# Inhibiting Histone and DNA Methylation Improves Cancer Vaccination in an Experimental Model of Melanoma

**DOI:** 10.3389/fimmu.2022.799636

**Published:** 2022-05-12

**Authors:** Lien De Beck, Robin Maximilian Awad, Veronica Basso, Noelia Casares, Kirsten De Ridder, Yannick De Vlaeminck, Alessandra Gnata, Cleo Goyvaerts, Quentin Lecocq, Edurne San José-Enériz, Stefaan Verhulst, Ken Maes, Karin Vanderkerken, Xabier Agirre, Felipe Prosper, Juan José Lasarte, Anna Mondino, Karine Breckpot

**Affiliations:** ^1^ Laboratory for Molecular and Cellular Therapy, Department of Biomedical Sciences, Vrije Universiteit Brussel (VUB), Brussels, Belgium; ^2^ Laboratory of Hematology and Immunology, Department of Biomedical Sciences, Vrije Universiteit Brussel (VUB), Brussels, Belgium; ^3^ Lymphocyte Activation Unit, Division of Immunology, Transplantation and Infectious Diseases, IRCCS Ospedale San Raffaele, Milan, Italy; ^4^ Immunology and Immunotherapy Program, Centro de Investigación Médica Aplicada (CIMA), Instituto de Investigación Sanitaria de Navarra (IdiSNA), Universidad de Navarra, Pamplona, Spain; ^5^ Hemato-Oncology Program, Centro de Investigación Médica Aplicada (CIMA), Instituto de Investigación Sanitaria de Navarra (IdiSNA), Universidad de Navarra, Pamplona, Spain; ^6^ Liver Cell Biology Research Group, Department of Biomedical Sciences, Vrije Universiteit Brussel (VUB), Brussels, Belgium; ^7^ Center for Medical Genetics, Vrije Universiteit Brussel (VUB), Universitair Ziekenhuis Brussel (UZ Brussel), Brussels, Belgium; ^8^ Laboratory of Cancer Epigenetics, Centro de Investigación Biomédica en Red de Cáncer (CIBERONC), Madrid, Spain; ^9^ Hematology and Cell Therapy Department, Clínica Universidad de Navarra, Universidad de Navarra, Pamplona, Spain

**Keywords:** melanoma, cancer vaccination, dendritic cell vaccination, adoptive T cell therapies, epigenetic targeted therapy, histone and DNA methylation/demethylation, histone methyltransferase G9a, DNA methyltransferase (DNMT)

## Abstract

Immunotherapy has improved the treatment of malignant skin cancer of the melanoma type, yet overall clinical response rates remain low. Combination therapies could be key to meet this cogent medical need. Because epigenetic hallmarks represent promising combination therapy targets, we studied the immunogenic potential of a dual inhibitor of histone methyltransferase G9a and DNA methyltransferases (DNMTs) in the preclinical B16-OVA melanoma model. Making use of tumor transcriptomic and functional analyses, methylation-targeted epigenetic reprogramming was shown to induce tumor cell cycle arrest and apoptosis *in vitro* coinciding with transient tumor growth delay and an IFN-I response in immune-competent mice. In consideration of a potential impact on immune cells, the drug was shown not to interfere with dendritic cell maturation or T-cell activation in vitro. Notably, the drug promoted dendritic cell and, to a lesser extent, T-cell infiltration in vivo, yet failed to sensitize tumor cells to programmed cell death-1 inhibition. Instead, it increased therapeutic efficacy of TCR-redirected T cell and dendritic cell vaccination, jointly increasing overall survival of B16-OVA tumor-bearing mice. The reported data confirm the prospect of methylation-targeted epigenetic reprogramming in melanoma and sustain dual G9a and DNMT inhibition as a strategy to tip the cancer-immune set-point towards responsiveness to active and adoptive vaccination against melanoma.

## Introduction

Melanoma is a malignant skin cancer with an estimated worldwide increase of 57% and 68% in the number of new cases and deaths by 2040, respectively. Despite a four-fold lower incidence compared to non-melanoma skin cancers, melanoma accounts for half of skin cancer-related deaths (1). Conventional therapies include surgery and chemo-radiotherapy, while targeted therapies and immunotherapy represent novel treatment options. Immunotherapy has gained attention owing to the potential of melanoma-specific cytotoxic T cells to kill melanoma cells irrespective of their location while ensuring long-term protection (2). Immune checkpoint blockade has indeed become standard-of-care for melanoma (3, 4), while active (5) and adoptive (6) vaccination have shown promising results in clinical trials. The yet low overall clinical response rates have prompted research on combination strategies.

Epigenetic modifying drugs pledge promising, as epigenetic events shape cell transformation of both cancer cells and cancer-supportive cells within the tumor micro-environment. Aberrations in both histone and DNA methylation patterns, in part due to histone methyltransferase G9a (7, 8) and DNA methyltransferase (DNMT) 1/3b overexpression (9, 10), have indeed been identified in melanoma. G9a promotes gene expression *via* monomethylation of histone 3 on lysine 9 (H3K9me1) (11). G9a together with DNMT1 represses gene expression by dimethylation of histone H3 on lysine 9 (H3K9me2) and DNA cytosine methylation (5mC), respectively (12). Together with *de novo* methylation implemented by DNMT3b, these processes cooperate to govern cellular integrity and to commit cells to a specific expression profile (13). Distinct promotor CpG hypermethylation patterns in melanoma patients have been recently identified to drive tumor immune cell exclusion, thereby linking aberrant methylation patterns to melanoma immune evasion, and as such suggestive of a correlation between prognosis and epigenetic immune regulation (14). The involvement of these epigenetic processes in melanoma initiation and progression renders them valuable targets for combined inhibition in the melanoma context (15–25).

CM-272 is a dual G9a/DNMT inhibitor with proven efficacy in hematological and solid cancer models. The drug inhibited tumor growth while promoting immunogenic cell death and IFN responses (26–28), as such facilitating synergism with programmed death ligand-1 (PD-L1) blockade in a preclinical bladder cancer model (28). This brings forth CM-272 as a prime candidate for combination with immunotherapy in melanoma.

To evaluate the combination of CM-272 with immunotherapy, we exploited the B16-OVA (MO4) melanoma model, which expresses ovalbumin (OVA) as a model antigen (29). This enables the evaluation of tumor/OVA-targeted active and adoptive immunotherapy. This model is representative of BRAF wild-type patients lacking p16^Ink4a^ and p14^Arf^ tumor suppressor proteins (29–33). From an immunological perspective, it also represents the so-called immunotype B patients, existing among both primary and metastatic melanoma patients that have a limited number of tumor-infiltrating lymphocytes, which has been identified as a poor prognostic factor (34–36). This compromised immune set-point manifests in the MO4 model as an inherent resistance to immune checkpoint blockade therapy (37–40). These features render this preclinical model relevant for evaluating more powerful combination strategies.

## Materials and Methods

### Mice, Cell Lines and Primary Cell Culture

Female 6-12 week old C57Bl6J, Crl:NU-Foxn1^nu^ or C57BL/6-Tg^(TcraTcrb)1100Mjb/J^ (OT-I) mice were purchased from Charles River (Saint-Germain-Nuelles, France; Calco, Italy). C57Bl6J mice for programmed cell death-1 (PD-1) blockade therapy were purchased from Harlan (Barcelona, Spain).

Cells were cultured at 37°C under humidified 5% CO_2_ atmosphere and tested negative for mycoplasma using VenorGeM Classic and MB Taq Polymerase (Minerva Biolabs, Berlin, Germany). MO4 cells were gifted by Ken Rock (Division of Lymphocyte Biology, Dana Farber Cancer Institute, Boston, Massachusetts; Department of Pathology, Harvard Medical School, Boston, Massachusetts), authenticated by Eurofins Scientific (Luxemburg, Belgium), and cultured in DMEM (Sigma-Aldrich, Overijse, Belgium) supplemented with 10% fetal bovine serum (TICO, Amstelveen, The Netherlands), 2mM L-Glutamine (Sigma-Aldrich), 100U/mL penicillin (Sigma-Aldrich), and 100µg/mL streptomycin (Sigma-Aldrich).

Dendritic cells (DCs) were generated from bone marrow cells of C57Bl6J mice, matured with lipopolysaccharide and pulsed with OVA_257-264_, as previously described (41). Where indicated, CD4+ or CD8+ T cells were isolated from C57Bl6J, OT-I or OT-II TCR transgenic mice. OVA-specificity was inherent to OT-I (OVA-derived SIINFEKL [OVA257-264] in H2-kb) and OT-II (OVA-derived ISQAVHAAHAEINEAGR [OVA323-339] in I-Ab) T cells, or was genetically engineered. For T cell isolation, spleens were isolated and passed through a 40µm cell strainer (Corning, New York, New York) before red blood cells were lysed. Single cell suspensions were subsequently enriched for the CD8^+^ fraction using negative MACS-selection (from OT-I mice for DC co-culture assays), or for the CD4^+^ or CD8^+^ fraction using negative and positive selection (from C57Bl6J, OT-I or OT-II mice for *in vitro* T cell sensitivity assays), according to manufacturer instructions (Miltenyi Biotec, Gladbach, Germany). For *in vitro* experiments on MO4-mediated T-cell stimulation, OVA-specific T cells were genetically engineered. To this end, a retrovirus encoding the OT-I TCR was produced as previously described (42), and used to transduce Concanavalin A/IL-7-activated mouse splenocytes by spin infection in retronectin (Takara)-coated plates (43).

### Therapeutic Reagents

CM-272 was developed at Clinica Universidad de Navarra (26), dissolved at 10mM in DMSO (Sigma-Aldrich), and diluted in culture medium (*in vitro)* or 0.9% NaCl infusion solution (*in vivo*) (Baxter, Lessines, Belgium). Combination therapy included: (1) anti-PD-1 (clone RMPI-14) or isotype-matched antibody (BioXcel, New Haven, Connecticut) at 0.5mg/mL in 0.9% NaCl-solution, (2) 1x10^6^ OT-I TCR-engineered or untransduced T cells, (3) 5x10^6^ DCs/mL in phosphate buffered saline (PBS) or PBS (Sigma-Aldrich).

### Experimental Set-Up - *In Vivo* Experiments

C57Bl6J mice were subcutaneously injected in the flank with 3x10^5^ MO4 cells in 50µL PBS. Treatment regimen was started on day 3 (unless stated otherwise), as an intraperitoneal CM-272 (5mg/kg)/vehicle injection for 5 consecutive days a week, until 1000 mm^3^ tumor volume endpoint was reached. Combination treatment included: (1) 3 intraperitoneal injections of 50µg anti-PD-1/isotype once a week starting from day 1 of treatment cycle 1 (day 7-10); (2) 1x10^6^ OT-I TCR-engineered/untransduced T cells (day 13). Treatment cycle 1 started on day 5; (3) 3-4 intravenous injections of 5x10^5^ DCs/vehicle once a week, starting from day 1 of treatment cycle 1. Tumor volume was measured 3-5 times per week and calculated as: (length x width^2^)/2, width being the smallest value. The ethical endpoint of the experiment allowed a maximum tumor volume of 1500 mm^3^. For evaluation of therapy efficacy, we plotted the time to reach a volume of 1000 mm^3^ (experimental endpoint) in a Kaplan-Meier curve, using an algorithm build on the following criteria. If on the day of monitoring the tumor volume reached 1000 ± 50 mm^3^, this day was plotted as experimental endpoint (criterium a). If (a) was not met, the day at which the tumor volume reached a volume closest to 1000 ± 150 mm^3^ was used (criterium b). If (b) was never met, the first day at which the tumor volume exceeded 1150 mm^3^ was used (criterium c). In case tumor volumes remained <850 mm^3^, mice were censored, i.e., scored as ‘alive’ (criterium d). Censoring was required when mice had to be taken out of the experiment for ethical reasons, e.g., ulcers of tumors combined with physical signs of declined health status. Outlier removal analysis was subsequently performed. The time to reach 1000 mm^3^ was plotted until the last mouse in the vehicle group reached this endpoint. With regard to tumor growth curves, mean tumor volume in time was plotted for each experimental group, until the first mouse of the concerning group had reached the experimental endpoint tumor volume.

For *ex vivo* tumor tissue analysis, tumors were processed to single cell suspensions either by application of the GentleMACS isolation protocol (Miltenyi Biotec) in case of downstream flow cytometry (at experimental endpoint), or by immediate lyses in case of downstream multiplex analysis (at 706.9 ± 194.8 mm^3^).

### Experimental Set-Up - *In Vitro* Sensitivity MO4 Cells

Quantification of epigenetic marks was performed as previously described (28), upon 48 hours (H3K9me2) or 5 days (5mC) exposure to 1.9μM CM-272. Furthermore, 1x10^4^ MO4 cells were exposed to 0.05-1μM CM-272 in 200μL in a flat-bottom 96-well plate (Sarstedt, Nümbrecht, Germany). Confluence, cytotoxicity and apoptosis were monitored with the IncuCyte Zoom (Essen BioScience, Welwyn Garden City, UK), and the number of viable cells was determined with CellTiter-Glo, as instructed (Promega, Leiden, The Netherlands). IC50 value was determined based on four-parameter nonlinear regression of vehicle-normalized CellTiter-Glo data. Concerning RNA sequencing and validation, 5x10^5^ MO4 cells were exposed to 0.05-1μM CM-272 in 5mL in a 6-well plate (Corning) for indicated timeframe. Cells were harvested for flow cytometry, snap-frozen awaiting western blot analysis, or processed for RNA sequencing.

### Experimental Set-Up - *In Vitro* Sensitivity T Cells

Purified CD4^+^/CD8^+^ or OT-I/-II T cells were labelled with carboxyfluorescein diacetate succinimidyl ester (CFSE) (Becton Dickinson [BD], Franklin Lakes, New Jersey) and stimulated with 0.5µg/mL plate-coated anti-CD3 (clone 145-2C11; Biolegend, San Diego, California) and 1µg/mL soluble anti-CD28 (clone 37.51; Biolegend), or 10µg/mL OVA-derived peptides (AnaSpec, Fremont, California) for 3 days, respectively. Non-mitogenic IL-7 (5ng/mL) served as a negative control (PeproTech, Cranbury, New Jersey). T cells were treated with 0.125-1μM CM-272. Cells were collected for flow cytometry at 72 hours and IFN-γ was measured in culture supernatants at 48 hours. MO4 cells were co-cultured for 48 hours with OT-I TCR-engineered T cells at 1:2 effector/target ratio, while exposed to 0.125-0.5μM CM-272, before IFN-γ measurement.

### Experimental Set-Up - *In Vitro* Sensitivity DCs

5x10^5^ DCs were exposed to 0.05-1μM CM-272 for 24 hours in a 48-well plate in 500μL complete RPMI-1640 (Sigma-Aldrich) and cultured for an additional 24 hours with/without 1μg/mL lipopolysaccharide (E. coli serotype O55:B5; Sigma-Aldrich). DCs were collected for flow cytometry and IL-12p70 was measured in culture supernatants. DCs pulsed with 10μg/mL OVA_257-264_ were co-cultured at 1:10 ratio with OT-I T cells for 72 hours before IFN-γ measurement. Unstimulated and CD3/CD28-stimulated (ThermoFisher, Waltham, Massachusetts) T cells served as negative and positive controls, respectively.

### RNA Extraction From MO4 Cells or *Ex Vivo* Tumor Tissue

Upon cell harvesting, cell integrity was evaluated using cell cycle analysis and sub-G1-phase quantification. MO4 cells in the sub-G1 phase amounted to 1.78% ( ± 0.67 SD) and 6.92% (± 2.32 SD) in vehicle and CM-272 treatment conditions, respectively. RNA was extracted from *in vitro* treated MO4 cells or *ex vivo* tumor tissue using the RNeasy plus mini kit, according to manufacturer instructions (Qiagen, Hilden, Germany). RNA quality control was based on the RNA integrity number score and DV200 score as determined on the 2100 Bioanalyzer (Agilent, Santa Clara, California). The RNA integrity number ranged from 8.7 to 7.1 and the DV200 score from 90 to 95%, indicating that the RNA was of sufficient (undegraded) quality to perform RNA sequencing on. Concentration was determined using Qubit RNA HS Assay (Invitrogen, Carlsbad, California). RNA was subsequently used for RNA sequencing (*in vitro* MO4 cells) or multiplex analysis (*ex vivo* tumor tissue).

### RNA Sequencing Analysis on MO4 Cells

**Figure 1:**
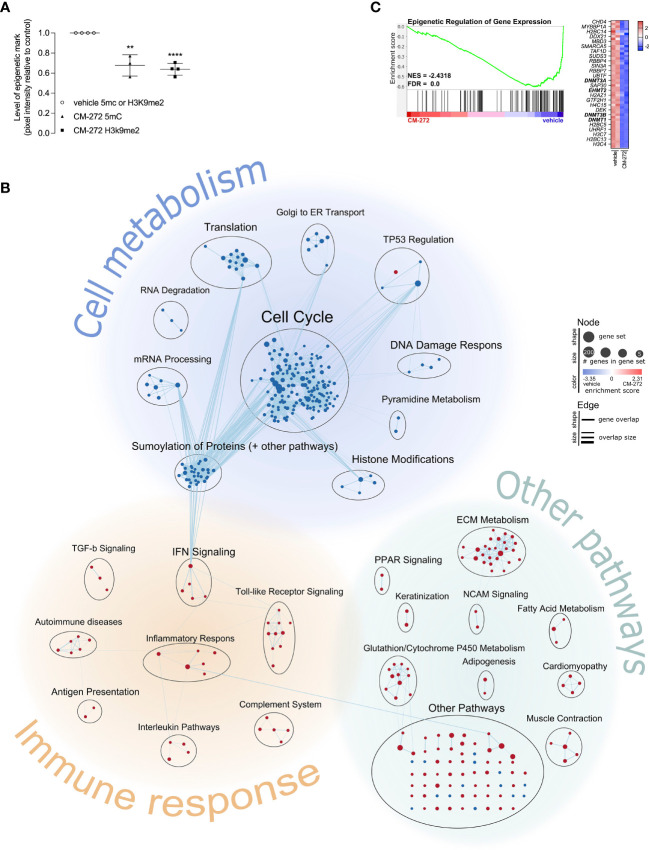
Dual G9a and DNMT inhibition shapes the transcriptional profile of melanoma cells, impacting on cell cycle progression and immunogenicity. **(A)** H3K9me2- and 5mC-levels upon 1.9μM CM-272 treatment (2 or 5 days respectively), relative to vehicle-treated cells (mean ± SD; *n*=3/4). **(B, C)** Gene expression changes in MO4 cells upon treatment with 1μM CM-272 for 24 hours (*n*=1, 2 s.p.c.). The percentage of cells with fractionated DNA (sub-G1-phase, indicative of cell death) amounted to 1.78 ± 0.67 and 6.92 ± 2.32 (SD) in vehicle and CM-272 treated conditions, respectively. **(B)** Graphical representation of GSEA on gene expression changes in MO4 cells. **(C)** Enrichment plot of a gene set involving epigenetic regulation of gene expression. Heatmap lists row-normalized gene expression of leading-edge genes. Vehicle and CM-272 conditions were compared using unpaired two-tailed student *t*-test **(A)**. Asterisks indicate statistical significance: **p ≤ 0.01; ****p ≤ 0.0001.

MO4 cells were treated for 24 hours with 1µM CM-272 or vehicle, and further processed as to extract the RNA. 150ng RNA per condition was used to construct an RNA library upon ribosomal RNA depletion using the KAPA Ribo Erase (HMR) kit (Kapa Biosystems, Basel, Switzerland), followed by sequencing on the Illumnia NovaSeq 6000 (Illumnia, San Diego, California). Gene expression counts were generated upon read alignment against the mus musculus reference genome version GRCm38-83 using STAR software (44), and subsequent analysis using HTSeq script in Python (45). Normalized gene expression counts and log_2_-fold change of gene expression were generated using DESeq2 script in R. Genes, as calculated by DESeq2 on single-gene level, in compliance with *p*-value <0.0005, *q*-value <0.002, and |log_2_-fold change| >1 were listed (**Supplementary Table 1**). Principal component analysis plot comparing vehicle and CM-272 samples was provided (**Supplementary Figure 1C**). Normalized counts from DESeq2 analysis were subjected to gene set enrichment analysis (GSEA) making use of GSEA v4.1.0 software and the Canonical Pathways (KEGG, PID, REACTOME and WikiPathways) gene set collections from the Molecular Signatures Database, as previously described (46–48). As such, gene sets were pre-filtered to a minimum of 15 and maximum of 500 number of genes, rendering 1776 gene sets (composed of 17870 gene markers) out of 2523 to be evaluated. Using 17870 gene markers for the CM-272 versus vehicle comparison, 1255 and 521 gene sets were identified as up- or downregulated in CM-272 condition, respectively. Significantly changed gene sets were defined as nominal p-value <0.005, *q*-value <0.1, |Normalized Enrichment Score (NES)| >1 (**Supplementary Table 2**). Results from **Supplementary Table 2** were visually presented using the EnrichmentMap Cytoscape application, as previously described (**Figure 1B**) (49).

### Multiplex Analysis on *Ex Vivo* Tumor Tissue

Tumors were resected from 6 mice (total 12) treated with CM-272 or vehicle, and further processed as to extract the RNA. RNA of each individual tumor was then analyzed with the nCounter PanCancer Mouse Immune Profiling Panel on the nCounter MAX Analysis System (Nanostring, Seattle, Washington). Quality control was performed using nSolver software. Expression counts (transcripts per million) were normalized making use of the Nanostring analysis-adjusted RuvSeq method (50). Principal component analysis plot comparing vehicle and CM-272 samples was provided (**Supplementary Figure 3A**). Normalized counts were subjected to GSEA, using Canonical Pathways (BIOCARTA, KEGG, PID, REACTOME and WikiPathways) gene set collections, as previously described (46–48). As such, gene sets were pre-filtered to a minimum of 15 and maximum of 500 number of genes, rendering 106 gene sets out of 2871 to be analyzed. Using 359 gene markers for the CM-272 versus vehicle comparison, 70 and 36 gene sets were identified as up- or downregulated in CM-272 condition, respectively. Significantly changed gene sets were defined as nominal *p*-value <0.005; FDR-value <0.1; |NES| >1 (**Supplementary Table 3**) and visualized using the EnrichmentMap Cytoscape application (**Figure 4D**). Tumor-infiltrating leukocyte (TIL)-scoring was performed in R as previously described (51), using cell type specific marker genes as specified by the PanCancer Mouse Immune Profiling Panel (Nanostring). Briefly, cell scores were calculated as the mean of log_2_-normalized gene expression value of all marker genes. Total TIL score per sample were calculated as the mean of all cell scores whose correlation with CD45 exceeded 0.6. Cell type enrichment score was calculated as the residual from the linear regression curve simulating cell score from total TIL score, combining all samples data for each cell type separately.

### IncuCyte Zoom Live Cell Imaging

The IncuCyte Zoom live cell imaging device was used to monitor cell confluence, cytotoxicity - 1:800 dilution of Incucyte Cytotox Red Dye (Sartorius, Göttingen, Germany), and expression of caspase-3/7 – 1:1500 dilution of Incucyte Caspase-3/7 Dye, according to manufacturer instructions (Sartorius).

### ELISA

Supernatant was collected from cell culture of DCs, T cells, DC/T cell co-cultures, or MO4/T cell co-cultures at indicated time-points. IL-12p70 and IFN-γ levels were measured according to manufacturer instructions (Invitrogen, BD Pharmingen).

### Flow Cytometry

Antibody staining was performed in 0.02% sodium azide/1% PBS supplemented with bovine serum albumin (prepared in-house), unless stated otherwise, for 1 hour at 4°C. Cells were acquired on the LSR Fortessa/Canto (BD) and data was analyzed with FlowJo v10 software (BD). Forward- and side-scatter properties were used to gate-out debris and aggregating cells before viable cells were selected using a viability dye. Cell cycle analysis included dead cells. MO4 cells were analyzed for: (1) cell cycle distribution: 3x10^5^ cells in 500μL PBS were fixated by addition to 4,5mL of a -20°C pre-cooled 70% Ethanol solution while vortexing. After 2 hours of incubation at -20°C, cells were washed twice and rehydrated for 15 minutes in PBS. DNA was stained by 10 minutes incubation with 200μL propidium iodide solution: 1 mg/mL sodium nitrate (Merck KGaA, Darmstadt, Germany), 0.1% Triton-X (Merck), 100 µg/mL RNase A (Boehringer, Ingelheim, Germany), and 50 µg/mL propidium iodide (Sigma-Aldrich). Gating strategy was provided (**Supplementary Figure 1F**). (2) Expression of SIINFEKL/H-2K^b^ (phycoerythrin [PE], clone eBio25-D1.16; eBioscience, San Diego, California) and PD-L1 (brilliant violet 421 [BV421], clone MIH5; Novus, Centennial, Colorado). The DC phenotype was analyzed based on surface expression of: CD11c (peridinin chlorophyll protein cyanine 5.5 [PerCP-Cy5.5], clone N418; Biolegend), CD40 (PE-cyanine 7 [PE-Cy7], clone 3.23; Biolegend), CD80 (BV421, clone 16-1OA1; BD), CD86 (fluorescein isothiocyanate [FITC], clone GL1; BD), I-A/I-E [I-A^d^] (allophycocyanin [APC], clone M5.114.15.2; Biolegend). Gating strategy was provided (**Supplementary Figure 4A**). *Ex vivo* tumor T-cell infiltrate of vehicle or CM-272-treated mice was analyzed based on: 7-AAD (Biolegend), PD-1 (PE, clone J43; BD), CD8a (Pacific Blue, clone 53-6.7; BD), CD4 (Alexa Fluor 700 [AF700], clone RM4-5; BD), CD3e (PE-Cy7, clone 17A2; Biolegend), CD45.2 (APC-eFluor 780 [APC-eFluor 780], clone 104; Invitrogen). *Ex vivo* tumor T-cell infiltrate of DC vaccine alone or DC vaccine and CM-272-treated mice was analyzed based on: Fixable viability dye eFluor506 (eBioscience), PD-1 (PE-Cy7, clone J43; Invitrogen), CD8a (Horizon v450, clone 53-6.7; BD), CD4 (alexa fluor 700 [AF700], clone RM4-5; BD), CD3e (PerCP-Cy5.5, clone 145-2C11; BD), CD45.2 (APC-cyanin 7 [APC-Cy7], clone 104; BD). For *ex vivo* tumor tissue analysis, cells were pre-stained with anti-CD16/32 (unconjugated, clone 93; Biolegend) and samples were fixed with Cytofix/cytoperm (BD), according to manufacturer instructions. Gating strategy was provided (**Supplementary Figure 4F**). T-cell proliferation was measured based on CFSE dilution in viable CD4^+^ or CD8^+^ T cells.

### Western Blot

Western blot-mediated quantification of epigenetic marks was performed as previously described (28). For the detection of p21 protein, MO4 cells were lysed in 400µL buffer (5% ß-mercaptoethanol laemmli buffer; prepared in-house) and boiled at 95°C for 10 minutes. 20µg of protein was size-separated on a SDS-PAGE gel next to size-reference (PageRuler; ThermoFisher) and transferred to a nitrocellulose membrane (Amersham, Little Chalfont, United Kingdom). The membrane was blocked in 5% low-fat milk TTBS (prepared in-house) before overnight incubation at 4°C with 5mL of 1:500 rabbit polyclonal IgG p21^Waf1/Cip1^ (clone C-19; SantaCruz, Dallas, Texas) or 1:1000 rabbit polyclonal ß-actin (Cell Signaling, Danvers, Massachusetts). Blots were incubated for 1 hour at room temperature with anti-rabbit horseradish peroxidase-linked IgG (Cell signaling). Proteins were detected using WesternBright chemiluminescent reagent (Advansta, San Jose, California), visualized on the Odyssey FC (LI-COR, Lincoln, Nebraska), and quantified relative to background making use of Image Studio Lite software (LI-COR).

### Statistical Analysis

Statistical analysis was performed using GraphPad Prism v9.1.0 or RStudio v1.3.1093. Outliers were selected using ROUT method at 0.1% (*in vivo*) or 1% (*in vitro/ex vivo*). Normality was tested using Shapiro-Wilk test. Sample sizes ≤ 4 were tested assuming normality. Asterisks or symbols indicate statistical significance: * p ≤ 0.05; ** p ≤ 0.01; *** p ≤ 0.001; **** p ≤ 0.0001. Only significant differences were indicated in graphs. Statistical tests, sample sizes (mice per condition [m.p.c.] or samples per condition [s.p.c.]), data variability (standard deviation [SD] or standard error of the mean [SEM]), and number of repeats (n) were indicated in figure legends.

## Results

### Dual G9a and DNMT Inhibition Results in Melanoma Cell Cycle Arrest and Cell Death

To study melanoma-intrinsic CM-272 effects, MO4 cells were first exposed to the drug *in vitro.* H3K9me2- and 5mC-levels were significantly reduced upon treatment, suggesting G9a and DNMT1 to be active in MO4 tumors and inhibitable by CM-272 (**Figure 1A**). Also, cell number and viability were significantly reduced by CM-272 doses above 0.25μM, reaching IC50 at 0.3844μM after exposure for 72 hours (**Supplementary Figures 1A, B**). These data show that MO4 cells are sensitive to epigenetic modulation by CM-272 *in vitro*.

To gain further insights and study transcriptional consequences, MO4 cells were treated for 24 hours with 1μM CM-272 and subjected to RNA sequencing. A total number of 1595 and 823 genes were identified as significantly up- or downregulated respectively in CM-272-treated MO4 cells, at single-gene level (**Supplementary Table 1**). Since the net consequence of single-gene expression changes in big data is generally considered difficult to assess, we made use of the GSEA method to evaluate net effect on pre-established signaling pathways (further referred to as ‘gene sets’). Significantly changed gene sets with associated gene set enrichment score and significance were listed in **Supplementary Table 2**. In addition, we visually represented significantly changed gene sets using the EnrichmentMap Cytoscape application (**Figure 1B**). Each significantly changed gene set is presented as a node, whose size and color indicated the number of associated genes and the associated enrichment score. Edges between nodes indicate gene overlap and overlap size. Biologically associated nodes are visually grouped and annotated with an appropriate term, indicating various biological processes. Finally, biological processes are also visually grouped according to whether they pertained to cell metabolism, immune response, or other biological pathways. Some relevant gene sets were further disclosed, showing normalized gene expression of leading-edge genes (**Figure 1C**) or of the top 20 up- and down-regulated genes (**Supplementary Figures 1D, E**). GSEA identified 218 and 100 gene sets as significantly down- or upregulated respectively in CM-272-treated MO4 cells (**Figure 1B** and **Supplementary Table 2**). Among the downregulated gene sets, those pertaining to cell metabolism, particularly cell cycle regulation, were most represented. In addition, CM-272 downregulated gene sets critical for cell functioning, such as mRNA processing, translation and degradation; the DNA damage response; protein SUMOylation; and epigenetic regulation, including DNMT1/3 (*dnmt1/3*) and G9a (*ehmt2*). (**Figure 1C**). Also, gene sets involving tumor suppressor 53 (TP53) regulation were downregulated, apart from one upregulated gene set, pointing toward cyclin-dependent kinase inhibitor protein p21 (*cdkn1a*)-induced cell cycle arrest and apoptosis (**Figure 1B**; **Supplementary Figure 1D**). GSEA also identified upregulation of gene sets linked to carcinogenesis (TP53 regulation, neural cell adhesion molecule signaling and extracellular matrix metabolism) and immune responses (antigen presentation, complement system, inflammatory response through IL-10 signaling, and IL-4/12/13/23/27, IFN-I/II, transforming growth factor-β [TGF-β] and toll-like receptor signaling) (**Figure 1B** and **Supplementary Figure 1E**).

**Figure 2:**
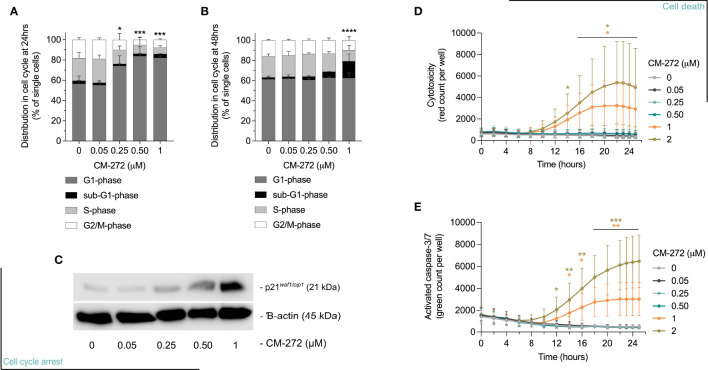
Dual G9a and DNMT inhibition causes MO4 cell cycle arrest and cell death. **(A, B)** Percentage of MO4 cells in cell cycle phases (mean ± SD; *n*=4). Asterisks represent significant differences in **(A)** G1-phase at 24 hours or **(B)** sub-G1-phase at 48 hours. **(C)** p21 protein expression and ß-actin loading control (representative blot; *n*=3). **(D, E)** Cell death induction: **(D)** Loss of cell membrane integrity and **(E)** caspase-3/7 activity in time (mean ± SD; *n*=3). Vehicle and CM-272 conditions were compared using ordinary one-way Anova and *post-hoc* Dunnett’s multiple comparison tests **(A, B)** or REML modeling with Geisser-Greenhouse correction and *post-hoc* Sidak multiple comparison test **(D, E)**. Asterisks indicate statistical significance: *p ≤ 0.05; **p ≤ 0.01; ***p ≤ 0.001; ****p ≤ 0.0001.

Next, we validated selected pathways possibly causing acute cytostatic or cytotoxic events. Using flow cytometry, CM-272 was shown to cause cells to arrest in the G1-phase of cell cycle by 24 hours of treatment in a dose-dependent manner (**Figure 2A**), and the accumulation of sub-G1 cells, representative of apoptotic cells with fractionated DNA, by 48 and 72 hours (**Figure 2B** and **Supplementary Figure 1G**) (52). At transcriptional level, the upregulation of cyclin D1 (*ccnd1*) at 24 hours accompanied the G1-arrest (**Supplementary Table 1**), along with that of *cdkn1a* (**Supplementary Table 1**) and concomitant accumulation of p21 (**Figure 2C** and **Supplementary Figure 1H**). These events were concomitant to a rise in cytotoxicity and caspase-3/7 activation, significantly detected at 1-2μM CM-272 (**Figures 2D, E**). These results suggest that CM-272-treated MO4 cells undergo cell cycle arrest followed by cell death. Notably, the finding that CM-272 concomitantly reduced cell growth while causing an upregulation of immune-related gene sets suggested that CM-272-treated tumors *in vivo* might develop a different sensitivity to immune recognition.

### Dual G9a and DNMT Inhibition Favors Tumor Cell Recognition by Tumor-Specific T Cells *In Vitro*

**Figure 3:**
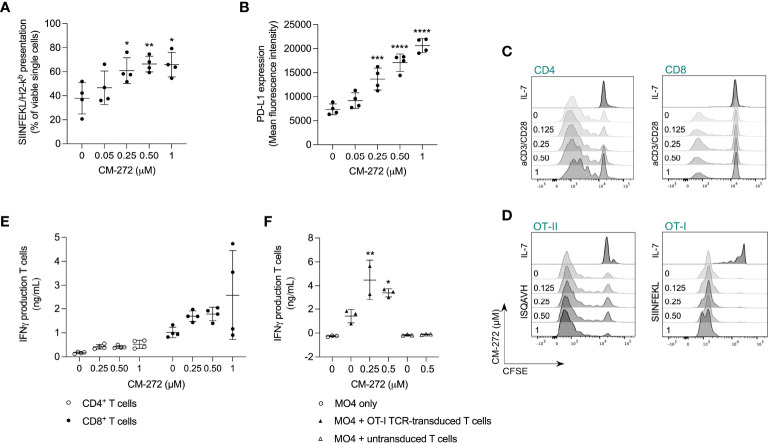
Dual G9a and DNMT inhibition favors tumor cell recognition by tumor-specific T cells in vitro. **(A, B)** MO4 cell surface presentation of **(A)** SIINFEKL/H2-K^b^ or **(B)** PD-L1 after CM-272 treatment for 24 hours (mean ± SD; *n*=4). **(C, D)** T-cell proliferation upon 72 hours of polyclonal (anti-CD3/CD28) or peptide (SIINFEKL/ISQAVH) stimulation of **(C)** CD4^+^ and CD8^+^ T cells or **(D)** OT-I and OT-II T cells, respectively, in the presence or not of CM-272 (representative histograms; *n*=4 [B]/*n*=2 [C]). **(E, F)** IFN-γ production upon 48 hours of **(E)** polyclonal stimulation of T cells (mean ± SD; *n*=2, total 4 s.p.c.) or **(F)** MO4-mediated stimulation of (un)transduced T cells (mean ± SD; *n*=2/3). Vehicle and CM-272 conditions were compared using ordinary one-way Anova and *post-hoc* Dunnett’s multiple comparison test (A,B,F). Asterisks indicate statistical significance: *p ≤ 0.05; **p ≤ 0.01; ***p ≤ 0.001; ****p ≤ 0.0001.

To address effects on tumor/T-cell recognition, we first investigated if CM-272 promoted MO4 antigen presentation. SIINFEKL/H-2K^b^ complexes were found to be upregulated by CM-272 in a dose-dependent manner (**Figure 3A**). Notably, CM-272 also increased PD-L1 expression (**Figure 3B**), questioning net effects on T-cell activation.

We also investigated putative T-cell intrinsic effects of CM-272. T-cell expansion to polyclonal (**Figure 3C**; **Supplementary Figure 1I**) and antigen-driven (**Figure 3D**) stimulation as well as IFN-γ production (**Figure 3E**) were comparable in the absence or presence of CM-272. In addition, in MO4 and OVA-specific TCR-engineered T cell co-cultures, IFN-γ production (**Figure 3F**) was significantly increased upon CM-272 exposure. These results indicate that CM-272 promotes tumor/T cell recognition.

### Dual G9a and DNMT Inhibition Transiently Delays Melanoma Growth in Immune-Competent Mice

**Figure 4:**
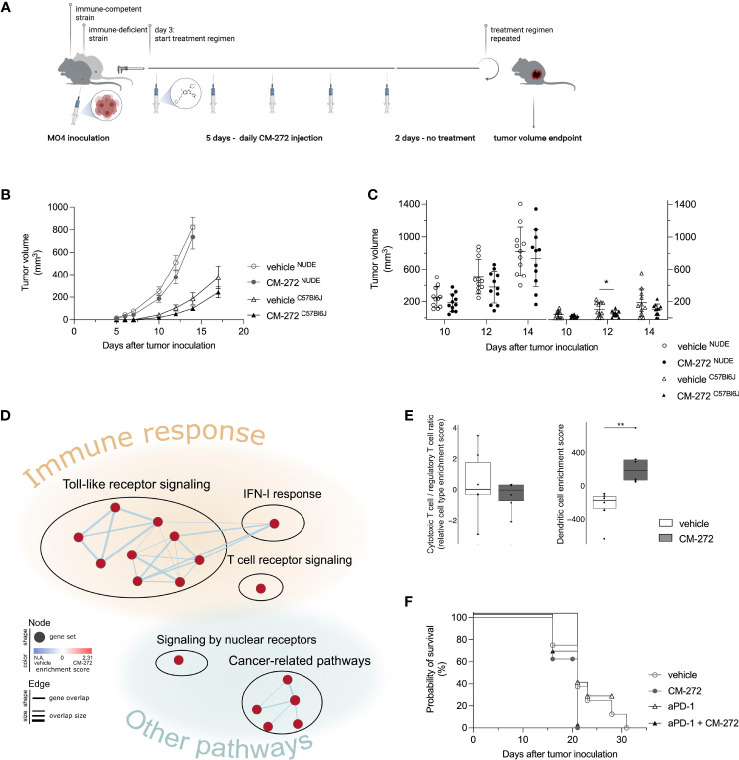
Dual G9a and DNMT inhibition transiently delays MO4 tumor growth in immune-competent mice. **(A, C)** CM-272 therapy in immune-deficient (NUDE) and -competent (C57Bl6J) MO4-bearing mice. **(A)** Schematic representation of treatment regimen (*n*=1, 11 m.p.c.). **(B, C)** Tumor volume in time (mean ± SEM [B]/mean ± SD [C]). **(D, E)** Multiplex tumor analysis (706.9 ± 193.8 mm^3^; *n*=1, 12 m.p.c.). **(D)** Graphical representation of GSEA. **(E)** TIL-scoring (10-90 percentile Box&Whiskers). **(F)** Mice survival (Kaplan-Meier curve) upon CM-272 combination with PD-1 blockade (aPD-1; *n*=1, 6-8 m.p.c.). Vehicle and CM-272 conditions were compared using REML modeling and *post-hoc* Sidak multiple comparison test **(B)**; unpaired one-tailed student *t*-test with Welsh correction or Mann-Whitney test **(C)**; Wilcoxon rank sum test **(E)**; Log-rank test **(F)**. Asterisks indicate statistical significance: *p ≤ 0.05; **p ≤ 0.01.

CM-272’s therapeutic activity was evaluated in immune-competent and -deficient mice bearing subcutaneous MO4 tumors. CM-272 or vehicle were injected intraperitoneally starting at day 3, and tumor growth was monitored in time (**Figure 4A**, treatment scheme). Of note, MO4 tumors developed faster in immune-deficient mice compared to immune-competent ones (**Figure 4B** and **Supplementary Figure 2A**). CM-272 delayed tumor growth only in immune-competent mice and only transiently. Indeed, effects were most evident at day 12 (**Figure 4C**) and omitted to significantly impact survival (data not shown). As the tumor delay effect of CM-272 *in vivo* was prone to variability (50% efficacy across 6 independent experiments, conducted at independent sites), further investigation into transcriptional reprogramming by CM-272 *in vivo* was deemed necessary. By extension thereof, these data support the possibility that, in context of melanoma, CM-272 exerts anti-tumor activity mainly *via* immune-mediated mechanisms.

To address this, we performed multiplex gene expression analysis on MO4 tumors from immune-competent mice to study the tumor immune-contexture. CM-272 upregulated toll-like receptor and IFN-I signaling, as identified by GSEA (**Figure 4D**). Although this corroborated *in vitro* findings on CM-272’s ability to fuel immune-signaling, at present we cannot discriminate whether *in vivo* effects are due only to tumor-intrinsic effects or also to effects on other tumor-infiltrating/resident cells. Enrichment of a TCR-signaling gene set was also observed, potentially reflecting the upward trend in (CD8^+^) T-cell representation (including cytotoxic and exhausted T cells as well as T helper 1 cells) and the downward trend in regulatory T cells, as identified by TIL-scoring (**Supplementary Figures 3B–D**). Although the CD8^+^ T cell/regulatory T cell ratio remained unchanged, DCs were significantly increased within CM-272-treated tumors (**Figure 4E**). These data support CM-272-driven immunomodulation *in vivo*.

### Dual G9a and DNMT Inhibition Promotes the Therapeutic Efficacy of DC Vaccination

Given that CM-272 impacts on antigenicity (SIINFEKL/H-2K^b^ complexes, **Figure 3A**) and immunogenicity (PD-L1, **Figure 3B**) *in vitro*, and on the immune-contexture *in vivo* (**Figures 4D, E**), we investigated possible cooperation with various immunotherapy strategies. We studied the combination of CM-272 with PD-1 blockade therapy (2.5mg/kg intraperitoneal, on day 7, 14 and 21), which we reasoned could counteract CM-272-induced PD-L1 upregulation and thereby ameliorate the narrow therapeutic window for PD-1/PD-L1 blockade in melanoma subsets (37–39, 53). However, no benefit was observed as PD-1 blockade failed to delay tumor growth when administered alone and in combination with CM-272 (**Figure 4F** and **Supplementary Figure 2B**).

**Figure 5:**
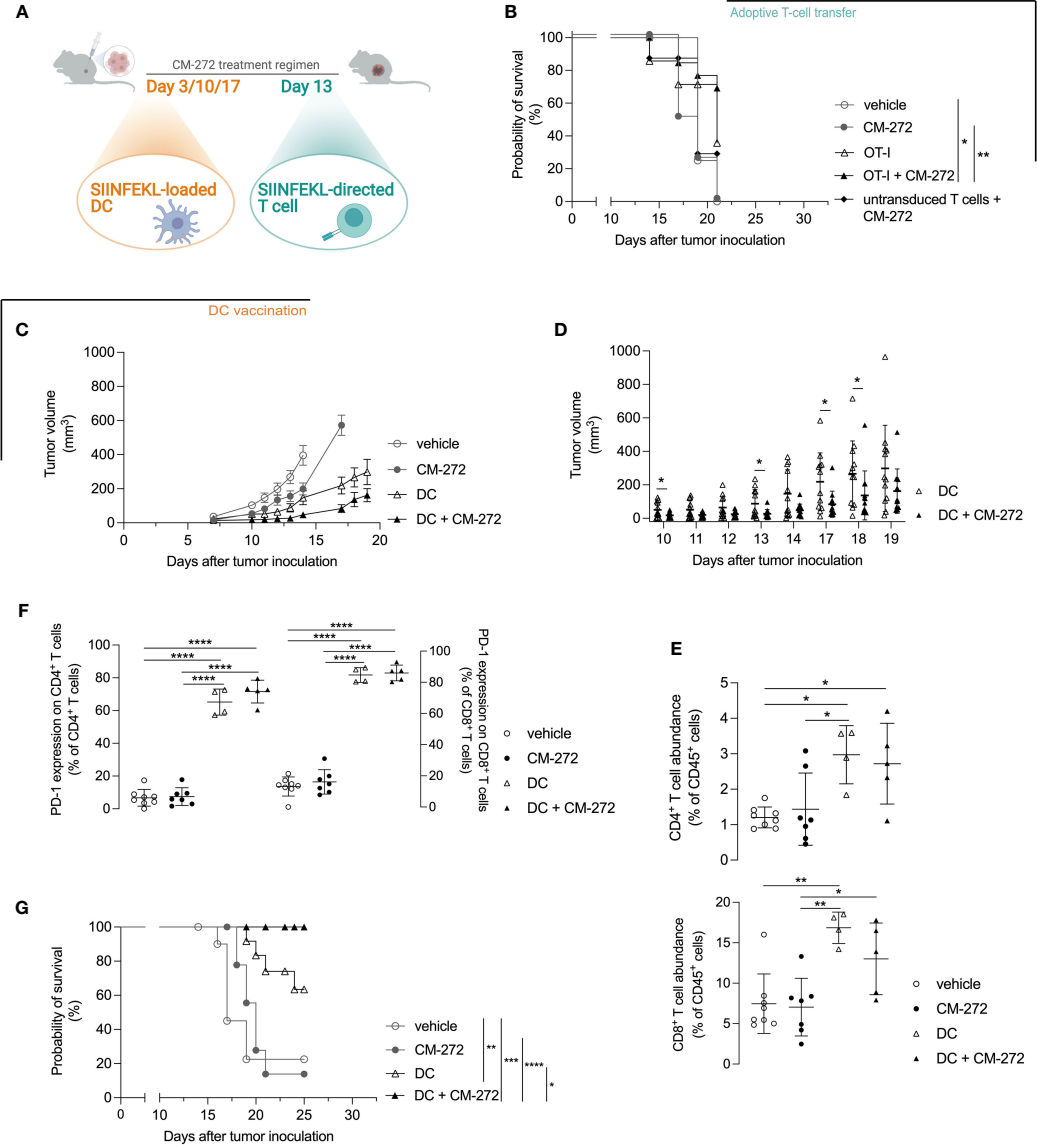
DC vaccination best cooperates with dual G9a and DNMT inhibition in prolonging mouse survival. **(A–G)** CM-272 combination therapy with **(B)** adoptive T-cell therapy (*n*=3, total 7-14 m.p.c.) or **(C–G)** DC vaccination (*n*=2, total 12 m.p.c.). **(A)** Treatment regimen. **(B, G)** Survival (Kaplan-Meier curve) upon CM-272 combination with **(B)** T-cell therapy or **(G)** DC vaccination. **(C, D)** Tumor volume in time (mean ± SEM [C]/mean ± SD [D]). **(E, F)** Tumor-contexture upon CM-272 combination with DC vaccination (*n*=1, 4-8 m.p.c.). **(E)** CD4^+^/CD8^+^ T-cell abundance. **(F)** PD-1 expression on CD4^+^/CD8^+^ T cells. Vehicle and experimental conditions were compared using Log-rank test (B,G); REML modeling with Geisser-Greenhouse correction and *post-hoc* Sidak multiple comparison test **(C)**; unpaired one-tailed student *t*-test or Mann-Whitney test **(D)**; ordinary one-way Anova and *post-hoc* Tukey’s multiple comparison test **(E, F)**. Asterisks indicate statistical significance: *p ≤ 0.05; **p ≤ 0.01; ***p ≤ 0.001; ****p ≤ 0.0001. Statistical significance of panel C is supplemented in **Table S4**.

We then reasoned that T-cell representation might be insufficient and therefore tested the combination with adoptive T-cell therapy in the form of SIINFEKL-specific TCR-engineered T cells (**Figure 5A**, treatment scheme). Provision of T cells improved the therapeutic effects of CM-272, allowing the survival of 69.2% of mice at the time all vehicle controls reached endpoint (survival proportion 0%) (**Figure 5B**). Yet, the combination of CM-272 and T-cell therapy was not sufficient for durable responses.

We thus reasoned that T-cell priming might be a limiting factor. We therefore investigated active DC-mediated vaccination as to better instigate tumor-directed CD8^+^ T-cell responses *in vivo*. DCs of bone marrow origin were matured with lipopolysaccharide and pulsed with SIINFEKL. Lipopolysaccharide-matured DCs expressed CD40, CD80 and CD86 co-stimulatory molecules (**Supplementary Figures 4A–C**) and secreted IL-12p70 (**Supplementary Figure 4D**) to comparable extent in the absence or the presence of CM-272. Likewise, mature DCs induced comparable IFN-γ secretion by SIINFEKL-specific CD8^+^ T cells (**Supplementary Figure 4E**). *In vivo*, DC vaccination delayed tumor growth compared to vehicle- and CM-272-only treatments (**Figure 5C**). Adding CM-272 to DC vaccination further increased tumor growth control. This was best found on day 10, 13, 17, and 18 (**Figures 5C, D**). Flow cytometry analysis of the tumor at end-stage confirmed that DC vaccination caused tumor infiltration by both CD4^+^ and CD8^+^ T cells, which remained unchanged in combination with CM-272 (**Figure 5E**). A significant fraction of T cells upregulated PD-1, indicative of acute tumor recognition (**Figure 5F**). The combination of CM-272 and DC vaccination best promoted mouse survival, with 100% of treated mice being alive at the time all vehicle mice had reached endpoint (survival proportion 22.5%), compared to 63.5% upon DC vaccination only (**Figure 5G**). These data indicate that the dual G9a and DNMT inhibitor CM-272 promotes the therapeutic effect of cancer vaccination against melanoma.

## Discussion

We report that the dual G9a and DNMT inhibitor CM-272 can be used in combination with adoptive T cell therapy and active cancer vaccination against melanoma. The data support the notion that both tumor cell-intrinsic and -extrinsic events shape *in vivo* responses to such combination therapies, and that counteractive effects might be concomitantly induced, possibly hindering full efficacy of such combined strategies.

*In vitro*, CM-272 caused MO4 cell cycle arrest and cell death. This corroborates recent findings that correlated G9a- and DNMT1-activity to melanoma cell proliferation (7–9). Also, restoring wild-type TP53 transcriptional activity, or at least tipping the balance away from oncogenic mutant isoforms, has been receiving attention as a means to tackle melanoma cell proliferation and therapy resistance (54). Our results advocate for epigenetic regulation of isoform expression, as CM-272 induced TP53 signaling toward p21-mediated cell cycle arrest and apoptosis. Though p21 effects are ambiguous, it is considered to mediate G1 arrest at high concentrations (55). This substantiates the anti-proliferative effect of CM-272 on MO4 cells. Notably, p21 upregulation has been shown to sensitize melanoma cells to T-cell cytotoxicity (56). Accordingly, we found improved recognition of CM-272-treated tumors by T cells *in vitro.*

We also found evidence of CM-272 having immune-modulatory consequences both *in vitro* and within the TME *in vivo*, suggesting that it might help shifting the cancer-immune set-point beyond the activation threshold (57). Indeed, in transcriptomic analyses we found that CM-272 induced the upregulation of several gene sets related to immune responses, including toll-like receptor and IFN-I signaling. We appreciate that IFN-I signaling could be due to MYD88 signaling resulting from epigenetic re-expression of retroviral elements or from the response to genetic material from dying cells (58), and operates as a bridge between innate and adaptive anti-tumor immunity. IFN-I has been previously shown to promote DC maturation and IL-12p70 production, instruct the local release of chemo-attractants for monocyte and lymphocyte recruitment, and induce antigen and co-stimulatory ligand expression, as such facilitating T cell reactivation and tumor recognition (59–62). Although not all transcriptional changes defined *in vitro* were validated in explanted tumors, we appreciate that they could all participate in establishing a more favorable immune contexture *in vivo*. Accordingly, CM-272 caused DC enrichment in the tumor, thus supporting the promise for synergistic activity in combination with immunotherapy. Nevertheless, it should be noted that CM-272 also caused PD-L1 upregulation on tumor cells, a known IFN-I feedback mechanism protecting tumors from IFN-mediated toxicity (63). In addition, tolerogenic signals including IL-10 (64) and TGF-β (65) were also upregulated by CM-272. GSEA thus identified CM-272’s epigenetic reprogramming as a putative *in vivo* double-edged sword. We believe that such dual activity is consistent with the transient therapeutic effects reported *in vivo*. The fact that CM-272 depends on an intact immune system to exert an anti-tumor effect suggests that the direct tumor inhibition observed *in vitro* is not as prominent *in vivo*. This may be due to insufficient drug penetration into the tumor site *in vivo* to confer direct tumor-cell intrinsic cytotoxic effects. Ensuing this notion, suboptimal CM-272 tumor-cell intrinsic effects *in vivo* could also explain its failure to confer a significant survival improvement when provided as a single agent. Regardless, *in vivo* tumor transcriptional changes upon CM-272 treatment suggest the drug to be active at the tumor site, and able to instigate a signaling cascade toward immune activation. In line with our statement above, we contend that the reported results reflect some tumor cells being affected by CM-272 *in vivo*, e.g., by cell death induction or cellular stress as observed *in vitro*, and this to be sufficient to initiate transcriptional events reflective of reprogramming of the tumor microenvironment and of immune-mediated destruction.

Since PD-L1 levels were augmented by CM-272, and DC influx and TCR-signaling (local T-cell activity) were induced by CM-272 *in vivo* administration, we first tested possible synergy with PD-1 blockade therapy. The decision to target PD-1 rather than PD-L1 was based on previous published results. Indeed, a meta-analysis of melanoma patients treated with PD-1 or PD-L1 blockade reported a 37% response rate in former compared to 16% in the latter (66). In addition, while anti-PD-1/PD-L1 blocking antibodies were proven similarly efficacious in B16 melanoma-bearing mice, even at elevated PD-L1 levels (37), the potency of anti-PD-L1 and not that of anti-PD-1 was found to decline with age in the B16 model (67). Thus, both for putative translational purposes and to avoid confounding effects, the synergy with anti-PD-1 was first investigated. We found that CM-272 failed to sensitize MO4 tumors to PD-1 blockade. This contradicts the successful combination of anti-PD-L1/CM-272 in a bladder cancer model (28) and of anti-PD-1/UNC0642 (G9a inhibitor) in the parental B16F10 model (68), as well as other preclinical reports on G9a or DNMT inhibition across different tumor models (8, 69, 70), and yet can be explained by diverse immune contextures or pharmacokinetics of epigenetic remodeling. In agreement with our findings, clinical trials have reported on a significant patient subgroup that does not respond to such combined treatments. While phase I/II clinical trials in melanoma patients testing DNMTi’s in combination with anti-CTLA-4 or anti-PD-1 mAbs are still ongoing (71), a phase II trial in acute myeloid leukemia reported on the combination of the DNMTi Azacitidine and PD-1 inhibitor Nivolumab. Here, despite an encouraging overall response rate of 33%, low pre-therapy tumor infiltration by T cells remained a limiting factor for therapeutic response (72). Still, we acknowledge that implementation of PD-1 blockade, albeit increased PD-L1 expression upon CM-272 treatment, could be subject to discussion. As stated before, the decision to target PD-1 was made to avoid confounding effects in the B16 model as well as for translational purposes, based on previous published results (37, 66, 67). Notwithstanding the grounds for testing PD-1 blockade, future studies might address the blockade of PD-L1 or of other immune checkpoints when of relevance. Also, using anti-PD-L1 as a single agent or in combination with anti-PD-1 might further improve therapeutic efficacy of the combined vaccination treatment. Future studies are needed to address this possibility.

On these grounds, and with the aim of promoting T-cell responses, we moved to adoptive T-cell therapy and active vaccination. Adoptive T-cell therapy showed capable of some cooperative effects in the MO4 model. Indeed, the combination of CM-272 and TCR-redirected T cells promoted longer survival in a significant fraction of tumor-bearing mice, although tumors eventually escaped control. Cooperative activity could be explained by the ability of CM-272 to promote direct peptide/MHC-complex presentation, rendering melanoma cells better targets for adoptively transferred effector T cells (73). Pursuant to this, we reasoned that CM-272-induced sustained IFN-I/II signaling and/or factors like IL-10 and TGF-β could indeed hinder tumor antigen presentation by tumor-resident DCs, thereby restraining successful combination therapy of CM-272 and PD-1 inhibition (60, 74–81). We also found *cd209* expression to be increased by CM-272, potentially reflecting monocyte-derived DCs, known to have paradoxical effects on T-cell responses (82). Directly improving *in situ* tumor antigen presentation by DCs should thus enable the generation of a successful anti-tumor T-cell response if indeed it is the limiting factor. To test this, we vaccinated mice with antigen-loaded mature DCs, known to promote protective immunity in preclinical models and clinical trials (38, 83). Combining CM-272 with DC vaccination prolonged tumor growth control and increased survival compared to individual therapies to extents that surmised those evoked by the combination of adoptive T-cell therapy and CM-272. We attribute this to the ability of mature DCs to express co-stimulatory ligands and to secrete IL-12p70, key for cytotoxic T-cell induction (84), and local reactivation of T cells combined with the cytotoxic and immune-shaping support from CM-272.

Thus, our work extends previous *in vitro* reports on increased melanoma antigenicity upon methylation-targeted epigenetic treatment in cancer vaccination context (73, 85) and underlines epigenetic reprogramming as a strategy to tip the cancer-immune set-point toward responsiveness to immunotherapeutic strategies. We expect additional studies to stem from this proof-of-principle report as to include the validation of the therapeutic robustness of this combined strategy when targeting unmutated tumor-associated self-antigens, the selection of the most appropriate vaccination platform (e.g., mRNA vaccination), and the definition of markers of epigenetic reprogramming capable of predicting sensitivity to the most appropriate immunotherapy.

## Data Availability Statement

The datasets presented in this study can be found in online repositories. The names of the repository/repositories and accession number(s) can be found below: Mendeley; 10.17632/x26nbkdjj3.1, 10.17632/pzvxcmhxb7.1, 10.17632/4zgd4ssrv2.1.

## Ethics Statement

All animal experiments were reviewed and approved by the Ethical Committee for Animal Experiments of the Vrije Universiteit Brussel (16-214-11 and 19-214-7); the Ethical Committee for Animal Experiments of the San Raffaele Scientific Institute (IACUC 858, Authorization 839/2017-PR); the Ethical Committee for Animal Experiments of the Universidad de Navarra (R-018-19).

## Author Contributions

LDB: conceptualization (equal), investigation (lead), data curation (lead), formal analysis (lead), project administration (equal), validation (equal), writing – original draft (equal), writing – review and editing (equal), visualization (lead), supervision (equal); KB: conceptualization (equal), funding acquisition (equal), project administration (equal), formal analysis (supporting), validation (equal), writing – original draft (equal), writing – review and editing (equal), supervision (equal); AM: conceptualization (equal), funding acquisition (equal), project administration (supportive), formal analysis (supporting), validation (equal), writing – review and editing (equal), supervision (equal); JJL: conceptualization (equal), Funding acquisition (equal), project administration (supporting), validation (supporting), writing – review and editing (equal), supervision (equal); XA: conceptualization (equal), funding acquisition (equal), writing – review and editing (supporting); FP: conceptualization (equal), funding acquisition (equal), writing – review and editing (supporting); CG: investigation (supporting), writing – review and editing (supporting); SV: software (equal), writing – review and editing (supporting); RMA: investigation (supporting); VB: investigation (supporting); NC: investigation (supporting); KDR: software (equal); YDV: investigation (supporting); AG: investigation (supporting); QL: investigation (supporting); ESJ-E: investigation (supporting); KM: writing – review and editing (supporting); KV: writing – review and editing (supporting). All authors contributed to the article and approved the submitted version.

## Funding

LDB, RMA, KDR, YDV, CG, QL, SV, KM, KV, and KB: This research was performed with financial support from the Vrije Universiteit Brussel (VUB) under the strategic research program scheme (SRP48) and from ERA-NET TRANSCAN 2 JTC 2015 (EPICA project; G0H7216N). LDB, RMA, YDV, QL, and SV received funding from het Fonds Wetenschappelijk Onderzoek - Vlaanderen (FWO) under the predoctoral FWO-FR research grant (1164918N), pre-doctoral FWO-SB research grants (1S53719N, 1S24817N, and 1S24218N), and the junior post-doctoral research grant (1243121N), respectively. C.G received funding from the research council of the Vrije Universiteit Brussel (VUB; OZR3458). FP, XA, and JJL: This research was performed with financial support from the Foundation for Applied Medical Research, the University of Navarra, Fundación Fuentes Dutor, Instituto de Salud Carlos III (ISCIII) and co-financed by FEDER (PI20/01308, PI20/01306), CIBERONC (CB16/12/00489), ERA-NET TRANSCAN-2 JTC 2015 (EPICA project; AC16/00041), Spanish Ministry of Economy, Industry and Competitiveness (RTHALMY SAF2017-92632-EXP), Ministerio de Ciencia e Innovación (PID2019-108989RB-I00), and Gobierno de Navarra (strategic projects DIANA, DESCARTHeS and AGATA). AM: This research was performed with financial support from ERA-NET TRANSCAN-2 JTC 2015 (EPICA project; AC16/00041) and Associazione Italiana per la Ricerca sul Cancro (AIRC IG 2014 Id.15883 and AIRC IG 2018 Id.21763).

## Conflict of Interest

The dual G9a/DNMT inhibitor CM-272 pertains to patent WO2015192981A1, on which XA, FP, and ESJ-E are filed as inventors.

The remaining authors declare that the research was conducted in the absence of any commercial or financial relationships that could be construed as a potential conflict of interest.

## Publisher’s Note

All claims expressed in this article are solely those of the authors and do not necessarily represent those of their affiliated organizations, or those of the publisher, the editors and the reviewers. Any product that may be evaluated in this article, or claim that may be made by its manufacturer, is not guaranteed or endorsed by the publisher.

## Abbreviations

5mC, cytosine methylation; BD, Becton Dickinson; CFSE, carboxyfluorescein diacetate succinimidyl ester; DC, dendritic cell; DNMT, DNA methyltransferase; GSEA, gene set enrichment analysis; H3K9me2, di-methylated lysine 9 on histone H3; m.p.c., mice per condition; NES, normalized enrichment score; OVA, ovalbumin; PD-1, programmed cell death-1; PD-L1, programmed death ligand-1; *q-*value, adjusted *p*-value; SD, standard deviation; SEM, standard error of the mean; s.p.c., samples per condition; TGF-β, transforming growth factor-β; TIL, tumor-infiltrating leukocyte; TP53, tumor suppressor 53.
