# Supplementary material for: Inhibiting Histone and DNA Methylation Improves Cancer Vaccination in an Experimental Model of Melanoma
Source: Front Immunol. 2022 May 12;13:799636. doi: 10.3389/fimmu.2022.799636 (PMC9134079; doi:10.3389/fimmu.2022.799636)
Supplement: Supplementary file 1 [file DataSheet_1.pdf]

SUPPLEMENTARY FIGURES

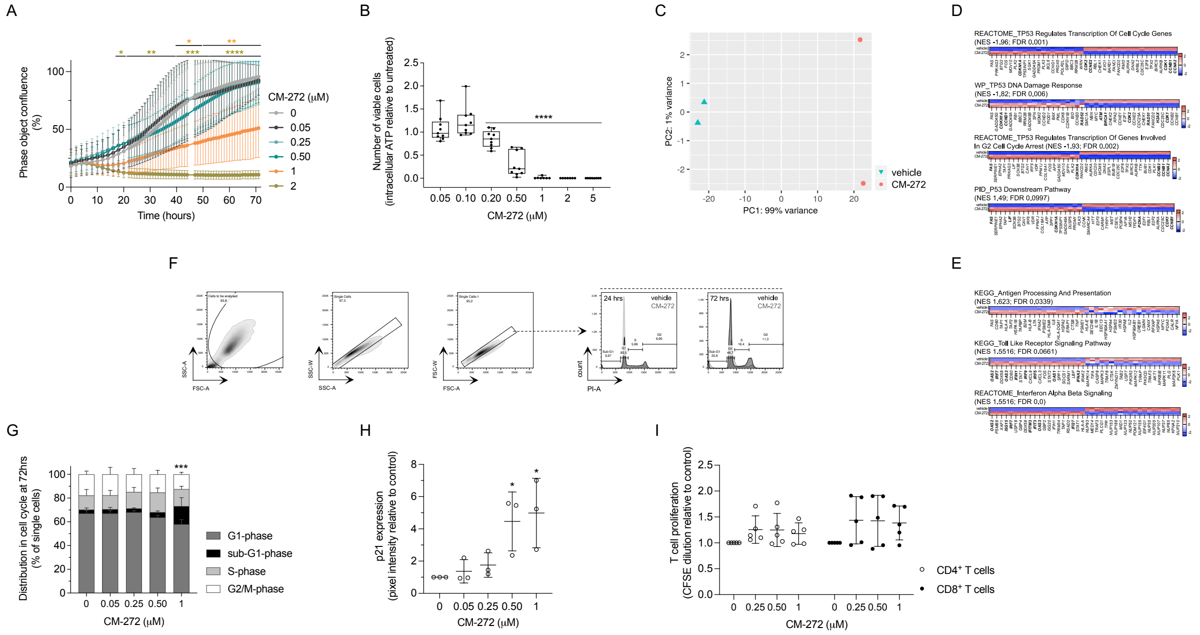

Figure S1 - Validation of CM-272 effect on MO4 cells and T cells in vitro. (**A-H**) CM-272 effect on MO4 cells. (**A**) Cell confluence in time (mean±SD; *n*=3). (**B**) Number of viable cells at 72 hours, relative to untreated (mean±SD; *n*=8). (**C-E**) RNA sequencing analysis. (**C**) Principal component analysis plot comparing vehicle and CM-272 samples. (**D-E**) Representative gene sets affected by CM-272, involved in (**D**) TP53 regulation of cell cycle progression, and of G1 phase arrest and apoptosis signaling, (**E**) Immune responses. Heatmaps list row-normalized gene expression of top-20 up/down-ranked genes. (**F**) Flow cytometry gating strategy of cell cycle distribution. (**G**) Percentage of MO4 cells in cell cycle phases at 72 hours (mean±SD; *n*=4). Asterisks represent sub-G1-phase. (**H**) p21 protein expression after 24 hours of treatment (mean±SD; *n*=3). (**I**) CFSE dilution upon 72 hours of polyclonal stimulation of $CD4^+$ or $CD8^+$ T cells (mean±SD; *n*=4). Vehicle and CM-272 conditions were compared using REML modeling and post-hoc Sidak multiple comparison test (A); Ordinary one-way Anova and post-hoc Dunnett's multiple comparison test (B,G,H,I). Asterisks indicate statistical significance: * p≤0.05; ** p≤0.01; *** p≤0.001; **** p≤0.0001.

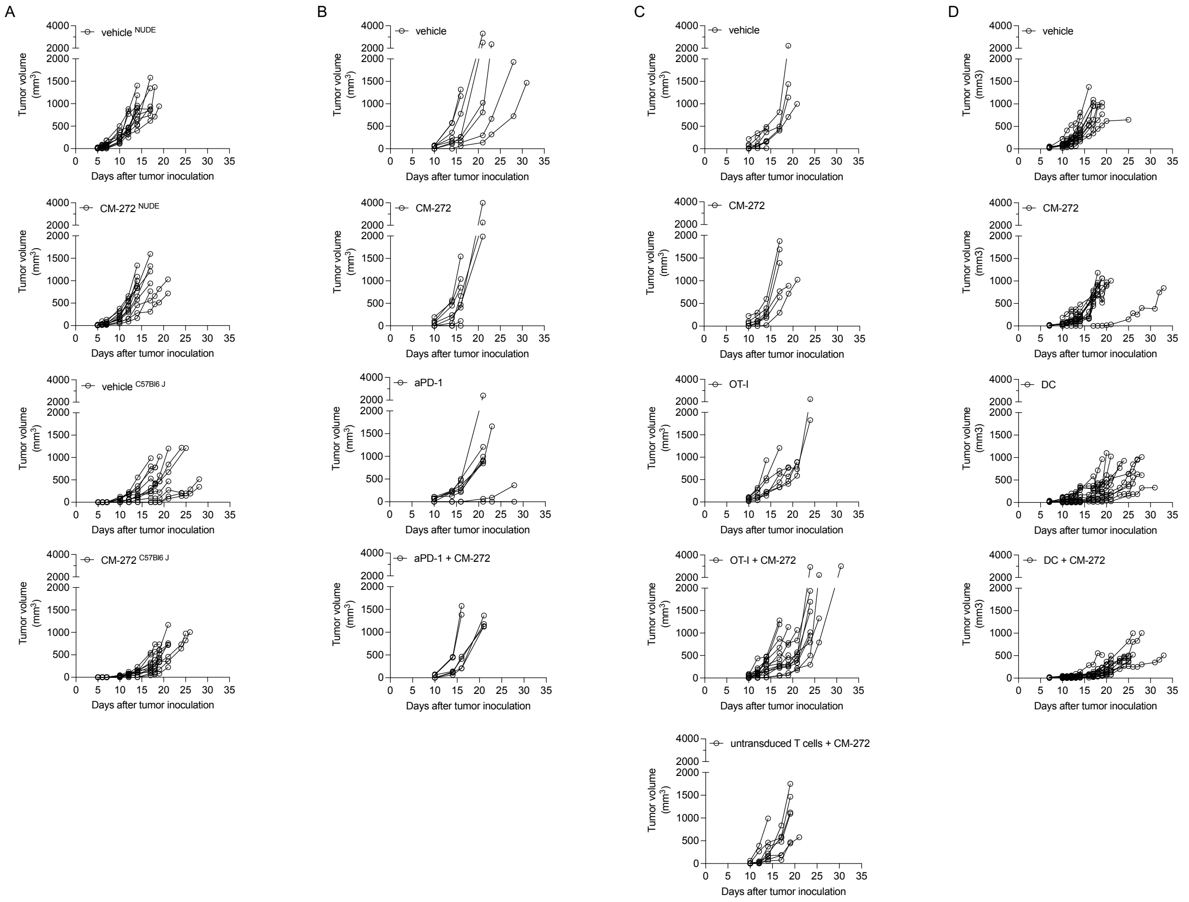

Figure S2 - Follow-up of in vivo MO4 tumor volume evolution in time. (**A-D**) Evolution of tumor volume in time of each individual mouse, presented per experimental group. (**A**) CM-272 in immune-deficient (NUDE) and immune-competent (C57Bl6 J) setting. (**B**) Single or combined CM-272 and PD-1 blockade (aPD-1) treatment. (**C**) Single or combined CM-272 and adoptive T-cell treatment. (**D**) Single or combined CM-272 and DC vaccination treatment.

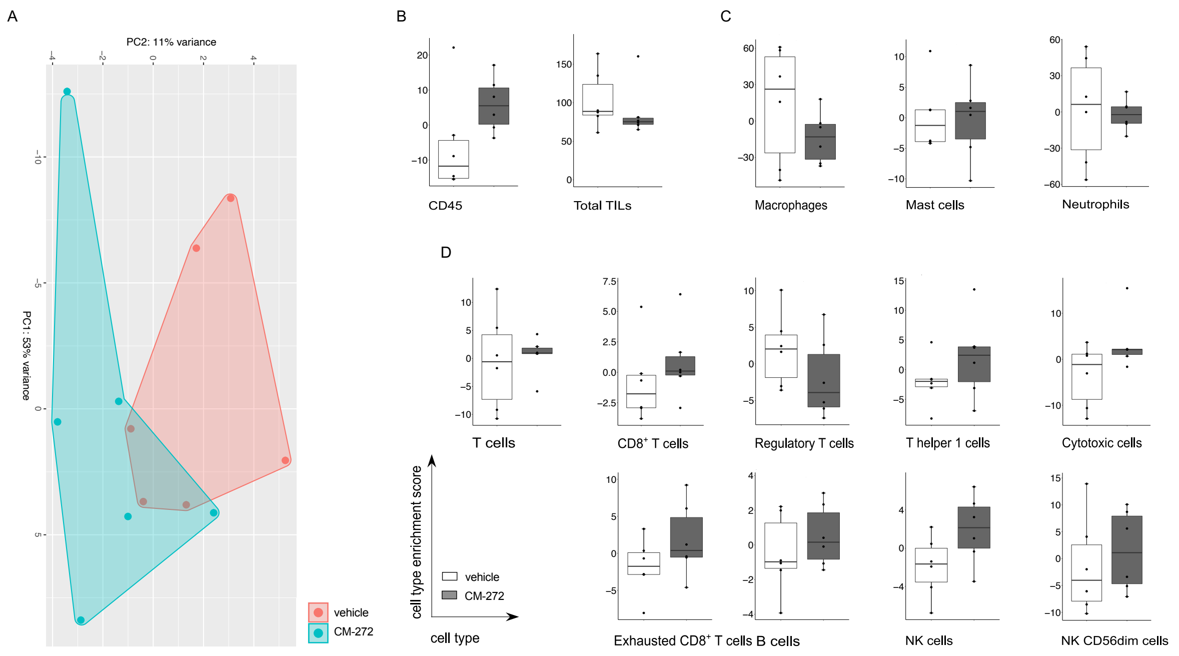

Figure S3 - Gene expression-based TIL-scoring on ex vivo tumor tissue. Tumors of MO4 tumor-bearing mice subjected to the CM-272 treatment regimen were resected at 706.9±193.8 mm^3 tumor volume (*n*=1, 12 m.p.c.) and subsequently subjected to multiplex analysis of 770 genes included in the Nanostring nCounter PanCancer Mouse Immune Profiling Panel. (**A**) Principal component analysis plot comparing vehicle and CM-272 samples. (**B-D**) Cell score of (**B**) total TILs and $CD45^+$ immune cells, (**C**) immune cells of the myeloid lineage, and (**D**) immune cells of the lymphoid lineage. Vehicle and CM-272 condition was compared using the Wilcoxon rank sum test (B-D).

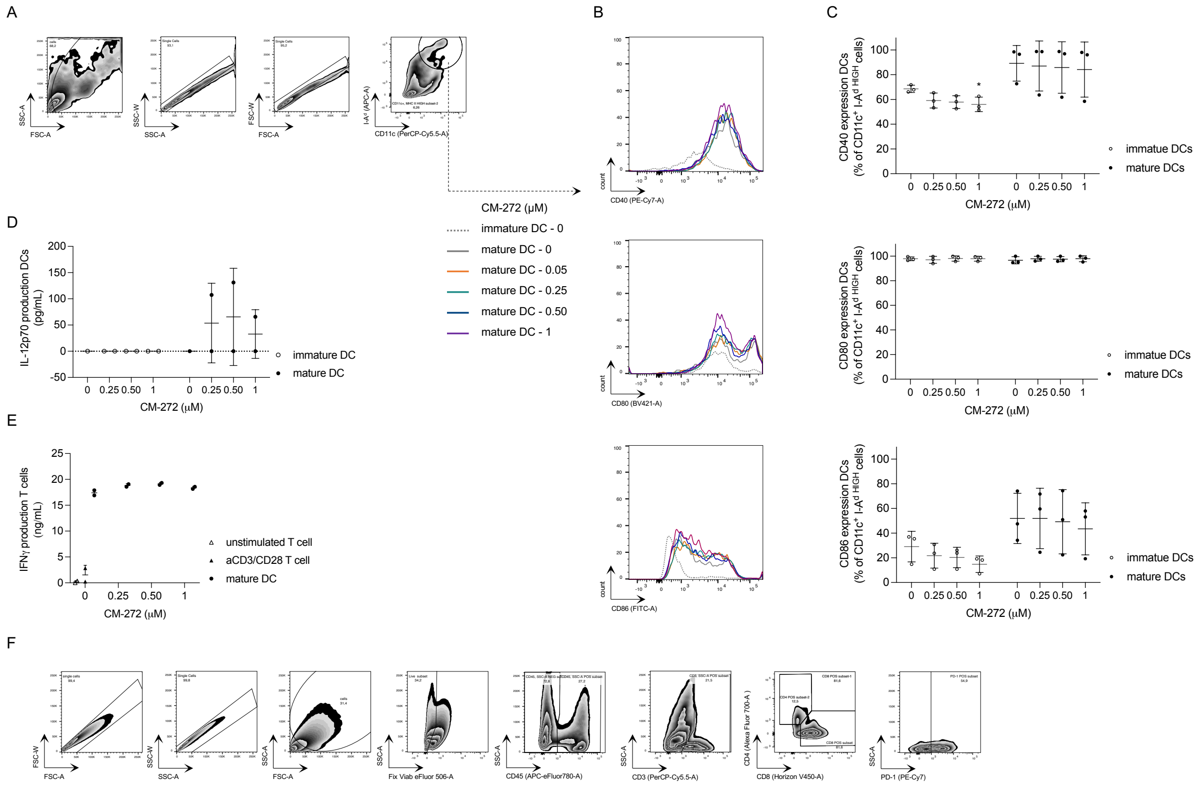

Figure S4 - Sensitivity of DCs to CM-272 exposure and flow cytometry gating strategy of ex vivo tumor T cell infiltrate. (**A-C**) Bone marrow-derived DCs were subjected to CM-272 for 24 hours before effect was determined on maturation marker (CD40, CD80, and CD86) expression of I-A$^{d\ HIGH}$/CD11c^{+} DCs. (**A**) Flow cytometry gating strategy. (**B-C**) Maturation marker expression (representative histograms [b]/ mean±SD; *n*=3 [c]). (**D**) IL-12p70 production by DCs (mean±SD; *n*=2). (**E**) IFN-γ production signifying T-cell activation by DCs (mean±SD; *n*=2). (**F**) Flow cytometry gating strategy of T-cell infiltration *in vivo*. Vehicle and CM-272 conditions were compared using ordinary one-way Anova and post-hoc Dunnett's multiple comparison test (C).
