# Supplementary material for: Inhibiting Histone and DNA Methylation Improves Cancer Vaccination in an Experimental Model of Melanoma
Source: Front Immunol. 2022 May 12;13:799636. doi: 10.3389/fimmu.2022.799636 (PMC9134079; doi:10.3389/fimmu.2022.799636)
Supplement: Supplementary file 2 [file DataSheet_2.pdf]

SUPPLEMENTARY TABLES

Table S1: RNA sequencing of CM-272-treated MO4 cells *in vitro* - normalized gene expression counts and log_2-fold changes (***page 1-58***); ***Table S2***: RNA sequencing of CM-272-treated MO4 cells *in vitro* - significantly changed gene sets (***page 59-68***); ***Table S3***: Multiplex gene expression analysis on *ex vivo* tumor tissue - significantly changed gene sets (***page 69***); ***Table S4***: Report on statistical significance pertaining to Figure 5C (***page 70***).

Table S1 – RNA sequencing of CM-272-treated MO4 cells *in vitro* - normalized gene expression counts and log_2-fold changes. [1]

Gene symbol	Log_2-fold change [2]	*q*-value	*p*-value
**Ccnb1-ps**	-4,1347375	0,0000000	0,00000000
**Ska1**	-4,0254682	0,0000000	0,00000000
**H2ac12**	-3,9147032	0,0000000	0,00000000
**Gm12856**	-3,7981566	0,0000000	0,00000000
**Pimreg**	-3,6647352	0,0000000	0,00000000
**Depdc1a**	-3,6566913	0,0000000	0,00000000
**Ccna2**	-3,6081603	0,0000000	0,00000000
**Cenpf**	-3,5921527	0,0000000	0,00000000
**Hmmr**	-3,540225	0,0000000	0,00000000
**Cenpe**	-3,5394196	0,0000000	0,00000000
**Plk1**	-3,499661	0,0000000	0,00000000
**Ccnb1**	-3,4988183	0,0000000	0,00000000
**Cdkn3**	-3,4883359	0,0000000	0,00000000
**Mki67**	-3,4763774	0,0000000	0,00000000
**Spag5**	-3,4486162	0,0000000	0,00000000
**Pif1**	-3,4407567	0,0000000	0,00000000
**Aurkb**	-3,4194024	0,0000000	0,00000000
**Nek2**	-3,4183896	0,0000000	0,00000000
**Ccnb2**	-3,4131431	0,0000000	0,00000000
**Cdk1**	-3,3936285	0,0000000	0,00000000
**Troap**	-3,3870243	0,0000000	0,00000000
**H2bu1-ps**	-3,3667004	0,0000000	0,00000000
**H3c8**	-3,3658868	0,0000000	0,00000000
**Kif20a**	-3,3619289	0,0000000	0,00000000
**Aspm**	-3,3548509	0,0000000	0,00000000
**H2ac4**	-3,3493837	0,0000000	0,00000000
**Bub1**	-3,3368604	0,0000000	0,00000000
**Ube2c**	-3,3337322	0,0000000	0,00000000
**Sapcd2**	-3,3300053	0,0000000	0,00000000
**Pbk**	-3,3245753	0,0000000	0,00000000
**Nuf2**	-3,3144117	0,0000000	0,00000000
**Cdc25c**	-3,3112061	0,0000000	0,00000000

**Ttk**	-3,3070152	0,0000000	0,00000000
**Cit**	-3,3021253	0,0000000	0,00000000
**Tacc3**	-3,2907224	0,0000000	0,00000000
**Knl1**	-3,2833459	0,0000000	0,00000000
**Cenpa**	-3,2776714	0,0000000	0,00000000
**Kif11**	-3,2767817	0,0000000	0,00000000
**Ckap2l**	-3,2726165	0,0000000	0,00000000
**Birc5**	-3,2711752	0,0000000	0,00000000
**Kif2c**	-3,2577494	0,0000000	0,00000000
**H3c3**	-3,254268	0,0000000	0,00000000
**Prr11**	-3,2269347	0,0000000	0,00000000
**Kif14**	-3,2218341	0,0000000	0,00000000
**Dlgap5**	-3,2168215	0,0000000	0,00000000
**Kif15**	-3,2127121	0,0000000	0,00000000
**H2bc11**	-3,1915446	0,0000000	0,00000000
**Cep55**	-3,1864117	0,0000000	0,00000000
**Sgo1**	-3,1823859	0,0000000	0,00000000
**Racgap1**	-3,1661056	0,0000000	0,00000000
**Cdca3**	-3,1598981	0,0000000	0,00000000
**Ndc80**	-3,1504508	0,0000000	0,00000000
**H2bc12**	-3,1458645	0,0000000	0,00000000
**Iqgap3**	-3,1257731	0,0000000	0,00000000
**Knstrn**	-3,1233334	0,0000000	0,00000000
**E2f8**	-3,1160295	0,0000000	0,00000000
**Tpx2**	-3,0982327	0,0000000	0,00000000
**Cdca8**	-3,0946976	0,0000000	0,00000000
**Shcbp1**	-3,0779693	0,0000000	0,00000000
**Cip2a**	-3,0700741	0,0000000	0,00000000
**Spc24**	-3,0692056	0,0000000	0,00000000
**Clspn**	-3,0681375	0,0000000	0,00000000
**Top2a**	-3,0672933	0,0000000	0,00000000
**Mybl2**	-3,0387531	0,0000000	0,00000000
**Phf19**	-3,0371398	0,0000000	0,00000000
**Espl1**	-3,0347456	0,0000000	0,00000000
**Hmgb2**	-3,0207861	0,0000000	0,00000000
**Cdca2**	-3,0167948	0,0000000	0,00000000
**Kif4**	-3,0149558	0,0000000	0,00000000
**Arhgef39**	-3,0135798	0,0000000	0,00000000
**Kif18b**	-2,9927619	0,0000000	0,00000000
**Gm13237**	-2,9833873	0,0000000	0,00000000
**Bub1b**	-2,982978	0,0000000	0,00000000
**Kif23**	-2,9739727	0,0000000	0,00000000

**Neil3**	-2,9665909	0,0000000	0,00000000
**H2ac8**	-2,9551475	0,0000000	0,00000000
**Cdca5**	-2,9471841	0,0000000	0,00000000
**H2ac10**	-2,9435189	0,0000000	0,00000000
**Arhgap19**	-2,9418074	0,0000000	0,00000000
**Kif20b**	-2,9403812	0,0000000	0,00000000
**Sgo2a**	-2,9359783	0,0000000	0,00000000
**H2bc3**	-2,9356635	0,0000000	0,00000000
**Gmnn**	-2,9306823	0,0000000	0,00000000
**Polq**	-2,9302649	0,0000000	0,00000000
**Melk**	-2,9160451	0,0000000	0,00000000
**Chaf1b**	-2,9131269	0,0000000	0,00000000
**Cdc20**	-2,9104946	0,0000000	0,00000000
**Dna2**	-2,9067503	0,0000000	0,00000000
**H3c1**	-2,9036269	0,0000000	0,00000000
**Kif22**	-2,8979858	0,0000000	0,00000000
**Fbxo5**	-2,8979286	0,0000000	0,00000000
**Ncapd2**	-2,8955089	0,0000000	0,00000000
**Mcm5**	-2,8934462	0,0000000	0,00000000
**Aurka**	-2,8899949	0,0000000	0,00000000
**Mns1**	-2,8767008	0,0000000	0,00000000
**Hrob**	-2,8636668	0,0000000	0,00000000
**Uhrf1**	-2,862257	0,0000000	0,00000000
**Ncapg**	-2,8537155	0,0000000	0,00000000
**H2ax**	-2,8493259	0,0000000	0,00000000
**Cenpi**	-2,8439729	0,0000000	0,00000000
**Tk1**	-2,8423123	0,0000000	0,00000000
**Prc1**	-2,8381259	0,0000000	0,00000000
**Parpbp**	-2,8353337	0,0000000	0,00000000
**H4f16**	-2,8326882	0,0000000	0,00000000
**Nup210**	-2,8228022	0,0000000	0,00000000
**H2ac11**	-2,8145992	0,0000000	0,00000000
**Ticrr**	-2,8018397	0,0000000	0,00000000
**Gm15428**	-2,7952902	0,0000000	0,00000000
**Ccnf**	-2,7890627	0,0000000	0,00000000
**Gm6091**	-2,7858897	0,0000000	0,00000000
**Kntc1**	-2,7825599	0,0000000	0,00000000
**Gm5853**	-2,775122	0,0000000	0,00000000
**Gm5648**	-2,7714933	0,0000000	0,00000000
**Ercc6l**	-2,7678747	0,0000000	0,00000000
**Pclaf**	-2,7641846	0,0000000	0,00000000
**Mtfr2**	-2,7429425	0,0000000	0,00000000

**Lig1**	-2,7212199	0,0000000	0,00000000
**H2bc6**	-2,7197708	0,0000000	0,00000000
**Nsl1**	-2,7190627	0,0000000	0,00000000
**Gm13160**	-2,7169813	0,0000001	0,00000001
**Incenp**	-2,7152748	0,0000000	0,00000000
**Atad2**	-2,7029774	0,0000000	0,00000000
**Mis18bp1**	-2,6921126	0,0000000	0,00000000
**Dscc1**	-2,6868389	0,0000000	0,00000000
**H4c14**	-2,6784439	0,0000000	0,00000000
**Rmi2**	-2,6567392	0,0000000	0,00000000
**Ect2**	-2,6506809	0,0000000	0,00000000
**H4c6**	-2,6493017	0,0000000	0,00000000
**Mcm10**	-2,6420664	0,0000000	0,00000000
**Mxd3**	-2,6408284	0,0000000	0,00000000
**H3c7**	-2,6407454	0,0000000	0,00000000
**Kif18a**	-2,6385523	0,0000000	0,00000000
**Aunip**	-2,6368671	0,0000000	0,00000000
**H2bc8**	-2,6234364	0,0000000	0,00000000
**Fanci**	-2,6212722	0,0000000	0,00000000
**Fancd2**	-2,6196934	0,0000000	0,00000000
**Mcm2**	-2,6129593	0,0000000	0,00000000
**Ncapg2**	-2,6069562	0,0000000	0,00000000
**Mcm3**	-2,6038143	0,0000000	0,00000000
**Gen1**	-2,599698	0,0000000	0,00000000
**E2f2**	-2,5959678	0,0000000	0,00000000
**Exo1**	-2,5510484	0,0000000	0,00000000
**Bard1**	-2,5468316	0,0000000	0,00000000
**Rad51ap1**	-2,5468007	0,0000000	0,00000000
**H1f3**	-2,5407484	0,0000000	0,00000000
**H4c1**	-2,5403381	0,0000000	0,00000000
**Traip**	-2,5235152	0,0000000	0,00000000
**Cdc6**	-2,4924737	0,0000000	0,00000000
**H3c6**	-2,4915301	0,0000000	0,00000000
**Mad2l1**	-2,4893762	0,0000000	0,00000000
**Cdc45**	-2,4862271	0,0000000	0,00000000
**Gins1**	-2,4764353	0,0000000	0,00000000
**N4bp3**	-2,4695288	0,0000000	0,00000000
**H2bc4**	-2,4633956	0,0000000	0,00000000
**Ccdc34**	-2,4619313	0,0000000	0,00000000
**Wdr62**	-2,4443986	0,0000000	0,00000000
**Pmf1**	-2,4339944	0,0000000	0,00000000
**Cdc25b**	-2,4276333	0,0000000	0,00000000

**Pold1**	-2,4176296	0,0000000	0,00000000
**Cdt1**	-2,4171866	0,0000000	0,00000000
**Depdc1b**	-2,4153508	0,0000003	0,00000004
**Rrm2**	-2,4150193	0,0000000	0,00000000
**Rad51**	-2,4085386	0,0000000	0,00000000
**Eme1**	-2,406928	0,0000000	0,00000000
**Esco2**	-2,4043727	0,0000000	0,00000000
**Gjb2**	-2,4042934	0,0000000	0,00000000
**Lmnb1**	-2,3996858	0,0000000	0,00000000
**H4c2**	-2,3988428	0,0000000	0,00000000
**Pkmyt1**	-2,3984809	0,0000000	0,00000000
**Foxm1**	-2,3867811	0,0000000	0,00000000
**Trip13**	-2,3865299	0,0000000	0,00000000
**Cenpu**	-2,3849786	0,0000000	0,00000000
**Nusap1**	-2,3830458	0,0000000	0,00000000
**Cntfr**	-2,3751741	0,0000000	0,00000000
**Rbl1**	-2,3745396	0,0000000	0,00000000
**Cenpw**	-2,3696453	0,0000000	0,00000000
**Plk4**	-2,3612534	0,0000000	0,00000000
**Chek1**	-2,3583141	0,0000000	0,00000000
**Chaf1a**	-2,3570417	0,0000000	0,00000000
**Fanca**	-2,355321	0,0000000	0,00000000
**Prim1**	-2,3526523	0,0000000	0,00000000
**Cenph**	-2,3526083	0,0000000	0,00000000
**Aire**	-2,3467895	0,0000225	0,00000424
**H4c8**	-2,3399366	0,0000000	0,00000000
**H1f6**	-2,3288339	0,0000000	0,00000000
**Ankle1**	-2,3265039	0,0000000	0,00000000
**H1f4**	-2,3222333	0,0000000	0,00000000
**Arhgap11a**	-2,3075014	0,0000000	0,00000000
**Gpsm2**	-2,3058961	0,0000000	0,00000000
**Ung**	-2,292136	0,0000000	0,00000000
**Pole**	-2,2862413	0,0000000	0,00000000
**Gm12387**	-2,2703188	0,0001610	0,00003492
**H2ac6**	-2,2679386	0,0000000	0,00000000
**Mcm7**	-2,2568544	0,0000000	0,00000000
**Mlf1**	-2,2476333	0,0000000	0,00000000
**Cenpm**	-2,2468687	0,0000000	0,00000000
**Fndc3c1**	-2,2465597	0,0000000	0,00000000
**H2ac20**	-2,2434024	0,0000000	0,00000000
**Wdhd1**	-2,2353798	0,0000000	0,00000000
**Dbf4**	-2,2308662	0,0000000	0,00000000

**Cenps**	-2,2131403	0,0000000	0,00000000
**Tyms**	-2,207161	0,0000000	0,00000000
**Hmx2**	-2,192737	0,0000000	0,00000000
**Mastl**	-2,1882585	0,0000000	0,00000000
**Cdc25a**	-2,1858425	0,0000000	0,00000000
**Impa2**	-2,1824926	0,0000000	0,00000000
**Cenpq**	-2,1798743	0,0000000	0,00000000
**Ska3**	-2,1780578	0,0000000	0,00000000
**Atad5**	-2,1729647	0,0000000	0,00000000
**Lmo1**	-2,1706644	0,0000000	0,00000000
**2700099C18Rik**	-2,1695322	0,0000000	0,00000000
**Chtf18**	-2,1672737	0,0000000	0,00000000
**Hmgb1-ps3**	-2,1623297	0,0000149	0,00000273
**H4c3**	-2,1619331	0,0000000	0,00000000
**Iqgap2**	-2,1586557	0,0000000	0,00000000
**Mcm4**	-2,1574914	0,0000000	0,00000000
**Vash1**	-2,1479079	0,0000000	0,00000000
**Efcab11**	-2,1427908	0,0000000	0,00000000
**H4c4**	-2,1388975	0,0000000	0,00000000
**Enpp2**	-2,1350249	0,0000000	0,00000000
**Smc2**	-2,1288572	0,0000000	0,00000000
**Cep152**	-2,1266937	0,0000000	0,00000000
**2810429I04Rik**	-2,125589	0,0000000	0,00000000
**Cep89**	-2,1228246	0,0000000	0,00000000
**Gm44521**	-2,1222818	0,0002261	0,00005034
**Lrr1**	-2,1194085	0,0000000	0,00000000
**Rrm1**	-2,1115537	0,0000000	0,00000000
**Cdk2**	-2,1114148	0,0000000	0,00000000
**Ccne2**	-2,1062309	0,0000000	0,00000000
**Ccsap**	-2,0969306	0,0000000	0,00000000
**Ccdc85c**	-2,0768439	0,0000000	0,00000000
**Stil**	-2,0721507	0,0000000	0,00000000
**Mgam2-ps**	-2,0702346	0,0000001	0,00000001
**Timeless**	-2,0641858	0,0000000	0,00000000
**Dut**	-2,05539	0,0000000	0,00000000
**E2f7**	-2,053647	0,0000000	0,00000000
**Gm20667**	-2,0512635	0,0000002	0,00000002
**Orc1**	-2,047915	0,0000000	0,00000000
**Arhgap33**	-2,0453785	0,0005470	0,00013191
**Asf1b**	-2,0428776	0,0000000	0,00000000
**Rad54l**	-2,0425766	0,0000000	0,00000000
**Spdl1**	-2,0403423	0,0000000	0,00000000

**4930558J18Rik**	-2,0384679	0,0000061	0,00000107
**Ube2t**	-2,0229467	0,0000000	0,00000000
**E2f1**	-2,0226574	0,0000000	0,00000000
**Dtl**	-2,0207382	0,0000000	0,00000000
**Kifc1**	-2,0196331	0,0000000	0,00000000
**Spc25**	-2,0165306	0,0000000	0,00000000
**Mcm6**	-2,0165224	0,0000000	0,00000000
**Pitpnm3**	-2,0129947	0,0003126	0,00007185
**Recql4**	-2,0127097	0,0000000	0,00000000
**Smtnl2**	-2,0123602	0,0000000	0,00000000
**Aqp1**	-2,0115104	0,0000001	0,00000002
**Gm15452**	-2,0111721	0,0000000	0,00000000
**Lpar5**	-2,0084814	0,0000000	0,00000000
**Shmt1**	-2,0078264	0,0000000	0,00000000
**Dnmt1**	-1,9916607	0,0000000	0,00000000
**Xkr5**	-1,9782918	0,0000000	0,00000000
**Nckap5l**	-1,9743013	0,0000000	0,00000000
**Ccne1**	-1,9733045	0,0000000	0,00000000
**Batf3**	-1,9722207	0,0000000	0,00000000
**Gm10282**	-1,9720448	0,0000000	0,00000000
**Haspin**	-1,9627518	0,0000000	0,00000000
**Megf6**	-1,9592576	0,0000000	0,00000000
**Ddx11**	-1,957994	0,0000000	0,00000000
**Mpp6**	-1,9560401	0,0000000	0,00000000
**Pla2g4c**	-1,9492965	0,0007419	0,00018376
**Gm12261**	-1,9445674	0,0000000	0,00000000
**Sgcg**	-1,9440108	0,0000000	0,00000000
**Rpa2**	-1,9412146	0,0000000	0,00000000
**Rragd**	-1,9312524	0,0000000	0,00000000
**Ncaph**	-1,9283816	0,0000000	0,00000000
**Nasp**	-1,9280666	0,0000000	0,00000000
**Lrfn1**	-1,9237936	0,0000000	0,00000000
**Smc4**	-1,9230941	0,0000000	0,00000000
**Diaph3**	-1,9201467	0,0000000	0,00000000
**Hmgn2**	-1,9186663	0,0000000	0,00000000
**Cep128**	-1,9124486	0,0000000	0,00000000
**Zwilch**	-1,9100512	0,0000000	0,00000000
**Pole2**	-1,9085832	0,0000000	0,00000000
**H3c2**	-1,9052792	0,0000000	0,00000000
**Neurl1b**	-1,901566	0,0000227	0,00000429
**Cenpk**	-1,9005434	0,0000000	0,00000000
**Brip1**	-1,8992739	0,0000000	0,00000000

**2010110K18Rik**	-1,8961877	0,0012089	0,00031104
**Rad54b**	-1,8850622	0,0000000	0,00000000
**Gins2**	-1,8812065	0,0000000	0,00000000
**Wdr76**	-1,8758767	0,0000000	0,00000000
**Cenpn**	-1,8679685	0,0000000	0,00000000
**Gm3756**	-1,8646015	0,0000000	0,00000000
**Gm15387**	-1,8614012	0,0000003	0,00000004
**Fignl1**	-1,8612409	0,0000000	0,00000000
**Ctdspl**	-1,8569196	0,0000000	0,00000000
**Bora**	-1,8561479	0,0000000	0,00000000
**Cks2**	-1,8530874	0,0000000	0,00000000
**Hlf**	-1,8511282	0,0000000	0,00000000
**Fam83d**	-1,8509055	0,0000024	0,00000040
**Glt1d1**	-1,8479909	0,0000000	0,00000000
**Rapsn**	-1,8432006	0,0000000	0,00000000
**Pola2**	-1,8391981	0,0000000	0,00000000
**Gm6750**	-1,8370299	0,0002925	0,00006679
**Mis18a**	-1,8357435	0,0000000	0,00000000
**Rfc5**	-1,8330447	0,0000000	0,00000000
**Fam110a**	-1,8327323	0,0000000	0,00000000
**Poc1a**	-1,8317179	0,0000000	0,00000000
**Gm37599**	-1,8270008	0,0000409	0,00000805
**Gm9430**	-1,8103811	0,0003453	0,00007990
**Arhgap32**	-1,8078322	0,0000000	0,00000000
**Hpse**	-1,8036443	0,0000000	0,00000000
**Dmxl2**	-1,7955166	0,0000000	0,00000000
**Blm**	-1,7946543	0,0000000	0,00000000
**Vrk2**	-1,7904219	0,0000000	0,00000000
**Myo7a**	-1,784834	0,0000000	0,00000000
**Tcf19**	-1,7821047	0,0000000	0,00000000
**Map2k6**	-1,775362	0,0000000	0,00000000
**Tyro3**	-1,7752341	0,0000000	0,00000000
**Prickle1**	-1,774294	0,0000000	0,00000000
**2010008C14Rik**	-1,7727535	0,0000000	0,00000000
**Nmral1**	-1,7724385	0,0000000	0,00000000
**Klhl23**	-1,7692622	0,0000000	0,00000000
**Pmel**	-1,7659252	0,0000000	0,00000000
**Rapgef3os2**	-1,76436	0,0000006	0,00000009
**Dnmt3b**	-1,7641819	0,0000000	0,00000000
**Lockd**	-1,7572736	0,0000000	0,00000000
**Ptprs**	-1,7546021	0,0000000	0,00000000
**Hmgn2-ps1**	-1,7545762	0,0000437	0,00000865

**Fancb**	-1,7535402	0,0000000	0,00000000
**Bace2**	-1,75283	0,0000000	0,00000000
**Hvcn1**	-1,7502179	0,0000000	0,00000000
**Tuba1b**	-1,7492275	0,0000000	0,00000000
**Gm10357**	-1,7479874	0,0003568	0,00008290
**Gm21596**	-1,7451742	0,0000001	0,00000002
**Gm11349**	-1,7429325	0,0000623	0,00001268
**Xylb**	-1,7415624	0,0000000	0,00000000
**Gm43096**	-1,7361797	0,0000000	0,00000000
**Cep72**	-1,7322786	0,0000000	0,00000000
**Selenoh**	-1,7315521	0,0000000	0,00000000
**Gm37510**	-1,7280946	0,0000012	0,00000020
**Myb**	-1,7262957	0,0007340	0,00018156
**Rad51b**	-1,7259414	0,0001479	0,00003188
**Itpk1**	-1,7240997	0,0000000	0,00000000
**Tenm4**	-1,7220576	0,0000000	0,00000000
**Mboat1**	-1,7207371	0,0000000	0,00000000
**Kifc5b**	-1,7194629	0,0000000	0,00000000
**Cbx3-ps7**	-1,7129792	0,0000000	0,00000000
**Enkd1**	-1,7116408	0,0000000	0,00000000
**Ezh2**	-1,7096555	0,0000000	0,00000000
**Alms1**	-1,7064411	0,0000000	0,00000000
**Fndc4**	-1,7044673	0,0000000	0,00000000
**Haus4**	-1,7022229	0,0000000	0,00000000
**Echdc2**	-1,7006805	0,0000000	0,00000000
**Rfc4**	-1,6950347	0,0000000	0,00000000
**Mnd1**	-1,6939715	0,0000044	0,00000076
**Gm36401**	-1,6882516	0,0002056	0,00004544
**Adamts20**	-1,6864591	0,0000000	0,00000000
**H1f1**	-1,6862726	0,0000008	0,00000012
**Smo**	-1,6862544	0,0000000	0,00000000
**Gm43029**	-1,6842704	0,0000002	0,00000003
**Mnd1-ps**	-1,6779191	0,0004737	0,00011281
**Nsmce4a**	-1,6772262	0,0000000	0,00000000
**Hmgb1**	-1,6751614	0,0000000	0,00000000
**Lbr**	-1,6710142	0,0000000	0,00000000
**Nt5dc2**	-1,6681306	0,0000000	0,00000000
**Anln**	-1,6664051	0,0000000	0,00000000
**Parp1**	-1,6661662	0,0000000	0,00000000
**Zfp469**	-1,6632062	0,0000000	0,00000000
**Topbp1**	-1,6593869	0,0000000	0,00000000
**Gm5620**	-1,6570692	0,0000000	0,00000000

**Chek2**	-1,6566106	0,0000000	0,00000000
**Gm16436**	-1,6523979	0,0010344	0,00026246
**Hirip3**	-1,6469574	0,0000000	0,00000000
**Tnfaip8l1**	-1,6440393	0,0000008	0,00000013
**Tmem51**	-1,6389469	0,0000000	0,00000000
**Palb2**	-1,6389381	0,0000000	0,00000000
**Hmgb3**	-1,6351362	0,0000000	0,00000000
**Brca1**	-1,6320397	0,0000000	0,00000000
**Dcbld1**	-1,6314189	0,0000000	0,00000000
**Cdca7**	-1,6310517	0,0000000	0,00000000
**Galnt12**	-1,6293282	0,0000000	0,00000000
**5031439G07Rik**	-1,6281196	0,0000000	0,00000000
**Cdc7**	-1,6263398	0,0000000	0,00000000
**Tfap4**	-1,6152388	0,0000000	0,00000000
**Cers1**	-1,6149443	0,0000000	0,00000000
**Hat1**	-1,6144047	0,0000000	0,00000000
**Wee1**	-1,6127475	0,0000000	0,00000000
**Macroh2a1**	-1,6091532	0,0000000	0,00000000
**Gm6104**	-1,6080714	0,0000000	0,00000000
**Kif24**	-1,608055	0,0000000	0,00000000
**Tmcc2**	-1,6005149	0,0000000	0,00000000
**Trim59**	-1,5969864	0,0000000	0,00000000
**Pygo1**	-1,5937739	0,0000000	0,00000000
**Slc16a3**	-1,5936605	0,0002853	0,00006508
**Dlk2**	-1,5919667	0,0011430	0,00029216
**Pola1**	-1,5913261	0,0000000	0,00000000
**Mms22l**	-1,5901189	0,0000000	0,00000000
**Gm28229**	-1,5898851	0,0000000	0,00000000
**Hells**	-1,589837	0,0000000	0,00000000
**Plch1**	-1,5886979	0,0000000	0,00000000
**Poc1b**	-1,5871307	0,0000000	0,00000000
**Gm17383**	-1,5858665	0,0000000	0,00000000
**Gm12870**	-1,5840846	0,0005115	0,00012254
**Hmcn1**	-1,5815843	0,0000000	0,00000000
**Usp1**	-1,5811451	0,0000000	0,00000000
**Cpne4**	-1,5784176	0,0000000	0,00000000
**Exosc8**	-1,5690392	0,0000000	0,00000000
**Ckap5**	-1,568641	0,0000000	0,00000000
**Dsn1**	-1,563507	0,0000000	0,00000000
**Mdm1**	-1,5611608	0,0000000	0,00000000
**H2ac21**	-1,557662	0,0000300	0,00000579
**Tnk2**	-1,5540373	0,0000000	0,00000000

**Degs2**	-1,5536161	0,0009131	0,00022919
**Skp2**	-1,5533928	0,0000000	0,00000000
**Gm11223**	-1,5533413	0,0000000	0,00000000
**Tipin**	-1,5521749	0,0000000	0,00000000
**Pcna-ps2**	-1,5448787	0,0000000	0,00000000
**BC055324**	-1,5446362	0,0000000	0,00000000
**Cgas**	-1,5419054	0,0000000	0,00000000
**Hip1r**	-1,5415877	0,0000000	0,00000000
**Mapk11**	-1,5380426	0,0000000	0,00000000
**Stmn1**	-1,5377802	0,0000000	0,00000000
**Tspan10**	-1,536284	0,0000000	0,00000000
**Pcna**	-1,5358703	0,0000000	0,00000000
**Ppid**	-1,535447	0,0000000	0,00000000
**Oip5**	-1,5318855	0,0000000	0,00000000
**Clmn**	-1,5307064	0,0000000	0,00000000
**Cbx2**	-1,5264321	0,0000000	0,00000000
**Mtfp1**	-1,5254271	0,0000000	0,00000000
**Sass6**	-1,5246978	0,0000000	0,00000000
**Kpna2**	-1,5211598	0,0000000	0,00000000
**Vrk1**	-1,5202885	0,0000000	0,00000000
**Gm10184**	-1,5165647	0,0000000	0,00000000
**Cenpl**	-1,511139	0,0000000	0,00000000
**Lmnb2**	-1,5076918	0,0000000	0,00000000
**Gm7931**	-1,5072555	0,0001944	0,00004276
**Net1**	-1,5046169	0,0000000	0,00000000
**Gm13577**	-1,5005041	0,0000029	0,00000049
**Vegfb**	-1,4985282	0,0000000	0,00000000
**Mt2**	-1,4970963	0,0000018	0,00000029
**Hsf4**	-1,4959603	0,0000000	0,00000000
**Cks1b**	-1,4936192	0,0000000	0,00000000
**Dek**	-1,4903492	0,0000000	0,00000000
**Pold2**	-1,485098	0,0000000	0,00000000
**Mtbp**	-1,4840852	0,0000000	0,00000000
**Fxn**	-1,4813692	0,0000000	0,00000000
**Mknk2**	-1,4812741	0,0000000	0,00000000
**Tamm41**	-1,4804289	0,0000000	0,00000000
**Egln1**	-1,4776218	0,0000000	0,00000000
**Paqr6**	-1,4735791	0,0000001	0,00000002
**Plekhg3**	-1,4653316	0,0000000	0,00000000
**Abca12**	-1,4616558	0,0000000	0,00000000
**Rcc1**	-1,4611474	0,0000000	0,00000000
**Ttf2**	-1,4607659	0,0000000	0,00000000

**Tubg1**	-1,4531276	0,0000000	0,00000000
**Cibar1**	-1,4528908	0,0000000	0,00000000
**Gpr157**	-1,4479397	0,0000000	0,00000000
**Nsd2**	-1,4455873	0,0000000	0,00000000
**Bicc1**	-1,4403303	0,0000000	0,00000000
**Hjurp**	-1,4399345	0,0000000	0,00000000
**Tubb5**	-1,4395642	0,0000000	0,00000000
**Sdf2l1**	-1,4395565	0,0000000	0,00000000
**Fbxo48**	-1,4380346	0,0011562	0,00029591
**Ntrk3**	-1,4361224	0,0012000	0,00030833
**Gm10619**	-1,4354177	0,0012914	0,00033375
**Rcor2**	-1,4348554	0,0000000	0,00000000
**Scn1a**	-1,4338622	0,0000000	0,00000000
**Cenpv**	-1,4319954	0,0000000	0,00000000
**Ccdc69**	-1,4281036	0,0000001	0,00000002
**Cep192**	-1,427951	0,0000000	0,00000000
**Dhfr**	-1,4261833	0,0000000	0,00000000
**Mreg**	-1,4257182	0,0000000	0,00000000
**Suv39h1**	-1,4244619	0,0000000	0,00000000
**Cerox1**	-1,4244045	0,0015008	0,00039233
**Gm14276**	-1,4240307	0,0000001	0,00000001
**Gm12919**	-1,4207134	0,0004396	0,00010399
**Psmc3ip**	-1,4159314	0,0000000	0,00000000
**Cep135**	-1,4134982	0,0000000	0,00000000
**Dck**	-1,4107803	0,0000000	0,00000000
**Rnaseh2b**	-1,4064145	0,0000000	0,00000000
**Fbln1**	-1,4043517	0,0000000	0,00000000
**Paxip1**	-1,4022158	0,0000000	0,00000000
**Ccdc57**	-1,4000419	0,0000000	0,00000000
**Terf1**	-1,3990442	0,0000000	0,00000000
**Psen2**	-1,3988194	0,0000000	0,00000000
**Igsf8**	-1,3986751	0,0000000	0,00000000
**Pdk1**	-1,3979763	0,0000154	0,00000282
**Pold3**	-1,3976708	0,0000000	0,00000000
**Slfn9**	-1,3952052	0,0000000	0,00000000
**Tmem109**	-1,3912244	0,0000000	0,00000000
**Rttn**	-1,3904136	0,0000000	0,00000000
**Afap1l1**	-1,3894963	0,0000000	0,00000000
**Bckdhb**	-1,3832128	0,0000000	0,00000000
**Tjp2**	-1,3827337	0,0000000	0,00000000
**Gm14150**	-1,3817134	0,0000000	0,00000000
**D630045J12Rik**	-1,3798563	0,0000000	0,00000000

**Bhlhe40**	-1,3757357	0,0000000	0,00000000
**Dnph1**	-1,37455	0,0000000	0,00000000
**Plekhg5**	-1,373761	0,0000000	0,00000000
**Cntrob**	-1,3733027	0,0000000	0,00000000
**Cbx3-ps6**	-1,37235	0,0000000	0,00000000
**Gas2l3**	-1,369141	0,0000000	0,00000000
**Gm10074**	-1,3670756	0,0000000	0,00000000
**Slc29a1**	-1,3624428	0,0000000	0,00000000
**Tmem268**	-1,3603658	0,0000000	0,00000000
**Cntln**	-1,356192	0,0002693	0,00006106
**Tube1**	-1,3496785	0,0000002	0,00000003
**Cars2**	-1,3485028	0,0000000	0,00000000
**Ncapd3**	-1,343735	0,0000000	0,00000000
**Rbmx**	-1,3414642	0,0000000	0,00000000
**Metrn**	-1,3399493	0,0000020	0,00000032
**2810410L24Rik**	-1,3399056	0,0000003	0,00000005
**Bcl7a**	-1,3395675	0,0000000	0,00000000
**Mcph1**	-1,3370937	0,0000000	0,00000000
**Haus1**	-1,3350358	0,0000000	0,00000000
**Gpr143**	-1,3345582	0,0000000	0,00000000
**Wdr90**	-1,3343104	0,0000000	0,00000000
**Prkcz**	-1,3342679	0,0000000	0,00000000
**Brca2**	-1,3289344	0,0000000	0,00000000
**Aff3**	-1,3270444	0,0000000	0,00000000
**Cdca7l**	-1,3269764	0,0000000	0,00000000
**Pkp4**	-1,3238606	0,0000000	0,00000000
**Fam184b**	-1,322853	0,0000000	0,00000000
**Arhgef37**	-1,3224172	0,0002161	0,00004797
**Fen1**	-1,3220808	0,0000149	0,00000273
**Mt1**	-1,3206777	0,0000017	0,00000028
**Lin9**	-1,3176814	0,0000000	0,00000000
**Kdm2b**	-1,3173155	0,0000000	0,00000000
**Thra**	-1,3169475	0,0000000	0,00000000
**Eogt**	-1,3168114	0,0000000	0,00000000
**Gm8146**	-1,3148394	0,0003076	0,00007059
**Tal1**	-1,3139247	0,0000060	0,00000106
**Tnfsf9**	-1,312176	0,0000000	0,00000000
**4930579G24Rik**	-1,3093117	0,0000000	0,00000000
**Msh6**	-1,3063047	0,0000000	0,00000000
**Ifrd2**	-1,302833	0,0000000	0,00000000
**Pwwp3a**	-1,2972639	0,0000000	0,00000000
**Kmt5a**	-1,2972367	0,0000000	0,00000000

**Gm11585**	-1,2956657	0,0000000	0,00000000
**Pde9a**	-1,2953117	0,0000001	0,00000002
**Gm42549**	-1,2950817	0,0018740	0,00049997
**Parvb**	-1,2907131	0,0000000	0,00000000
**Uchl5**	-1,2906329	0,0000000	0,00000000
**Hunk**	-1,2855301	0,0000000	0,00000000
**Thrap3**	-1,2846699	0,0000000	0,00000000
**Odf2**	-1,2834141	0,0000000	0,00000000
**Tedc1**	-1,2833681	0,0000000	0,00000000
**Ybx3**	-1,2810631	0,0000000	0,00000000
**Alg13**	-1,2798782	0,0000000	0,00000000
**Gm5841**	-1,2797041	0,0000017	0,00000027
**Cyp39a1**	-1,2792588	0,0000000	0,00000000
**A930004J17Rik**	-1,2789907	0,0001261	0,00002692
**Paqr4**	-1,2786124	0,0000000	0,00000000
**Anp32b**	-1,2748199	0,0000000	0,00000000
**Vars**	-1,2744722	0,0000000	0,00000000
**Eps8**	-1,2732668	0,0000000	0,00000000
**Slf1**	-1,2715996	0,0000000	0,00000000
**Prdm16**	-1,2658516	0,0000000	0,00000000
**Dnajc3**	-1,2639504	0,0000000	0,00000000
**Tcof1**	-1,2604584	0,0000000	0,00000000
**Cenpp**	-1,2570019	0,0000000	0,00000000
**Ccdc88c**	-1,2560564	0,0000000	0,00000000
**Rad21**	-1,2556611	0,0000000	0,00000000
**Ube2s**	-1,2549193	0,0000000	0,00000000
**Foxd2os**	-1,2542571	0,0000009	0,00000014
**Mroh2a**	-1,2534542	0,0000002	0,00000004
**Zbed3**	-1,2520683	0,0000000	0,00000000
**Gm4617**	-1,2473962	0,0000000	0,00000000
**Dctpp1**	-1,2449474	0,0000000	0,00000000
**Nup85**	-1,244719	0,0000000	0,00000000
**Ccdc138**	-1,2446733	0,0001174	0,00002492
**Rab11fip4**	-1,243218	0,0000000	0,00000000
**Card14**	-1,2430993	0,0000000	0,00000000
**Ska2**	-1,2372181	0,0000000	0,00000000
**Nrm**	-1,2327228	0,0000002	0,00000002
**Srrt**	-1,2296633	0,0000000	0,00000000
**Pfkl**	-1,2293757	0,0000000	0,00000000
**2010204K13Rik**	-1,229308	0,0000000	0,00000000
**Gm5898**	-1,2292594	0,0000000	0,00000000
**Gm9800**	-1,2284225	0,0000000	0,00000000

**Rangap1**	-1,2256575	0,0000000	0,00000000
**Ankrd44**	-1,2248737	0,0000000	0,00000000
**6720489N17Rik**	-1,2242665	0,0000492	0,00000983
**Rasd2**	-1,2238508	0,0001161	0,00002461
**Insc**	-1,223542	0,0000000	0,00000000
**Axin2**	-1,2215192	0,0000006	0,00000009
**Tfdp1**	-1,220881	0,0000000	0,00000000
**Evc**	-1,2177802	0,0000000	0,00000000
**Polr3g**	-1,2175973	0,0000000	0,00000000
**Gm5903**	-1,2175243	0,0000000	0,00000000
**Galk1**	-1,2165457	0,0000000	0,00000000
**Suv39h2**	-1,2161961	0,0000000	0,00000000
**Paics**	-1,2158727	0,0000000	0,00000000
**D130058E05Rik**	-1,2151701	0,0000000	0,00000000
**AU020206**	-1,2137479	0,0000000	0,00000000
**Gm16216**	-1,2130903	0,0017127	0,00045300
**Zfp651**	-1,2055753	0,0000000	0,00000000
**Epop**	-1,2054813	0,0000000	0,00000000
**Pcnt**	-1,2049721	0,0000000	0,00000000
**Lpcat1**	-1,2045146	0,0000000	0,00000000
**4930526A20Rik**	-1,2025551	0,0000071	0,00000126
**Idh2**	-1,201506	0,0000000	0,00000000
**C77080**	-1,2014944	0,0005002	0,00011961
**H3c15**	-1,2005739	0,0000155	0,00000285
**Ssbp4**	-1,200562	0,0000000	0,00000000
**Exosc2**	-1,196803	0,0000000	0,00000000
**Gm4430**	-1,1947201	0,0002126	0,00004711
**Miip**	-1,1942858	0,0000000	0,00000000
**Scrib**	-1,1925872	0,0000000	0,00000000
**Bri3bp**	-1,1902453	0,0000000	0,00000000
**Bri3**	-1,1899258	0,0000000	0,00000000
**Hk2**	-1,1881508	0,0000000	0,00000000
**Ccdc18**	-1,1873121	0,0002536	0,00005712
**H1f2**	-1,1857038	0,0000000	0,00000000
**Dhrs2**	-1,1850438	0,0000000	0,00000000
**Hyls1**	-1,1848636	0,0000000	0,00000000
**Bsn**	-1,1837534	0,0000000	0,00000000
**Aif1l**	-1,1780457	0,0000000	0,00000000
**Zfp382**	-1,1770634	0,0000001	0,00000001
**Etaa1**	-1,1757215	0,0000000	0,00000000
**Itga9**	-1,175323	0,0000000	0,00000000
**Car14**	-1,1736306	0,0000000	0,00000000

**Tmem50b**	-1,1722466	0,0000000	0,00000000
**Dstyk**	-1,1717213	0,0000000	0,00000000
**Tpgs2**	-1,168591	0,0000000	0,00000000
**Gm26448**	-1,1682897	0,0000000	0,00000000
**Haus5**	-1,1662879	0,0002577	0,00005811
**G2e3**	-1,16546	0,0000000	0,00000000
**Tsen34**	-1,1650226	0,0000000	0,00000000
**Aph1c**	-1,1636941	0,0000000	0,00000000
**Rfc2**	-1,1634602	0,0000000	0,00000000
**Rfwd3**	-1,1624836	0,0000000	0,00000000
**Gm8652**	-1,1621373	0,0007334	0,00018136
**Dhrs13**	-1,1618901	0,0000000	0,00000000
**Hs3st1**	-1,1601112	0,0007028	0,00017292
**Dct**	-1,1580833	0,0000000	0,00000000
**Nin**	-1,1574237	0,0000000	0,00000000
**Tcf3**	-1,1571831	0,0000000	0,00000000
**Siah1b**	-1,1555065	0,0000010	0,00000016
**Csrnp2**	-1,1546465	0,0000000	0,00000000
**Cmtm5**	-1,1544423	0,0007196	0,00017771
**Rnf26**	-1,1520139	0,0000000	0,00000000
**Ryr3**	-1,1513524	0,0000028	0,00000047
**Klf15**	-1,1512752	0,0000000	0,00000000
**Gm11246**	-1,1500511	0,0000102	0,00000183
**Gnptab**	-1,1491453	0,0000000	0,00000000
**Mipol1**	-1,1477726	0,0000000	0,00000000
**Kit**	-1,1473852	0,0000000	0,00000000
**Kif26a**	-1,1465698	0,0001646	0,00003581
**Zw10**	-1,1459843	0,0000000	0,00000000
**Lsm5**	-1,1457486	0,0000000	0,00000000
**Fam111a**	-1,1444437	0,0000000	0,00000000
**Grk6**	-1,1441873	0,0000000	0,00000000
**Fkbp11**	-1,1427024	0,0000000	0,00000001
**Med12l**	-1,1395644	0,0000000	0,00000000
**Evl**	-1,1392006	0,0000000	0,00000000
**Ano3**	-1,1348132	0,0000000	0,00000000
**Hsp90b1**	-1,1318835	0,0000000	0,00000000
**Ephb4**	-1,1302456	0,0000000	0,00000000
**Ppp1r16b**	-1,1300929	0,0000000	0,00000000
**Cfap20**	-1,1291675	0,0000000	0,00000000
**Hpgds**	-1,1291641	0,0000000	0,00000000
**Dixdc1**	-1,1240565	0,0000000	0,00000000
**Pou6f1**	-1,1240087	0,0000000	0,00000000

**Zmiz2**	-1,121528	0,0000000	0,00000000
**Cpsf6**	-1,1201555	0,0000000	0,00000000
**Adi1**	-1,1192466	0,0000000	0,00000000
**Rfc3**	-1,1192048	0,0000000	0,00000000
**Gm9256**	-1,118874	0,0001437	0,00003093
**Mbd4**	-1,1187781	0,0000000	0,00000000
**Epb41l4b**	-1,1175525	0,0000019	0,00000031
**Ptma**	-1,1173661	0,0000000	0,00000000
**Sms-ps**	-1,116643	0,0000000	0,00000000
**Lpgat1**	-1,1157473	0,0000000	0,00000000
**Mllt6**	-1,1156103	0,0000000	0,00000000
**Tbc1d31**	-1,1151057	0,0000000	0,00000000
**Rbm14**	-1,1149604	0,0000000	0,00000000
**Xndc1**	-1,1140353	0,0000688	0,00001411
**Prim2**	-1,1121768	0,0000000	0,00000000
**L3hypdh**	-1,1097059	0,0000000	0,00000000
**Uaca**	-1,1094839	0,0000000	0,00000000
**Alyref**	-1,1094653	0,0000000	0,00000000
**Prelid2**	-1,1088639	0,0000026	0,00000043
**Cdc42ep5**	-1,1073064	0,0000000	0,00000000
**Dennd11**	-1,1056225	0,0000000	0,00000001
**Cdkn2d**	-1,1044749	0,0000000	0,00000000
**Cep295**	-1,1040759	0,0000000	0,00000000
**Chd1l**	-1,1021139	0,0000000	0,00000000
**Rapgef1**	-1,1006912	0,0000000	0,00000000
**Gm4204**	-1,1004745	0,0000000	0,00000000
**Rtel1**	-1,1002668	0,0000000	0,00000000
**Mphosph9**	-1,0997624	0,0000000	0,00000000
**Rab27a**	-1,0995786	0,0000000	0,00000000
**Pdia4**	-1,0991713	0,0000000	0,00000000
**Mcmbp**	-1,0987098	0,0000000	0,00000000
**Celsr2**	-1,0985377	0,0000000	0,00000000
**Srm**	-1,0983752	0,0000000	0,00000000
**Scml2**	-1,0983454	0,0011238	0,00028687
**St6galnac3**	-1,0974567	0,0000000	0,00000000
**Reep4**	-1,0969022	0,0000000	0,00000000
**Cbx3**	-1,095573	0,0000000	0,00000000
**C330018A13Rik**	-1,0937059	0,0000573	0,00001159
**Sclt1**	-1,0936687	0,0000001	0,00000001
**Crocc**	-1,0927178	0,0000000	0,00000000
**Mdc1**	-1,0921785	0,0000000	0,00000000
**Zfp395**	-1,0919161	0,0000001	0,00000001

**Mbnl3**	-1,0916495	0,0000000	0,00000000
**Ak3l2-ps**	-1,0912183	0,0001080	0,00002280
**Gxylt1**	-1,088497	0,0000000	0,00000000
**Ppp5c**	-1,0877707	0,0000000	0,00000000
**Msh2**	-1,0873271	0,0000000	0,00000000
**Myo5a**	-1,0872796	0,0000000	0,00000000
**Telo2**	-1,0872066	0,0000000	0,00000000
**Adamts10**	-1,0857418	0,0000000	0,00000000
**L3mbtl2**	-1,0845031	0,0000000	0,00000000
**Lrrc4b**	-1,0839927	0,0000000	0,00000000
**Dpy30**	-1,0836161	0,0000000	0,00000000
**Mcm8**	-1,0808227	0,0000000	0,00000000
**Nup107**	-1,0806382	0,0000000	0,00000000
**Hmces**	-1,0804799	0,0000000	0,00000000
**Zc3hav1l**	-1,0799935	0,0000000	0,00000000
**Nup205**	-1,0782937	0,0000000	0,00000000
**Plxnd1**	-1,077397	0,0000000	0,00000000
**Prex2**	-1,0771887	0,0000000	0,00000000
**Slc45a2**	-1,0756319	0,0000000	0,00000000
**Nup37**	-1,0754255	0,0000000	0,00000000
**Rgma**	-1,0752775	0,0000000	0,00000000
**Arhgap10**	-1,0747068	0,0000000	0,00000000
**Sirpa**	-1,074646	0,0000000	0,00000000
**Dcps**	-1,0722326	0,0000000	0,00000000
**Arrb2**	-1,0721591	0,0000000	0,00000000
**Cyba**	-1,0721098	0,0000000	0,00000000
**Gm5643**	-1,071088	0,0018403	0,00049018
**Cln6**	-1,0707426	0,0000000	0,00000000
**Pdia6**	-1,0702522	0,0000000	0,00000000
**Gm10052**	-1,0691454	0,0000000	0,00000000
**Tpcn1**	-1,0685825	0,0000000	0,00000000
**Prps1**	-1,068535	0,0000000	0,00000000
**Rnaseh2a**	-1,0684469	0,0000000	0,00000000
**Gtf2h1**	-1,06312	0,0000000	0,00000000
**Srsf4**	-1,0623892	0,0000000	0,00000000
**Zcchc8**	-1,0584608	0,0000000	0,00000000
**Cfap298**	-1,0582918	0,0000000	0,00000000
**Nup210l**	-1,0572988	0,0017713	0,00046985
**Pnn**	-1,0572476	0,0000000	0,00000000
**Palm**	-1,0571799	0,0000000	0,00000000
**1700019D03Rik**	-1,0571096	0,0014655	0,00038268
**Baz1b**	-1,0568089	0,0000000	0,00000000

**Gm6682**	-1,0546043	0,0000041	0,00000069
**Gins3**	-1,0544405	0,0000000	0,00000000
**Col9a1**	-1,0542262	0,0000709	0,00001456
**Gm6166**	-1,053476	0,0000000	0,00000000
**Atm**	-1,0523103	0,0000000	0,00000000
**Fgd1**	-1,0500457	0,0000000	0,00000000
**Sh3bp1**	-1,0499706	0,0000000	0,00000000
**Spcs3**	-1,0493956	0,0000000	0,00000000
**Zkscan17**	-1,0486253	0,0000000	0,00000000
**Fzr1**	-1,0485424	0,0000000	0,00000000
**Fbxo31**	-1,0484582	0,0000000	0,00000000
**G0s2**	-1,0478461	0,0000671	0,00001373
**Cmc2**	-1,0451193	0,0000000	0,00000000
**Tuba1c**	-1,0450986	0,0000000	0,00000000
**Stau2**	-1,0446402	0,0000000	0,00000000
**3110082I17Rik**	-1,0445583	0,0000000	0,00000000
**Nolc1**	-1,0444056	0,0000000	0,00000000
**Ttbk1**	-1,0436792	0,0000000	0,00000000
**Cfap410**	-1,0424364	0,0000000	0,00000000
**Snrpa1**	-1,041913	0,0000000	0,00000000
**Ckap2**	-1,0410329	0,0000000	0,00000000
**Gm11353**	-1,0402162	0,0004100	0,00009642
**Hnrnpab**	-1,0399136	0,0000000	0,00000000
**Zik1**	-1,0394104	0,0000000	0,00000000
**Gnb1l**	-1,0391121	0,0000066	0,00000115
**Nfkbib**	-1,0389637	0,0000000	0,00000000
**Pgam1-ps2**	-1,0367433	0,0000000	0,00000000
**Mrpl12**	-1,0367387	0,0000000	0,00000000
**Slc7a5**	-1,0365982	0,0000000	0,00000000
**Donson**	-1,036301	0,0000000	0,00000000
**Gm7846**	-1,033654	0,0000006	0,00000009
**Nova1**	-1,033082	0,0000036	0,00000061
**Elapor2**	-1,0319341	0,0000013	0,00000020
**Zmynd19**	-1,030808	0,0000000	0,00000000
**Get4**	-1,029791	0,0000000	0,00000000
**Sec61a2**	-1,0296645	0,0000000	0,00000000
**Nfya**	-1,0295983	0,0000000	0,00000000
**Fam53b**	-1,029552	0,0000000	0,00000000
**Ncaph2**	-1,0295335	0,0000000	0,00000000
**Ddx39a**	-1,0279413	0,0000000	0,00000000
**1700066M21Rik**	-1,0269416	0,0000001	0,00000002
**Nt5c2**	-1,0239182	0,0000000	0,00000000

**Arfgef3**	-1,0236564	0,0000000	0,00000000
**Cort**	-1,0235975	0,0008348	0,00020841
**2700038G22Rik**	-1,0231082	0,0000192	0,00000359
**Lsm2**	-1,0226708	0,0000000	0,00000000
**Epb41**	-1,0223414	0,0000000	0,00000000
**Pgap1**	-1,0207285	0,0000000	0,00000000
**Mif-ps4**	-1,0205697	0,0000000	0,00000000
**Nap1l1**	-1,0190144	0,0000000	0,00000000
**Stk39**	-1,0179488	0,0000000	0,00000000
**Dnaaf2**	-1,0166116	0,0000000	0,00000000
**Epm2a**	-1,0161448	0,0015183	0,00039757
**Fut10**	-1,0158727	0,0000000	0,00000000
**Lrrc8b**	-1,0143428	0,0000000	0,00000000
**4930503L19Rik**	-1,014311	0,0000000	0,00000000
**Sgsm2**	-1,0137992	0,0000000	0,00000000
**Slc9a5**	-1,0134848	0,0005455	0,00013151
**Casp8ap2**	-1,0131164	0,0000000	0,00000000
**Lmx1b**	-1,0121199	0,0000000	0,00000000
**Rpa1**	-1,0119965	0,0000000	0,00000000
**Orc6**	-1,0116128	0,0000000	0,00000000
**Prag1**	-1,0104798	0,0000000	0,00000000
**Plekha8**	-1,0094914	0,0000000	0,00000000
**Smc5**	-1,0086911	0,0000000	0,00000000
**Sae1**	-1,0058299	0,0000000	0,00000000
**Sfpq**	-1,0030329	0,0000000	0,00000000
**Myo1h**	-1,0029867	0,0005711	0,00013816
**Myc**	-1,0012896	0,0000000	0,00000000
**Srsf7**	-1,0010466	0,0000000	0,00000000
**Tex30**	-1,0008965	0,0000000	0,00000000
**Dusp8**	1,00043071	0,0000000	0,00000001
**Zdhhc1**	1,00063261	0,0004801	0,00011450
**Micall2**	1,00124565	0,0000001	0,00000002
**Tmco6**	1,00168131	0,0003875	0,00009078
**Myl12a**	1,00294026	0,0000000	0,00000000
**Zwint**	1,00372494	0,0000000	0,00000000
**Slc35d2**	1,00555842	0,0000755	0,00001559
**Jrkl**	1,00725244	0,0000108	0,00000195
**Glt8d1**	1,00812199	0,0000000	0,00000000
**Maff**	1,00871755	0,0000002	0,00000003
**Tefm**	1,00942985	0,0000000	0,00000000
**Psme1**	1,01066735	0,0000000	0,00000000
**Gm7809**	1,01246776	0,0000000	0,00000000

**Car8**	1,01629641	0,0013296	0,00034446
**B230354K17Rik**	1,01634553	0,0000000	0,00000000
**Enpp1**	1,0166091	0,0008810	0,00022062
**E130311K13Rik**	1,01717476	0,0000154	0,00000282
**Coq8a**	1,01792993	0,0000000	0,00000000
**Hmgcs1**	1,0187606	0,0000000	0,00000000
**Snapc5**	1,01896769	0,0000000	0,00000000
**Snhg15**	1,01980249	0,0000000	0,00000000
**Eola1**	1,02174124	0,0000006	0,00000009
**Zfp945**	1,0219967	0,0000000	0,00000000
**Rcan1**	1,02249313	0,0000000	0,00000000
**Lrp1**	1,02448807	0,0000000	0,00000000
**Arhgap28**	1,02473254	0,0000000	0,00000000
**Nagk**	1,02570307	0,0000000	0,00000000
**Ccnd2**	1,02579263	0,0000000	0,00000000
**Tspo**	1,02605972	0,0000000	0,00000000
**Abca7**	1,02668339	0,0000000	0,00000000
**2310039H08Rik**	1,02777739	0,0000000	0,00000000
**Gale**	1,02797158	0,0000000	0,00000000
**Crabp2**	1,02875202	0,0002121	0,00004697
**Qsox1**	1,02897424	0,0000000	0,00000000
**Ctxn1**	1,0291022	0,0000000	0,00000000
**Speg**	1,0296234	0,0000000	0,00000000
**Stat3**	1,03123086	0,0000000	0,00000000
**3000002C10Rik**	1,0319459	0,0004025	0,00009452
**Klf6**	1,03241875	0,0000000	0,00000000
**Pdp1**	1,03420544	0,0000062	0,00000108
**Oaz2**	1,03433967	0,0000000	0,00000000
**Lipa**	1,03494437	0,0000000	0,00000000
**Ston1**	1,03706152	0,0000000	0,00000000
**Runx1**	1,03720356	0,0000000	0,00000000
**Rmdn2**	1,03805536	0,0000002	0,00000003
**Klrg2**	1,03974083	0,0002670	0,00006047
**Zswim6**	1,04005295	0,0000000	0,00000000
**Zbtb38**	1,04033607	0,0000000	0,00000000
**Psmb10**	1,04085487	0,0000000	0,00000000
**Kctd21**	1,04119582	0,0015294	0,00040068
**Col20a1**	1,04160583	0,0000191	0,00000356
**Decr2**	1,04162029	0,0000000	0,00000000
**Tapbpl**	1,04236044	0,0000051	0,00000088
**Tgfb2**	1,04271052	0,0000000	0,00000000
**Tpm1**	1,04329331	0,0000000	0,00000000

**Ccdc73**	1,04330855	0,0004311	0,00010171
**Zfp119b**	1,0447741	0,0001152	0,00002440
**F8a**	1,04514077	0,0000008	0,00000013
**Cfap157**	1,0451841	0,0011030	0,00028110
**Hint2**	1,05067008	0,0000001	0,00000001
**Efcab2**	1,0533849	0,0000007	0,00000011
**Itpripl2**	1,0534968	0,0000000	0,00000000
**Dcaf8l**	1,05416709	0,0000000	0,00000000
**Btg1**	1,05429467	0,0000000	0,00000000
**Hpcal1**	1,05704661	0,0000000	0,00000000
**Serpinb6a**	1,05714632	0,0000000	0,00000000
**Rhod**	1,05794384	0,0000000	0,00000000
**Gm6222**	1,05886322	0,0000664	0,00001356
**Slc35f5**	1,06012651	0,0000000	0,00000000
**Tpm4**	1,06332669	0,0000000	0,00000000
**Lnpep**	1,06457401	0,0000000	0,00000000
**Smim10l1**	1,06525809	0,0000000	0,00000000
**Myd88**	1,0661068	0,0000000	0,00000000
**Ppp1r18**	1,06632563	0,0000000	0,00000000
**Pla2r1**	1,06636274	0,0000000	0,00000000
**Lasp1**	1,06738818	0,0000000	0,00000000
**Zfp703**	1,07032193	0,0000000	0,00000000
**Dtna**	1,07043464	0,0000000	0,00000000
**Lhfpl2**	1,071938	0,0000000	0,00000000
**Cd9**	1,07207248	0,0000000	0,00000000
**Htatip2**	1,07209617	0,0000000	0,00000000
**Ddit4**	1,07216476	0,0000000	0,00000000
**Plpp6**	1,0738764	0,0000000	0,00000000
**Zfp874b**	1,07698263	0,0000003	0,00000004
**Calhm5**	1,07733699	0,0000015	0,00000024
**Creb3l1**	1,07768461	0,0003229	0,00007446
**Acox3**	1,07796575	0,0000000	0,00000000
**Zbtb4**	1,08080663	0,0000000	0,00000000
**Acot2**	1,08184632	0,0000000	0,00000000
**Chpf2**	1,08212951	0,0000000	0,00000000
**Cryab**	1,08219054	0,0000000	0,00000000
**Gcnt1**	1,08290865	0,0000000	0,00000000
**Pgghg**	1,08340564	0,0000000	0,00000000
**Sfxn5**	1,08371551	0,0000000	0,00000000
**Gpr146**	1,08457316	0,0000000	0,00000000
**Snhg8**	1,0868262	0,0000000	0,00000000
**S100a1**	1,08704322	0,0000000	0,00000000

**Ctsb**	1,08753713	0,0000000	0,00000000
**Gstm5**	1,08777863	0,0000000	0,00000000
**Rps6ka3**	1,08779084	0,0000000	0,00000000
**Neil1**	1,08821057	0,0000155	0,00000286
**Nsdhl**	1,08858752	0,0000000	0,00000000
**Lpin1**	1,08890168	0,0001611	0,00003497
**Tpp1**	1,08912418	0,0000000	0,00000000
**Pigm**	1,09114101	0,0000000	0,00000000
**Gm10080**	1,09170499	0,0000000	0,00000000
**Lztfl1**	1,09178077	0,0000000	0,00000001
**Psrc1**	1,09179473	0,0000000	0,00000000
**Glb1l**	1,0938465	0,0013727	0,00035648
**Slc25a24**	1,09573279	0,0000000	0,00000000
**Snord104**	1,09614843	0,0000000	0,00000000
**Bcas3**	1,09672051	0,0000000	0,00000000
**1700086O06Rik**	1,09772587	0,0000541	0,00001090
**Gstt3**	1,09833824	0,0000442	0,00000876
**Lats2**	1,10023316	0,0000000	0,00000000
**Gpr153**	1,10081164	0,0000224	0,00000421
**Skil**	1,10085851	0,0000000	0,00000000
**Cpeb2**	1,10245893	0,0000000	0,00000000
**Orai2**	1,10306516	0,0000000	0,00000000
**Cd151**	1,10503091	0,0000000	0,00000000
**Rtn4**	1,10509493	0,0000000	0,00000000
**Rtn2**	1,10735865	0,0000023	0,00000037
**Gpcpd1**	1,10853683	0,0000000	0,00000000
**Rock2**	1,1091507	0,0000000	0,00000000
**C430049B03Rik**	1,11026451	0,0007398	0,00018310
**Dnajb2**	1,11078071	0,0000000	0,00000000
**Btbd19**	1,11270185	0,0000000	0,00000000
**Plxna3**	1,11333169	0,0000488	0,00000975
**Crip1**	1,11522434	0,0012066	0,00031026
**Hint3**	1,11538636	0,0000248	0,00000471
**Prorsd1**	1,11614128	0,0000000	0,00000000
**Ttc30b**	1,11702588	0,0000360	0,00000703
**Wdr5b**	1,11743441	0,0000515	0,00001034
**Pmp22**	1,11768365	0,0000000	0,00000000
**Pycr1**	1,11906522	0,0000025	0,00000042
**Cd81**	1,11932684	0,0000000	0,00000000
**Boc**	1,121744	0,0000000	0,00000000
**Jade1**	1,12180441	0,0000000	0,00000000
**Basp1**	1,12189754	0,0000000	0,00000000

**Plec**	1,12229126	0,0000584	0,00001184
**Tmem43**	1,12257478	0,0000000	0,00000000
**Bphl**	1,12262711	0,0000000	0,00000000
**Gm38317**	1,12274537	0,0004195	0,00009879
**Pck2**	1,1233986	0,0000000	0,00000000
**4930429F24Rik**	1,12381597	0,0000103	0,00000185
**Gnb4**	1,12535207	0,0000000	0,00000000
**Arsg**	1,12582422	0,0000009	0,00000014
**Rabac1**	1,12633379	0,0000000	0,00000000
**Mdm2**	1,12772612	0,0000000	0,00000000
**Ccndbp1**	1,12800638	0,0000000	0,00000000
**Lrrc24**	1,12887975	0,0000000	0,00000000
**1600014C10Rik**	1,12998588	0,0000000	0,00000000
**Gm19426**	1,13011243	0,0000513	0,00001029
**Coa4**	1,13025153	0,0000104	0,00000188
**Cyp26b1**	1,13044704	0,0000000	0,00000000
**Rhobtb3**	1,13160179	0,0000000	0,00000000
**Atp6v0a4**	1,13372675	0,0000038	0,00000065
**Dnase1l1**	1,13445414	0,0001942	0,00004271
**Ufsp1**	1,13592178	0,0000230	0,00000434
**E430018J23Rik**	1,13600302	0,0010400	0,00026398
**Gpsm3**	1,13618376	0,0000000	0,00000000
**Tnfrsf10b**	1,13712324	0,0000000	0,00000000
**Sdr42e1**	1,13764225	0,0004515	0,00010710
**Fam102a**	1,13838533	0,0000000	0,00000000
**Ier5**	1,13983935	0,0000000	0,00000000
**Gm4875**	1,14097287	0,0002947	0,00006741
**Gask1b**	1,14439433	0,0000000	0,00000000
**Arhgap26**	1,14619896	0,0000000	0,00000000
**Lifr**	1,14654338	0,0000070	0,00000124
**Itgb5**	1,14731328	0,0000000	0,00000000
**Hexim1**	1,14888454	0,0000000	0,00000000
**Usp27x**	1,14974318	0,0000012	0,00000019
**Wbp1**	1,15315031	0,0000000	0,00000000
**Trappc2**	1,15333379	0,0000051	0,00000089
**Tram1l1**	1,15416093	0,0000008	0,00000013
**Tnfaip3**	1,15449223	0,0010230	0,00025943
**Zbtb26**	1,15555203	0,0000000	0,00000000
**Tmem176a**	1,15696274	0,0000000	0,00000000
**Rcn3**	1,15912838	0,0000209	0,00000393
**Gm29083**	1,15991768	0,0018252	0,00048572
**Ubald1**	1,16185937	0,0000000	0,00000000

**Relb**	1,16235881	0,0000000	0,00000000
**Plaat3**	1,16566255	0,0000000	0,00000000
**Prokr1**	1,16624303	0,0000000	0,00000000
**Zfp874a**	1,16657049	0,0000000	0,00000000
**Gstm2**	1,16770141	0,0000000	0,00000000
**Slc5a1**	1,1682268	0,0005946	0,00014438
**Dubr**	1,16982677	0,0000000	0,00000000
**E130317F20Rik**	1,17008169	0,0000138	0,00000253
**C1rl**	1,17382055	0,0000030	0,00000051
**Atxn1**	1,17471135	0,0000000	0,00000000
**Slc15a2**	1,17524899	0,0000000	0,00000000
**Gpm6b**	1,17646433	0,0000620	0,00001263
**Usp20**	1,17692282	0,0000000	0,00000000
**Csf2ra**	1,1784671	0,0000261	0,00000499
**Dpy19l3**	1,17910268	0,0000000	0,00000000
**Gm6665**	1,18000835	0,0000000	0,00000000
**Lpp**	1,18084114	0,0000000	0,00000000
**Qpctl**	1,18111796	0,0000000	0,00000000
**Rdh5**	1,18269855	0,0001371	0,00002940
**Vim**	1,18420154	0,0000000	0,00000000
**Loxl2**	1,18683843	0,0000074	0,00000131
**Zfp57**	1,18935549	0,0000005	0,00000008
**H2bc21**	1,18967118	0,0000000	0,00000000
**Klf2**	1,19043808	0,0000580	0,00001174
**Camk2d**	1,19067404	0,0000000	0,00000000
**Vgll3**	1,19154621	0,0000000	0,00000000
**Cpeb3**	1,19187274	0,0000000	0,00000000
**Adamts13**	1,19320239	0,0003698	0,00008623
**Mcts2**	1,19333791	0,0000000	0,00000000
**Serf1**	1,19351897	0,0000000	0,00000000
**Trim7**	1,19521632	0,0000456	0,00000905
**Otub2**	1,19742576	0,0000000	0,00000000
**R74862**	1,19862267	0,0000007	0,00000011
**Ache**	1,20175939	0,0000040	0,00000067
**Atf3**	1,20324056	0,0000000	0,00000000
**2900041M22Rik**	1,20549074	0,0002763	0,00006275
**Pcdhb5**	1,20697815	0,0000072	0,00000127
**Aldh1a3**	1,20760389	0,0000000	0,00000000
**Pcdhb17**	1,20773071	0,0000090	0,00000161
**Klhl29**	1,21031299	0,0000000	0,00000000
**Khk**	1,21046553	0,0000000	0,00000000
**Alpk1**	1,21117383	0,0000002	0,00000003

**Inppl1**	1,21387036	0,0000000	0,00000000
**Coq10b**	1,21608954	0,0000000	0,00000000
**Wipf3**	1,21763812	0,0000000	0,00000000
**Pik3ip1**	1,21997299	0,0000981	0,00002059
**Klf9**	1,22059256	0,0000000	0,00000000
**Csrnp1**	1,22099908	0,0000000	0,00000000
**Fars2**	1,22155139	0,0000000	0,00000000
**Rab39b**	1,22168976	0,0000526	0,00001058
**Usp11**	1,22231824	0,0000000	0,00000000
**Nat9**	1,22283912	0,0000000	0,00000000
**Acbd4**	1,22299971	0,0000000	0,00000000
**Itm2b**	1,22420427	0,0000000	0,00000000
**Cpm**	1,22633735	0,0000000	0,00000000
**Nfil3**	1,22763325	0,0000000	0,00000000
**Pdrg1**	1,22835641	0,0000000	0,00000000
**Hap1**	1,2285804	0,0000000	0,00000000
**Inafm2**	1,2323184	0,0000000	0,00000000
**Casq2**	1,23359095	0,0002738	0,00006217
**Msrb1**	1,2340246	0,0000000	0,00000000
**Rsu1**	1,23453843	0,0000000	0,00000000
**Gstk1**	1,23485925	0,0002834	0,00006459
**Erap1**	1,23501457	0,0000000	0,00000000
**Exd1**	1,23569311	0,0012725	0,00032845
**Plekhn1**	1,23643386	0,0018370	0,00048919
**Flna**	1,23700614	0,0000000	0,00000000
**Mob3a**	1,23827742	0,0000618	0,00001256
**Crem**	1,23910759	0,0000000	0,00000000
**Mrps6**	1,24107088	0,0000000	0,00000000
**Sat2**	1,24135971	0,0000327	0,00000634
**Smim14**	1,24146691	0,0000000	0,00000000
**Ckb**	1,24153222	0,0000000	0,00000000
**Marchf3**	1,24215008	0,0009136	0,00022935
**Ahr**	1,24409915	0,0000000	0,00000000
**Cyld**	1,24412638	0,0000000	0,00000000
**Dhrs1**	1,24639561	0,0000000	0,00000000
**Tmem198b**	1,24720356	0,0000000	0,00000000
**Asah2**	1,24756673	0,0000000	0,00000000
**Lrp11**	1,2475999	0,0000000	0,00000000
**Pigp**	1,24766926	0,0000000	0,00000000
**Mertk**	1,24830768	0,0000046	0,00000078
**Gm10825**	1,24928516	0,0000021	0,00000035
**Soga3**	1,24936894	0,0002135	0,00004735

**Trmt12**	1,25042685	0,0000000	0,00000000
**Dock8**	1,25274455	0,0000000	0,00000000
**Cyp2u1**	1,2527853	0,0000000	0,00000000
**Cirbp**	1,25323395	0,0000000	0,00000000
**Slc44a3**	1,25896043	0,0000038	0,00000064
**Gxylt2**	1,26024503	0,0000000	0,00000000
**Cldn12**	1,26084543	0,0000000	0,00000000
**Aff1**	1,2622195	0,0000000	0,00000000
**A730017L22Rik**	1,26472449	0,0000002	0,00000003
**Epm2aip1**	1,26485198	0,0000000	0,00000000
**Bbs10**	1,26529338	0,0000002	0,00000003
**Eva1c**	1,26560025	0,0000239	0,00000453
**P3h2**	1,26606981	0,0000000	0,00000000
**Slc10a3**	1,26633663	0,0000000	0,00000000
**Etv5**	1,2666979	0,0000000	0,00000000
**Pxmp4**	1,26682576	0,0000000	0,00000000
**Acad10**	1,26725809	0,0000000	0,00000000
**Sesn2**	1,26882939	0,0000766	0,00001584
**2010320M18Rik**	1,26896126	0,0000000	0,00000000
**Pgam2**	1,26935369	0,0010325	0,00026194
**Mcam**	1,27018887	0,0000000	0,00000000
**Ptrh1**	1,27071537	0,0000012	0,00000019
**Shisa4**	1,27309814	0,0000000	0,00000000
**Pcx**	1,27396053	0,0000000	0,00000000
**Gm37352**	1,27430054	0,0000000	0,00000000
**Anxa11**	1,27464085	0,0000000	0,00000000
**Trim45**	1,27642804	0,0000073	0,00000129
**Exoc3l4**	1,28102042	0,0000266	0,00000509
**Tdrd7**	1,2821395	0,0000000	0,00000000
**Tspan17**	1,28380394	0,0000000	0,00000000
**Ebf4**	1,2846221	0,0007723	0,00019187
**Pde5a**	1,28541889	0,0000000	0,00000000
**Noct**	1,28694001	0,0000000	0,00000000
**Idi1-ps1**	1,28746444	0,0000000	0,00000000
**Kif6**	1,29213466	0,0000000	0,00000000
**Zscan2**	1,29288847	0,0000000	0,00000000
**Cdkl2**	1,29306477	0,0000000	0,00000000
**Rims2**	1,29333593	0,0000000	0,00000000
**Col11a1**	1,29612935	0,0016826	0,00044430
**Acadvl**	1,29615982	0,0000000	0,00000000
**Tm7sf2**	1,29618043	0,0000058	0,00000102
**Avpi1**	1,29778411	0,0000000	0,00000000

**Aamdc**	1,29913364	0,0000000	0,00000000
**Bcar1**	1,29924591	0,0000000	0,00000000
**Gm572**	1,2994321	0,0008765	0,00021945
**Ly6g5c**	1,30294933	0,0004184	0,00009851
**Pttg1**	1,30327608	0,0000000	0,00000000
**Ehd4**	1,30603795	0,0000000	0,00000000
**0610038B21Rik**	1,30684865	0,0000093	0,00000166
**Slc9a9**	1,30852909	0,0000000	0,00000000
**Lancl1**	1,30914501	0,0000000	0,00000000
**Carmn**	1,31061479	0,0016978	0,00044864
**Nuak1**	1,31116836	0,0000000	0,00000000
**Alcam**	1,31304602	0,0000000	0,00000000
**Sh3tc1**	1,31306519	0,0009801	0,00024746
**Il13ra1**	1,31405215	0,0000000	0,00000000
**Blcap**	1,31499784	0,0000000	0,00000000
**Kcnd1**	1,31523696	0,0000112	0,00000203
**Unc93b1**	1,31533458	0,0000000	0,00000000
**Mmd**	1,31616757	0,0000000	0,00000000
**Ctsl**	1,31657628	0,0000000	0,00000000
**Dynlt1b**	1,31774326	0,0000029	0,00000048
**Pcdha11**	1,31779015	0,0009412	0,00023695
**Hspa4l**	1,317884	0,0000000	0,00000000
**Taf9b**	1,31852936	0,0000000	0,00000000
**Zcwpw1**	1,31858366	0,0000719	0,00001479
**Tafa5**	1,31862679	0,0000222	0,00000418
**Map7**	1,32017794	0,0002429	0,00005442
**Hbegf**	1,3225787	0,0000001	0,00000001
**Ubash3b**	1,32328209	0,0000000	0,00000000
**Ehd2**	1,32334609	0,0002658	0,00006014
**Tnrc18**	1,32347996	0,0000000	0,00000000
**Yjefn3**	1,32546638	0,0000001	0,00000001
**Id3**	1,32670964	0,0000000	0,00000000
**Gm10863**	1,32896108	0,0000000	0,00000000
**5930430L01Rik**	1,32996699	0,0005360	0,00012895
**Spaar**	1,3301941	0,0003674	0,00008556
**Acad11**	1,33085667	0,0000000	0,00000000
**Pld3**	1,33135891	0,0000000	0,00000000
**Hspa2**	1,33174934	0,0000000	0,00000000
**Crebl2**	1,33297753	0,0000000	0,00000000
**1600012H06Rik**	1,33300704	0,0000000	0,00000000
**F3**	1,33316217	0,0000000	0,00000000
**Nudt18**	1,33366761	0,0000000	0,00000000

**1110032A03Rik**	1,33438124	0,0000000	0,00000000
**Crebrf**	1,33590116	0,0000000	0,00000000
**Acyp1**	1,33678948	0,0000000	0,00000000
**Dusp6**	1,33821057	0,0000000	0,00000000
**1810024B03Rik**	1,33852789	0,0000099	0,00000178
**Dlg4**	1,33870583	0,0000000	0,00000000
**Spry4**	1,34294174	0,0000000	0,00000000
**Tmem80**	1,34428747	0,0000000	0,00000000
**Ntng2**	1,3443916	0,0017187	0,00045475
**Pakap**	1,34916681	0,0000299	0,00000576
**Tmtc2**	1,34960638	0,0000031	0,00000051
**Cyb5r3**	1,35034637	0,0000000	0,00000000
**Gm9791**	1,35046231	0,0000038	0,00000064
**Wrn**	1,35077483	0,0000000	0,00000000
**Rrm2b**	1,35393015	0,0000000	0,00000000
**Ypel4**	1,3554107	0,0000004	0,00000006
**Duox1**	1,35634509	0,0000018	0,00000030
**Cyb5d2**	1,35764632	0,0000000	0,00000000
**Daam2**	1,35778873	0,0000000	0,00000000
**Ppp1r14c**	1,35843235	0,0000022	0,00000035
**Tmem230**	1,3587987	0,0000000	0,00000000
**Myrfl**	1,36240769	0,0002138	0,00004743
**Anxa7**	1,3626737	0,0000000	0,00000000
**Fam234a**	1,36307605	0,0000000	0,00000000
**4930556M19Rik**	1,36506769	0,0001174	0,00002495
**Gdi1**	1,36715015	0,0000000	0,00000000
**Rnh1**	1,36870694	0,0000000	0,00000000
**Thnsl2**	1,36912253	0,0000005	0,00000008
**Kctd12**	1,3708295	0,0000000	0,00000000
**Mageh1**	1,37085239	0,0000006	0,00000009
**Ptp4a3**	1,37125095	0,0000000	0,00000000
**Gsta1**	1,37250769	0,0000000	0,00000000
**Akr1b8**	1,37275654	0,0000000	0,00000000
**Zfp365**	1,37302956	0,0000754	0,00001555
**Zyx**	1,37450647	0,0000000	0,00000000
**Pomc**	1,37505744	0,0014598	0,00038098
**5730471H19Rik**	1,37516701	0,0000001	0,00000001
**Fam160a1**	1,37807469	0,0000107	0,00000192
**Asic3**	1,37855986	0,0003031	0,00006947
**Gm38391**	1,3789155	0,0000067	0,00000118
**Gm9522**	1,38044279	0,0006941	0,00017055
**Myl6**	1,38134086	0,0000000	0,00000000

**Cavin1**	1,38227991	0,0000000	0,00000000
**Cx3cl1**	1,38346349	0,0000000	0,00000000
**Gm18609**	1,38671595	0,0000636	0,00001297
**Dstn**	1,3875504	0,0000000	0,00000000
**Bbc3**	1,387654	0,0000000	0,00000000
**Cited2**	1,38801386	0,0000000	0,00000000
**Bloc1s6**	1,38997691	0,0000000	0,00000000
**L1cam**	1,39198369	0,0000000	0,00000000
**Nmi**	1,39287214	0,0000000	0,00000000
**Srxn1**	1,39341551	0,0000000	0,00000000
**Trib3**	1,3935769	0,0000000	0,00000000
**Klhdc7a**	1,39475526	0,0003145	0,00007230
**1700052K11Rik**	1,394844	0,0000502	0,00001007
**Capn1**	1,39522868	0,0000000	0,00000000
**Fbxl20**	1,39548905	0,0000000	0,00000000
**Arid5a**	1,39581871	0,0000000	0,00000000
**Fzd5**	1,39604642	0,0000000	0,00000000
**Morrbid**	1,39754317	0,0000000	0,00000000
**Pdzd7**	1,39831672	0,0000000	0,00000000
**H1f0**	1,39979796	0,0000000	0,00000000
**Arl11**	1,40133659	0,0000000	0,00000000
**Carhsp1**	1,40189705	0,0000000	0,00000000
**Cspg4**	1,40215584	0,0000000	0,00000000
**Fam71f1**	1,40257043	0,0000000	0,00000000
**Adar**	1,40343106	0,0000000	0,00000000
**Snap47**	1,40620119	0,0000000	0,00000000
**Slc25a1**	1,40705003	0,0000000	0,00000000
**Sfn**	1,4075629	0,0000000	0,00000000
**Nox4**	1,40940856	0,0000000	0,00000000
**Gm45495**	1,41003041	0,0008277	0,00020653
**Larp1b**	1,41144621	0,0000000	0,00000000
**Pak6**	1,41313708	0,0000408	0,00000803
**Cdc42ep1**	1,41501625	0,0000000	0,00000000
**Itgb3**	1,4166197	0,0000000	0,00000000
**Tmem203**	1,41734392	0,0006975	0,00017149
**Rnasel**	1,41781535	0,0000000	0,00000000
**1500015A07Rik**	1,41886044	0,0000039	0,00000066
**Cyb561**	1,42032075	0,0003170	0,00007294
**Zfp467**	1,4251473	0,0000000	0,00000000
**Eif2ak2**	1,42678687	0,0000000	0,00000000
**Pyurf**	1,42773813	0,0000000	0,00000000
**Tmem14a**	1,42910566	0,0000000	0,00000000

**Jak3**	1,42910575	0,0000042	0,00000071
**Shb**	1,4310155	0,0000000	0,00000000
**Eno2**	1,43335289	0,0000000	0,00000000
**Gm28809**	1,43446414	0,0000001	0,00000002
**Pkia**	1,43515654	0,0000000	0,00000000
**Tnfaip2**	1,4354219	0,0000000	0,00000000
**Cyp4f17**	1,43685109	0,0000031	0,00000052
**Hacl1**	1,43689077	0,0000000	0,00000000
**Zc2hc1a**	1,43786408	0,0000000	0,00000000
**Aldoc**	1,43786855	0,0000000	0,00000000
**Macrod1**	1,4381545	0,0000000	0,00000000
**Large1**	1,43847709	0,0000000	0,00000000
**Cyb561a3**	1,4391793	0,0000000	0,00000001
**Zbed5**	1,44005474	0,0001595	0,00003456
**Lrrc51**	1,4410366	0,0000000	0,00000000
**Dlx6os1**	1,44197051	0,0000001	0,00000001
**Tmem35b**	1,44319599	0,0000239	0,00000453
**Nabp1**	1,44324327	0,0000000	0,00000000
**4921531C22Rik**	1,44417573	0,0000020	0,00000033
**Prob1**	1,44515656	0,0001735	0,00003785
**Gabra1**	1,4520923	0,0007734	0,00019222
**Gm13067**	1,4559745	0,0003779	0,00008828
**G6pdx**	1,456296	0,0000000	0,00000000
**Rb1**	1,4565192	0,0000000	0,00000000
**Mylk**	1,45802208	0,0000000	0,00000000
**Gstm4**	1,46223668	0,0000000	0,00000000
**Gm42778**	1,46300888	0,0007267	0,00017965
**2610306M01Rik**	1,46362201	0,0000000	0,00000000
**Kyat1**	1,46438903	0,0000000	0,00000000
**Cavin2**	1,46494955	0,0000000	0,00000000
**Akr1b3**	1,4653992	0,0000000	0,00000000
**Ctnnal1**	1,4683331	0,0000000	0,00000000
**2810013P06Rik**	1,46917065	0,0000000	0,00000000
**Tmem220**	1,47063237	0,0000452	0,00000898
**Dok5**	1,47082744	0,0000000	0,00000000
**Shc3**	1,47129936	0,0000000	0,00000000
**Ccdc63**	1,47130547	0,0011284	0,00028825
**Kif9**	1,47336388	0,0001518	0,00003280
**Pdlim5**	1,47403778	0,0000000	0,00000000
**Akr1b10**	1,4740399	0,0000000	0,00000000
**Ehd3**	1,47518193	0,0000000	0,00000000
**Gm42829**	1,47529679	0,0002480	0,00005571

**Dgka**	1,47893233	0,0000000	0,00000000
**Slc30a4**	1,47943816	0,0000000	0,00000000
**Gm7967**	1,48292842	0,0004732	0,00011267
**Dner**	1,48326022	0,0007164	0,00017686
**4930455G09Rik**	1,4832964	0,0000107	0,00000193
**B230206L02Rik**	1,4834033	0,0000000	0,00000000
**Draxin**	1,48436024	0,0001950	0,00004289
**Adamts1**	1,48582782	0,0000000	0,00000000
**Cmbl**	1,48654943	0,0000000	0,00000000
**Dyrk1b**	1,49254689	0,0000000	0,00000000
**1700007L15Rik**	1,49256464	0,0000465	0,00000926
**Fuz**	1,49670156	0,0000000	0,00000000
**Polr2l**	1,49723167	0,0000000	0,00000000
**Nacad**	1,4974076	0,0000002	0,00000003
**Tnfrsf14**	1,49891713	0,0008236	0,00020544
**Lrrc27**	1,4994076	0,0000000	0,00000000
**Gm6736**	1,49997153	0,0000000	0,00000000
**Klhdc1**	1,50209177	0,0000046	0,00000078
**Malat1**	1,50330046	0,0000000	0,00000000
**Csdc2**	1,503467	0,0000652	0,00001331
**B230216N24Rik**	1,50477659	0,0005116	0,00012258
**Gm37696**	1,50592801	0,0000283	0,00000544
**Arl4a**	1,5069684	0,0000000	0,00000000
**Rnf128**	1,50865799	0,0000000	0,00000000
**BC024978**	1,5097364	0,0000000	0,00000000
**Il18**	1,51159249	0,0004314	0,00010181
**Hdac9**	1,51165727	0,0000238	0,00000451
**Gfap**	1,51299034	0,0000015	0,00000025
**Spry2**	1,51426344	0,0000000	0,00000000
**Pbxip1**	1,51484493	0,0000000	0,00000000
**Slc46a1**	1,51701525	0,0000000	0,00000000
**Veph1**	1,51899545	0,0001067	0,00002249
**Bach2**	1,51909674	0,0000000	0,00000000
**Gm37305**	1,52009733	0,0000000	0,00000000
**Rhd**	1,52056485	0,0000000	0,00000000
**Gm5454**	1,52058927	0,0000000	0,00000000
**Mylpf**	1,5207133	0,0000002	0,00000003
**Arhgef9**	1,52113875	0,0000001	0,00000001
**Fbxo32**	1,52148749	0,0000000	0,00000000
**Pla2g2e**	1,52286946	0,0000000	0,00000000
**Itpr3**	1,52381701	0,0000000	0,00000000
**Ptms**	1,52600976	0,0000000	0,00000000

**C1ra**	1,5289543	0,0000000	0,00000000
**Gsto1**	1,52973323	0,0000000	0,00000000
**Gm5456**	1,53018706	0,0000430	0,00000851
**Eps8l1**	1,53138275	0,0000000	0,00000000
**Nudt7**	1,53418817	0,0000000	0,00000000
**Nrep**	1,53465168	0,0000000	0,00000000
**Etv1**	1,53473564	0,0000000	0,00000000
**Mr1**	1,53513072	0,0000000	0,00000000
**Slc1a4**	1,53614067	0,0000000	0,00000000
**Sema6d**	1,53705565	0,0000000	0,00000000
**Olfml3**	1,5378737	0,0000000	0,00000000
**9330102E08Rik**	1,54148977	0,0000000	0,00000000
**Gm38158**	1,54457839	0,0000000	0,00000000
**Dcdc2a**	1,54889552	0,0001694	0,00003691
**Gsn**	1,55107493	0,0000000	0,00000000
**Tcta**	1,55151922	0,0000000	0,00000000
**Tmem86a**	1,55373029	0,0000000	0,00000000
**Prdx5**	1,55561259	0,0000000	0,00000000
**Colgalt2**	1,55715358	0,0001600	0,00003469
**Slc39a13**	1,55755465	0,0000000	0,00000000
**Nipal4**	1,55797107	0,0000000	0,00000000
**Ptprd**	1,56174733	0,0005124	0,00012281
**Tlcd3b**	1,56177206	0,0016793	0,00044334
**4930402H24Rik**	1,56246207	0,0000000	0,00000000
**H2-T22**	1,56256573	0,0000000	0,00000000
**Maged2**	1,56388893	0,0000000	0,00000000
**Lncenc1**	1,56495595	0,0000038	0,00000064
**Pam**	1,5654993	0,0000000	0,00000000
**2010001A14Rik**	1,56560657	0,0002144	0,00004758
**Naip2**	1,5661432	0,0000000	0,00000000
**Sync**	1,56649311	0,0010849	0,00027616
**Gja1**	1,56772175	0,0000000	0,00000000
**B230217C12Rik**	1,57004894	0,0000000	0,00000000
**Gm2115**	1,57238397	0,0001837	0,00004023
**Olfm1**	1,57266769	0,0000000	0,00000000
**C330011M18Rik**	1,57506244	0,0009058	0,00022718
**Exoc3l**	1,57689814	0,0000462	0,00000919
**Lama5**	1,57806185	0,0000000	0,00000000
**Zfp697**	1,58149087	0,0000000	0,00000000
**Asap3**	1,58161356	0,0010524	0,00026739
**Fhl3**	1,5835488	0,0000000	0,00000000
**Mgat3**	1,58454359	0,0017717	0,00047022

**Dhh**	1,58538942	0,0003770	0,00008802
**Kcnu1**	1,58637001	0,0000793	0,00001643
**Pls3**	1,58683768	0,0000000	0,00000000
**Klf7**	1,58684018	0,0000000	0,00000000
**Drc3**	1,5903551	0,0000019	0,00000030
**Scube3**	1,5908861	0,0000000	0,00000000
**S100a10**	1,59581152	0,0000000	0,00000000
**Adcy5**	1,5964771	0,0000318	0,00000614
**Gm4285**	1,59688224	0,0000592	0,00001200
**Eme2**	1,59693481	0,0000000	0,00000000
**Cyp4f13**	1,59793008	0,0000000	0,00000000
**Gm17435**	1,60226433	0,0000005	0,00000007
**Cercam**	1,6037173	0,0000004	0,00000006
**Phyh**	1,60409593	0,0000000	0,00000000
**Tent5a**	1,6050494	0,0000000	0,00000000
**Epb41l1**	1,6059993	0,0000000	0,00000000
**Rbm11**	1,60676309	0,0008458	0,00021148
**Gm19569**	1,60731085	0,0001894	0,00004160
**Otos**	1,60821988	0,0000072	0,00000127
**F8**	1,60885765	0,0000000	0,00000000
**Rnd3**	1,60902584	0,0000000	0,00000000
**Col7a1**	1,60924785	0,0000000	0,00000000
**Traf5**	1,60946271	0,0006471	0,00015788
**Runx2**	1,60966022	0,0000000	0,00000000
**Mllt11**	1,61273235	0,0000000	0,00000000
**Rep15**	1,6145789	0,0000029	0,00000048
**Tmem190**	1,61479133	0,0005064	0,00012122
**Sphk1**	1,61489163	0,0000000	0,00000000
**Slc17a7**	1,61502544	0,0000562	0,00001135
**Procr**	1,61510122	0,0000000	0,00000000
**Was**	1,61534616	0,0002816	0,00006408
**Tgfb1**	1,61603368	0,0000000	0,00000000
**Scrn1**	1,61966241	0,0000000	0,00000000
**Prrg2**	1,62114571	0,0000000	0,00000000
**Il4ra**	1,62148586	0,0000529	0,00001065
**Slc2a13**	1,62446179	0,0000694	0,00001424
**Slc43a2**	1,63319219	0,0000000	0,00000000
**Nat8f1**	1,6339804	0,0000137	0,00000251
**H2-Eb1**	1,63418453	0,0017306	0,00045805
**Chrm1**	1,63456749	0,0013816	0,00035905
**Psd3**	1,63740347	0,0000001	0,00000001
**Etfrf1**	1,63808298	0,0000000	0,00000000

**Il1rap**	1,63860141	0,0000000	0,00000000
**Elovl7**	1,642172	0,0000000	0,00000000
**Nnmt**	1,64374643	0,0000000	0,00000000
**Eva1a**	1,64641174	0,0000000	0,00000000
**Abhd4**	1,64834561	0,0000000	0,00000000
**Hdac7**	1,65087622	0,0000000	0,00000000
**9330104G04Rik**	1,65207711	0,0000016	0,00000026
**Ahrr**	1,65231131	0,0000000	0,00000000
**Ypel2**	1,65256007	0,0000000	0,00000000
**Ccng1**	1,65260266	0,0000000	0,00000000
**Hkdc1**	1,65505735	0,0002449	0,00005490
**Foxr2**	1,65578968	0,0000000	0,00000000
**Dop1b**	1,65653544	0,0000000	0,00000000
**Gadd45b**	1,65656423	0,0000000	0,00000000
**Irs2**	1,65832447	0,0000000	0,00000000
**Tcn2**	1,65891315	0,0000000	0,00000000
**C030014I23Rik**	1,6614541	0,0000043	0,00000073
**Rasl11a**	1,66319844	0,0000000	0,00000000
**Mgst1**	1,66502574	0,0000000	0,00000000
**Kcnn4**	1,66551036	0,0003693	0,00008608
**Bcl2l1**	1,66706534	0,0000000	0,00000000
**Adcy1**	1,66782497	0,0016872	0,00044559
**Fbxo6**	1,66856857	0,0000000	0,00000000
**Ampd3**	1,66881518	0,0000000	0,00000000
**Aldh3b1**	1,66918052	0,0000000	0,00000000
**Gm20125**	1,67158568	0,0016741	0,00044190
**Nebl**	1,67196876	0,0000000	0,00000000
**Myl6b**	1,67328703	0,0000000	0,00000000
**Mylip**	1,67437848	0,0000000	0,00000000
**1700096K18Rik**	1,67743156	0,0002480	0,00005569
**Hmox1**	1,67922639	0,0000000	0,00000000
**Fam214a**	1,68061285	0,0000000	0,00000000
**Map1lc3a**	1,68131841	0,0000000	0,00000000
**Rgs2**	1,68154569	0,0000000	0,00000000
**Epha7**	1,68374879	0,0000000	0,00000000
**H2-DMa**	1,68389668	0,0002945	0,00006734
**Kazald1**	1,6844781	0,0005889	0,00014282
**1110025M09Rik**	1,68604108	0,0002475	0,00005555
**Alyref2**	1,69036912	0,0002048	0,00004524
**Eef1a2**	1,69266862	0,0001490	0,00003214
**Gm16124**	1,69667766	0,0001204	0,00002561
**Ssc5d**	1,69711477	0,0000000	0,00000000

**Fgfrl1**	1,70001107	0,0000000	0,00000000
**3930402G23Rik**	1,70110365	0,0000090	0,00000160
**Arap3**	1,7018172	0,0000000	0,00000000
**Cebpb**	1,70235908	0,0000000	0,00000000
**Ifi27l2a**	1,70367892	0,0013697	0,00035536
**Esd**	1,70794999	0,0000000	0,00000000
**Parp11**	1,70834871	0,0000000	0,00000000
**Cartpt**	1,70863394	0,0014432	0,00037625
**Cd59a**	1,70974142	0,0000000	0,00000000
**Platr22**	1,70976361	0,0016989	0,00044902
**Mme**	1,7106419	0,0000000	0,00000000
**Abcg2**	1,71148117	0,0000000	0,00000000
**Fam71e1**	1,71223957	0,0000352	0,00000686
**Lyz2**	1,7184312	0,0016158	0,00042544
**Junb**	1,72111065	0,0000000	0,00000000
**Flot1**	1,72248586	0,0000000	0,00000000
**Gucy1a1**	1,7239646	0,0003536	0,00008205
**Ccdc28a**	1,7246903	0,0000000	0,00000000
**Cadm4**	1,72637005	0,0000000	0,00000000
**Emp2**	1,72771884	0,0013148	0,00034030
**Syne3**	1,72937978	0,0000000	0,00000000
**Dtx4**	1,73186575	0,0000035	0,00000058
**Ggta1**	1,73412185	0,0000004	0,00000006
**Capn5**	1,73427932	0,0000000	0,00000000
**Atf5**	1,73515856	0,0000000	0,00000000
**Sparc**	1,736879	0,0000000	0,00000000
**Vwa5a**	1,73882561	0,0000000	0,00000000
**Kdm7a**	1,73972455	0,0000000	0,00000000
**Kdm5b**	1,74146198	0,0000000	0,00000000
**Lpar6**	1,74270562	0,0000000	0,00000000
**Bmp8a**	1,74350689	0,0003951	0,00009270
**Magi3**	1,74562411	0,0000000	0,00000000
**Thsd4**	1,74980477	0,0011100	0,00028308
**Yipf2**	1,75138048	0,0000000	0,00000000
**Col4a1**	1,75315805	0,0000000	0,00000000
**BC043934**	1,75461677	0,0000000	0,00000000
**Gm11696**	1,75615523	0,0000005	0,00000008
**Acad12**	1,75648524	0,0000000	0,00000000
**Crim1**	1,75809038	0,0000000	0,00000000
**Inka2**	1,7603129	0,0001708	0,00003723
**Nobox**	1,76377122	0,0010901	0,00027759
**Serinc3**	1,7664747	0,0000000	0,00000000

**Fosb**	1,76661178	0,0000008	0,00000013
**Hsd3b1**	1,76663538	0,0011243	0,00028705
**Hspb2**	1,76678803	0,0000000	0,00000000
**Hmga2**	1,76922655	0,0000000	0,00000000
**C920009B18Rik**	1,77067293	0,0000000	0,00000000
**Tinagl1**	1,77192134	0,0000317	0,00000612
**Slc11a1**	1,77503182	0,0000022	0,00000037
**Entpd2**	1,77588151	0,0004959	0,00011846
**Gstm7**	1,77643594	0,0000000	0,00000000
**Klhdc8b**	1,77691855	0,0000000	0,00000000
**Trp53cor1**	1,77709168	0,0000000	0,00000000
**Tsc22d1**	1,77819942	0,0000000	0,00000000
**Hmgcll1**	1,77832417	0,0010772	0,00027414
**Gm42901**	1,77832417	0,0010772	0,00027414
**Phldb2**	1,77926288	0,0000000	0,00000000
**Jph2**	1,77932531	0,0013181	0,00034125
**Gm37397**	1,78057093	0,0002183	0,00004851
**Gm12138**	1,78420597	0,0000000	0,00000000
**Itga2**	1,78452243	0,0000140	0,00000256
**Serpinb8**	1,78455097	0,0000012	0,00000019
**Stom**	1,78456064	0,0000000	0,00000000
**9130023H24Rik**	1,78475285	0,0000000	0,00000000
**Tulp1**	1,78539749	0,0004591	0,00010904
**Gm15850**	1,78651546	0,0001219	0,00002597
**Zfp36**	1,78719898	0,0000000	0,00000000
**Mapre3**	1,78903365	0,0000000	0,00000000
**Cst6**	1,79094597	0,0000000	0,00000000
**Fam161a**	1,79138602	0,0003532	0,00008195
**Ssc4d**	1,79563411	0,0000000	0,00000000
**Tiam2**	1,79690216	0,0000000	0,00000000
**H2-M10.5-ps1**	1,7972992	0,0009862	0,00024914
**Cracdl**	1,79731574	0,0007225	0,00017846
**Ephx1**	1,79734027	0,0000000	0,00000000
**Atf6**	1,79749122	0,0000000	0,00000000
**Plat**	1,7995395	0,0000000	0,00000000
**Cdk14**	1,80075436	0,0000000	0,00000000
**Angpt2**	1,8024121	0,0000000	0,00000000
**A930033H14Rik**	1,80241974	0,0000804	0,00001670
**Rhpn2**	1,80273983	0,0000201	0,00000376
**Rgs16**	1,8027542	0,0000000	0,00000000
**Tlr3**	1,80362114	0,0000000	0,00000000
**Tor4a**	1,80403014	0,0000000	0,00000000

**Ces2g**	1,80436469	0,0009648	0,00024328
**Cib4**	1,80436469	0,0009648	0,00024328
**Gm11638**	1,80799258	0,0009423	0,00023738
**Rtl5**	1,80816918	0,0000276	0,00000528
**Maf**	1,8101436	0,0000000	0,00000000
**Adam8**	1,81027859	0,0000012	0,00000020
**Tmeff2**	1,81036626	0,0004041	0,00009494
**Mpv17l**	1,81104518	0,0000000	0,00000000
**Pinlyp**	1,81146711	0,0009363	0,00023559
**Duox2**	1,81146711	0,0009363	0,00023559
**Gm7936**	1,81260942	0,0000004	0,00000006
**Bcl6**	1,81311236	0,0000000	0,00000000
**Aldh1l2**	1,81355938	0,0000000	0,00000000
**Trim17**	1,81475727	0,0000053	0,00000091
**Coro2a**	1,8150761	0,0006975	0,00017149
**Fgfr1**	1,81574926	0,0000000	0,00000000
**Ankrd42**	1,81632815	0,0000000	0,00000000
**Dgat2**	1,81963359	0,0000000	0,00000000
**Fam43a**	1,82058114	0,0004656	0,00011073
**D330050G23Rik**	1,82075481	0,0000242	0,00000460
**Plk3**	1,82573953	0,0000000	0,00000000
**Olfr56**	1,82587557	0,0003694	0,00008612
**Nectin4**	1,82619827	0,0009059	0,00022726
**Map3k9**	1,82702462	0,0000003	0,00000004
**Adam23**	1,82710601	0,0000000	0,00000000
**Fcgrt**	1,83058612	0,0000000	0,00000000
**Pcolce2**	1,83059	0,0000619	0,00001258
**Tymp**	1,83425241	0,0000000	0,00000001
**Vmac**	1,83720084	0,0000000	0,00000000
**Ctsh**	1,83749117	0,0000000	0,00000000
**Bmp1**	1,83819218	0,0000000	0,00000000
**Apol10b**	1,83912496	0,0008236	0,00020545
**Gm11639**	1,83932529	0,0007398	0,00018309
**Guca1a**	1,83975697	0,0000000	0,00000000
**Tec**	1,84053456	0,0000000	0,00000000
**4930447F24Rik**	1,84249265	0,0000563	0,00001137
**Gch1**	1,84314715	0,0000001	0,00000001
**B4galnt3**	1,84339828	0,0017456	0,00046228
**Bcam**	1,84916211	0,0000000	0,00000000
**Gm11490**	1,85010903	0,0014018	0,00036458
**Jup**	1,85125263	0,0000000	0,00000000
**Plin2**	1,85145833	0,0000000	0,00000000

**E230013L22Rik**	1,85227842	0,0007694	0,00019111
**Camk2b**	1,85354633	0,0000000	0,00000000
**Ano1**	1,85477798	0,0000000	0,00000000
**8030451A03Rik**	1,8553003	0,0007099	0,00017509
**Ptk7**	1,85824174	0,0000002	0,00000003
**Prss56**	1,85915058	0,0013181	0,00034127
**Chac1**	1,85993682	0,0000000	0,00000000
**Cd1d1**	1,86151733	0,0001281	0,00002739
**Rbpms2**	1,86451574	0,0000000	0,00000000
**Fam117a**	1,86819294	0,0000011	0,00000018
**Efnb2**	1,86927782	0,0000000	0,00000000
**Rimbp3**	1,86967327	0,0000003	0,00000004
**Tmem229b**	1,8705056	0,0000000	0,00000000
**Egr2**	1,87161346	0,0016284	0,00042906
**Serpinf1**	1,8746418	0,0000000	0,00000000
**Atp10b**	1,87532343	0,0000001	0,00000001
**Wdr45**	1,87546689	0,0000000	0,00000000
**Gm15753**	1,87773884	0,0007098	0,00017501
**Hrh1**	1,87824964	0,0007122	0,00017568
**Glis3**	1,87892372	0,0000000	0,00000000
**Isoc2b**	1,8791344	0,0000000	0,00000000
**Abca8b**	1,87919949	0,0000000	0,00000000
**Tgif1**	1,87977824	0,0000000	0,00000000
**Olig3**	1,88047657	0,0006347	0,00015467
**Col12a1**	1,88236857	0,0000000	0,00000000
**P2rx3**	1,88399028	0,0005128	0,00012291
**Tnfrsf12a**	1,88546585	0,0000000	0,00000000
**Grik3**	1,88562646	0,0000000	0,00000000
**Gm20005**	1,88571268	0,0000077	0,00000136
**Hid1**	1,88670053	0,0000000	0,00000000
**Cd68**	1,88804298	0,0000000	0,00000000
**Slc40a1**	1,88887907	0,0000000	0,00000000
**Steap1**	1,88963751	0,0000000	0,00000000
**Gsta5**	1,89173038	0,0002601	0,00005872
**Gm12854**	1,89483469	0,0000000	0,00000000
**Rln1**	1,8975674	0,0011920	0,00030588
**Trim47**	1,89767906	0,0000000	0,00000000
**Scrt1**	1,89861362	0,0006384	0,00015563
**Pde7b**	1,90109842	0,0000000	0,00000000
**Tmem102**	1,9026744	0,0013788	0,00035826
**Gm14808**	1,90352349	0,0000005	0,00000007
**Hsd11b1**	1,90384465	0,0000000	0,00000000

**Psme2**	1,90490207	0,0000000	0,00000000
**Flnc**	1,90580271	0,0000136	0,00000248
**Bpifa2**	1,90604347	0,0006347	0,00015465
**Mettl27**	1,90613673	0,0000000	0,00000000
**Pcdhb3**	1,90718722	0,0006416	0,00015651
**Igsf23**	1,9098204	0,0000013	0,00000021
**Gm15704**	1,91102407	0,0006027	0,00014651
**Papln**	1,91177241	0,0000000	0,00000000
**Sec16b**	1,91407385	0,0000000	0,00000000
**Tnfrsf1b**	1,9142587	0,0000437	0,00000865
**Aldh6a1**	1,91618073	0,0000000	0,00000000
**Shisa5**	1,9187639	0,0000000	0,00000000
**Bpgm**	1,9197028	0,0000000	0,00000000
**Glrx**	1,92253767	0,0000000	0,00000000
**Iglon5**	1,92376644	0,0000045	0,00000078
**Nedd9**	1,92501887	0,0000339	0,00000658
**Bcar3**	1,92614922	0,0000000	0,00000000
**Esyt1**	1,9292159	0,0000000	0,00000000
**Pcdhb16**	1,93080899	0,0000000	0,00000000
**Gm5617**	1,93452711	0,0000000	0,00000000
**2810402E24Rik**	1,93548992	0,0000000	0,00000000
**Dmkn**	1,93850714	0,0005485	0,00013231
**Zswim4**	1,93975303	0,0000000	0,00000000
**Mmp10**	1,94173685	0,0005450	0,00013139
**Trim66**	1,94804319	0,0008406	0,00021007
**Neurl1a**	1,9489109	0,0000000	0,00000000
**F11r**	1,9522613	0,0000000	0,00000000
**Klhl26**	1,95270656	0,0000000	0,00000000
**Gm37855**	1,95608957	0,0000000	0,00000000
**Styxl1**	1,95907017	0,0007961	0,00019826
**Ano2**	1,95911509	0,0001346	0,00002884
**Pon3**	1,95959241	0,0000000	0,00000000
**Tmem119**	1,96095641	0,0005586	0,00013489
**Ell2**	1,96118679	0,0000000	0,00000000
**Apoa2**	1,96170543	0,0000042	0,00000071
**Galr2**	1,9618053	0,0000000	0,00000000
**Ypel3**	1,96242286	0,0000000	0,00000000
**Gsap**	1,9629209	0,0011934	0,00030631
**Cstb**	1,96399738	0,0000000	0,00000000
**Prune2**	1,96476946	0,0000000	0,00000000
**Gcnt2**	1,96547323	0,0000000	0,00000000
**Pnck**	1,9664037	0,0000000	0,00000000

**Tnfsf10**	1,9675809	0,0004845	0,00011564
**Dusp5**	1,96813737	0,0000007	0,00000012
**Gsta2**	1,96898258	0,0000000	0,00000000
**Pcdhb7**	1,96901388	0,0000610	0,00001240
**Ifit1bl1**	1,97026089	0,0007538	0,00018685
**Gm45713**	1,97078664	0,0001461	0,00003148
**Ppm1j**	1,97177992	0,0005878	0,00014252
**Irs1**	1,97569574	0,0000000	0,00000000
**Gm37170**	1,97581134	0,0000014	0,00000022
**1700057H21Rik**	1,97584518	0,0003818	0,00008929
**Serinc5**	1,97622882	0,0000000	0,00000000
**Gm13110**	1,976444	0,0000000	0,00000000
**Ptprn**	1,97688718	0,0000629	0,00001281
**4930539M17Rik**	1,97713076	0,0007416	0,00018361
**Arid5b**	1,97716767	0,0000000	0,00000000
**Aldh1l1**	1,98453018	0,0000000	0,00000000
**Mthfd2l**	1,98483144	0,0000000	0,00000000
**Cpt1c**	1,98485155	0,0000000	0,00000000
**S100a11**	1,9853842	0,0000000	0,00000000
**Fbxw9**	1,98721218	0,0000000	0,00000000
**Odf3l1**	1,98812252	0,0000000	0,00000000
**Dio2**	1,98816233	0,0000931	0,00001948
**Herc3**	1,98820461	0,0000000	0,00000000
**Atoh8**	1,98827322	0,0000000	0,00000000
**Ilk**	1,98985433	0,0010244	0,00025984
**Adamts7**	1,99333356	0,0000076	0,00000135
**Mcub**	1,993689	0,0001488	0,00003209
**Gm8860**	1,99684351	0,0004062	0,00009545
**Adamts4**	1,99734027	0,0000000	0,00000000
**Syt11**	1,99820372	0,0000000	0,00000000
**Cxcl1**	1,99988561	0,0002125	0,00004708
**Wnt3**	2,00016036	0,0004161	0,00009789
**Adssl1**	2,00016828	0,0000000	0,00000000
**Fahd1**	2,00343172	0,0000000	0,00000000
**Gm12187**	2,0041593	0,0004239	0,00009994
**Gm7665**	2,00519248	0,0000000	0,00000000
**4930533K18Rik**	2,00647074	0,0016682	0,00044010
**Gm10602**	2,00895419	0,0000000	0,00000000
**Gm10461**	2,01118049	0,0000004	0,00000006
**Tnfrsf26**	2,01150644	0,0007140	0,00017617
**Gm15663**	2,01214641	0,0002365	0,00005292
**Fam13c**	2,0155199	0,0000000	0,00000000

**Rfpl4b**	2,01600684	0,0003537	0,00008210
**Serpini1**	2,01760834	0,0000000	0,00000000
**Pacrg**	2,0187318	0,0002259	0,00005029
**Trim56**	2,02042824	0,0000000	0,00000000
**Gm4755**	2,02261847	0,0000180	0,00000335
**Xkr6**	2,02322632	0,0003816	0,00008923
**Mest**	2,02380142	0,0000000	0,00000000
**Gm42815**	2,0266634	0,0000518	0,00001040
**Card10**	2,0285316	0,0000000	0,00000000
**Spred3**	2,02963046	0,0000000	0,00000000
**Dhrs9**	2,03219364	0,0003555	0,00008255
**Slit2**	2,03258296	0,0000000	0,00000000
**Hes1**	2,03278391	0,0000000	0,00000000
**Gmpr**	2,03545798	0,0000000	0,00000000
**Def6**	2,03702045	0,0000000	0,00000000
**Sema3c**	2,03832071	0,0000000	0,00000000
**9230114K14Rik**	2,04068389	0,0000000	0,00000000
**Rom1**	2,04276053	0,0000000	0,00000000
**Lipc**	2,04449625	0,0001837	0,00004022
**Syt8**	2,0449586	0,0003521	0,00008165
**Zscan4d**	2,0449586	0,0003521	0,00008165
**Stbd1**	2,04572378	0,0009877	0,00024963
**Sag**	2,04588442	0,0000000	0,00000000
**Map2k3os**	2,04629527	0,0000000	0,00000000
**Lgals9**	2,04962034	0,0000000	0,00000000
**Rfx4**	2,04980634	0,0001766	0,00003858
**Tspyl4**	2,05102637	0,0000000	0,00000000
**Gm28818**	2,05337773	0,0003733	0,00008713
**Gm26651**	2,06007064	0,0014401	0,00037530
**Oit3**	2,06354879	0,0013377	0,00034675
**Ctso**	2,06367315	0,0000000	0,00000000
**Gm10575**	2,06936458	0,0000124	0,00000226
**Scn2a**	2,06999246	0,0000000	0,00000000
**Mical2**	2,07262741	0,0000000	0,00000000
**Lcp1**	2,07292579	0,0000000	0,00000000
**Rsph1**	2,07760011	0,0000001	0,00000002
**Socs1**	2,07810555	0,0000000	0,00000000
**Slc5a3**	2,07888438	0,0000000	0,00000000
**Hrc**	2,08457762	0,0000350	0,00000682
**Pcdhb15**	2,08516175	0,0000000	0,00000000
**Rnd1**	2,08551189	0,0000000	0,00000000
**Sarm1**	2,08685122	0,0000000	0,00000000

**Dnmt3l**	2,08739353	0,0002702	0,00006128
**Gm11216**	2,08939294	0,0002975	0,00006809
**Gadd45a**	2,08967923	0,0000000	0,00000000
**Phkg1**	2,09002453	0,0000000	0,00000000
**Capg**	2,09008989	0,0000000	0,00000000
**Cebpd**	2,09060971	0,0000000	0,00000000
**Alpk2**	2,09362845	0,0002930	0,00006692
**Tmem92**	2,09534689	0,0002833	0,00006455
**Tcaf2**	2,09563774	0,0000000	0,00000000
**Thrb**	2,09831899	0,0001797	0,00003931
**Gm15910**	2,10338166	0,0000002	0,00000003
**Dusp14**	2,10367058	0,0000000	0,00000000
**Lrtm2**	2,10602296	0,0000760	0,00001570
**Ttll7**	2,1103024	0,0000000	0,00000000
**Hsd17b7**	2,11371665	0,0000000	0,00000000
**Irak2**	2,11687397	0,0000000	0,00000000
**Reps2**	2,11803389	0,0002589	0,00005841
**Fut4**	2,11979007	0,0001258	0,00002684
**Bgn**	2,11983487	0,0002632	0,00005950
**4930447N08Rik**	2,12322547	0,0004497	0,00010662
**Ddah1**	2,12335379	0,0000000	0,00000000
**Cma1**	2,12485059	0,0002459	0,00005516
**Cstad**	2,12529125	0,0001788	0,00003910
**Slc6a9**	2,13080142	0,0000000	0,00000000
**Gm32591**	2,13169143	0,0000004	0,00000006
**Gstm6**	2,13252855	0,0000000	0,00000000
**Lhx6**	2,13625418	0,0000099	0,00000177
**Upp1**	2,13768138	0,0000192	0,00000358
**Svop**	2,13905672	0,0002081	0,00004603
**Anxa2**	2,14228759	0,0000000	0,00000000
**Loxl4**	2,14260495	0,0000000	0,00000000
**Col4a5**	2,14405128	0,0000000	0,00000000
**Mucl3**	2,14636055	0,0000072	0,00000128
**1700003M07Rik**	2,14669956	0,0009877	0,00024957
**Psme2b**	2,14745906	0,0000000	0,00000000
**Uox**	2,14881575	0,0000566	0,00001142
**AW011738**	2,15039036	0,0000000	0,00000000
**Pcdhb11**	2,15094262	0,0000000	0,00000000
**Rcan2**	2,15249727	0,0002540	0,00005721
**Ccdc96**	2,15355732	0,0000000	0,00000000
**Siglecg**	2,15570246	0,0001993	0,00004395
**AI839979**	2,16271317	0,0000000	0,00000000

**Piwil4**	2,16448265	0,0002130	0,00004721
**Mvp**	2,16606531	0,0000000	0,00000000
**Tek**	2,17030707	0,0002102	0,00004652
**Dcxr**	2,1713572	0,0000000	0,00000000
**Tmem217**	2,17645526	0,0002307	0,00005146
**Zscan4c**	2,17725467	0,0008490	0,00021232
**S100a8**	2,17729609	0,0002071	0,00004579
**Pcdhb9**	2,17778997	0,0000430	0,00000850
**Defb25**	2,17817578	0,0000346	0,00000673
**Nol4**	2,18076595	0,0000000	0,00000000
**Tcam1**	2,18288798	0,0000226	0,00000427
**Cpne5**	2,18367303	0,0008212	0,00020475
**Cngb1**	2,18769198	0,0000000	0,00000000
**Cnga3**	2,19527806	0,0001887	0,00004140
**Fgf1**	2,19753693	0,0000000	0,00000000
**Tmem202**	2,19808165	0,0000000	0,00000000
**Gm43549**	2,19972088	0,0000000	0,00000000
**Pcp4l1**	2,20001444	0,0001867	0,00004092
**Pcdh9**	2,20059174	0,0000000	0,00000000
**Cyp3a16**	2,20363474	0,0000610	0,00001239
**Ddx4**	2,20387588	0,0000570	0,00001151
**2210010C04Rik**	2,20534051	0,0000017	0,00000027
**Ccn4**	2,20706011	0,0000000	0,00000000
**Sgk1**	2,20852808	0,0000000	0,00000000
**Fam214b**	2,20878358	0,0000000	0,00000000
**Ank3**	2,21024568	0,0000000	0,00000000
**Sv2b**	2,21172112	0,0000000	0,00000000
**Tor3a**	2,21282093	0,0000000	0,00000000
**Ptges**	2,21455415	0,0002292	0,00005111
**Vcl**	2,21716023	0,0000000	0,00000000
**Pwwp3b**	2,21850728	0,0000000	0,00000000
**Gm26616**	2,21862569	0,0000000	0,00000000
**Psd2**	2,22672072	0,0000313	0,00000606
**Gm16201**	2,2274807	0,0000035	0,00000060
**Timp1**	2,22756959	0,0000000	0,00000000
**C130013H08Rik**	2,22875209	0,0000000	0,00000000
**Sult6b1**	2,22912923	0,0000000	0,00000000
**Tbc1d2**	2,22920759	0,0000000	0,00000000
**C130083M11Rik**	2,2298371	0,0000000	0,00000000
**Ccnd1**	2,23010944	0,0000000	0,00000000
**Vamp1**	2,23220055	0,0000000	0,00000000
**A230083G16Rik**	2,23566463	0,0000001	0,00000001

**Marchf4**	2,23656466	0,0001033	0,00002172
**Neurl3**	2,23758105	0,0000000	0,00000000
**Gm4524**	2,23993211	0,0000000	0,00000000
**Slc2a9**	2,24113486	0,0000000	0,00000000
**Pcdhb8**	2,24129195	0,0000274	0,00000525
**Sned1**	2,24212237	0,0000000	0,00000000
**Eif1ad8**	2,24264705	0,0001512	0,00003267
**Hao1**	2,24622011	0,0000335	0,00000649
**Septin4**	2,25063762	0,0000000	0,00000000
**9130008F23Rik**	2,25127411	0,0000000	0,00000000
**H1f11-ps**	2,2519974	0,0000670	0,00001370
**Gm16104**	2,25236631	0,0000019	0,00000031
**Elfn1**	2,25301964	0,0005351	0,00012871
**Ttll6**	2,25580668	0,0005303	0,00012751
**Pcdhb2**	2,25679869	0,0000005	0,00000007
**Pramel31**	2,25828356	0,0005760	0,00013939
**Rbms3**	2,25982379	0,0000003	0,00000005
**Gm12216**	2,26060296	0,0002933	0,00006699
**Dpf3**	2,26193769	0,0000093	0,00000167
**Gm5546**	2,26377497	0,0000000	0,00000000
**Fosl1**	2,2643095	0,0000002	0,00000002
**Gm20257**	2,26629238	0,0000000	0,00000000
**Adamts14**	2,26696854	0,0000000	0,00000000
**Rras**	2,27128516	0,0000000	0,00000000
**Camk1d**	2,27316768	0,0017545	0,00046522
**Lpin3**	2,27694556	0,0000000	0,00000000
**Btla**	2,27865836	0,0000682	0,00001398
**Styk1**	2,28110886	0,0001540	0,00003333
**Uchl1**	2,28162522	0,0000000	0,00000000
**Trim16**	2,28827934	0,0000000	0,00000000
**Samd5**	2,29029723	0,0000000	0,00000000
**Gngt2**	2,29399602	0,0016467	0,00043410
**Podn**	2,2985527	0,0001240	0,00002644
**Trp53inp1**	2,29952869	0,0000000	0,00000000
**Neb**	2,30253942	0,0000000	0,00000000
**Frat2**	2,30263781	0,0000000	0,00000001
**Slc39a4**	2,3027069	0,0000203	0,00000380
**Pqlc3**	2,30304807	0,0000000	0,00000000
**Spp1**	2,30329949	0,0000000	0,00000000
**Pdlim2**	2,30788529	0,0000000	0,00000000
**Gm13056**	2,30872343	0,0001191	0,00002534
**Msrb3**	2,31052511	0,0000000	0,00000000

**Igfbp6**	2,31451118	0,0000257	0,00000489
**Scnn1a**	2,31582891	0,0000513	0,00001030
**Gm5152**	2,32042464	0,0015140	0,00039622
**Tm4sf1**	2,32075809	0,0000000	0,00000000
**Pde4a**	2,32359642	0,0000000	0,00000000
**Gm44386**	2,32476375	0,0000000	0,00000000
**Igf2bp2**	2,32631439	0,0000000	0,00000000
**Pkp2**	2,32644063	0,0000000	0,00000000
**Ptprf**	2,327558	0,0000000	0,00000000
**Calcoco1**	2,3281609	0,0000000	0,00000000
**Usp17lc**	2,3308913	0,0001033	0,00002173
**Gm8818**	2,33096136	0,0000000	0,00000000
**Hacd4**	2,33281234	0,0000000	0,00000000
**2700046G09Rik**	2,33488312	0,0000000	0,00000000
**Shank1**	2,33789576	0,0000000	0,00000000
**Pdcd4**	2,33884759	0,0000000	0,00000000
**Ccdc189**	2,34435557	0,0000000	0,00000000
**9530034E10Rik**	2,34578897	0,0000946	0,00001980
**Trim21**	2,3458533	0,0000000	0,00000000
**Pcdh1**	2,34778451	0,0000000	0,00000000
**Tmem72**	2,350519	0,0013287	0,00034416
**Ypel5**	2,35125604	0,0000000	0,00000000
**Aldh1a1**	2,35520238	0,0000859	0,00001789
**0610009L18Rik**	2,35602919	0,0000001	0,00000001
**Gm44250**	2,35618539	0,0000000	0,00000000
**Spsb2**	2,36030929	0,0000000	0,00000000
**Ass1**	2,36203112	0,0000000	0,00000000
**Mov10**	2,36250337	0,0000000	0,00000000
**Gm26645**	2,36952137	0,0000007	0,00000010
**H2aw**	2,36993425	0,0000000	0,00000000
**H2-D1**	2,37279197	0,0000000	0,00000000
**Cd79a**	2,37420431	0,0000010	0,00000015
**Mt3**	2,38235657	0,0002876	0,00006562
**Greb1**	2,38253971	0,0000000	0,00000000
**Akp-ps1**	2,38411984	0,0000222	0,00000418
**Slc22a4**	2,38551055	0,0000008	0,00000012
**Gm5424**	2,39366779	0,0000000	0,00000000
**Fosl2**	2,39429414	0,0000000	0,00000000
**Pcdhb4**	2,39653651	0,0000000	0,00000000
**Cntn1**	2,39741339	0,0000000	0,00000000
**Cdkn1a**	2,39798428	0,0000000	0,00000000
**Zcchc24**	2,40355044	0,0000000	0,00000000

**Gpr35**	2,40719669	0,0000000	0,00000000
**Pyroxd2**	2,40816507	0,0000000	0,00000000
**Syn1**	2,41127012	0,0000000	0,00000000
**Gbe1**	2,41306017	0,0000000	0,00000000
**Gm3716**	2,41851831	0,0000000	0,00000000
**Gm10800**	2,42505843	0,0000005	0,00000007
**Jam2**	2,42881822	0,0000000	0,00000000
**Pparg**	2,42933711	0,0000017	0,00000028
**Gm12059**	2,42955359	0,0000000	0,00000000
**Tcp11l2**	2,4346037	0,0000000	0,00000000
**Tslp**	2,435082	0,0000000	0,00000000
**Gsdmd**	2,44330388	0,0000000	0,00000000
**Plekhh2**	2,44554727	0,0000000	0,00000000
**Txlnb**	2,44645181	0,0000008	0,00000013
**B2m**	2,4469379	0,0000000	0,00000000
**Tent5c**	2,44989681	0,0000000	0,00000000
**Mturn**	2,45056448	0,0000000	0,00000000
**Psmb9**	2,45139067	0,0000854	0,00001778
**P2ry6**	2,45157536	0,0000656	0,00001340
**Nptx1**	2,45305759	0,0000000	0,00000000
**Megf10**	2,45333225	0,0000000	0,00000000
**Faim2**	2,45739247	0,0009340	0,00023483
**Slc16a13**	2,45834762	0,0000000	0,00000000
**Gm28447**	2,46052278	0,0000001	0,00000002
**Robo2**	2,46185427	0,0000030	0,00000049
**Cyb5r2**	2,46214729	0,0000000	0,00000000
**Gm12602**	2,46745354	0,0000000	0,00000000
**Cnn2**	2,46806223	0,0000000	0,00000000
**Mxd4**	2,47131692	0,0000000	0,00000000
**Slco2a1**	2,47650098	0,0000001	0,00000001
**Gm11816**	2,47670132	0,0000591	0,00001199
**Gadd45g**	2,47840084	0,0000000	0,00000000
**Sp110**	2,48041845	0,0000716	0,00001471
**Ahnak**	2,48434457	0,0000000	0,00000000
**Jag1**	2,4851173	0,0000000	0,00000000
**Ccpg1os**	2,48547258	0,0000000	0,00000000
**Chst11**	2,48912392	0,0000000	0,00000000
**Dtx3l**	2,49527986	0,0000000	0,00000000
**Azin2**	2,49538698	0,0000000	0,00000000
**Glipr2**	2,4965854	0,0000000	0,00000000
**Gstm1**	2,49665809	0,0000000	0,00000000
**Iigp1**	2,50100238	0,0000506	0,00001015

**Bcl11b**	2,51035471	0,0000060	0,00000104
**Kcnk3**	2,51172301	0,0000463	0,00000922
**Cdk5r2**	2,51174822	0,0000000	0,00000000
**Sfxn3**	2,51378478	0,0000000	0,00000000
**Tmem171**	2,51381869	0,0000502	0,00001005
**Slc7a11**	2,5174001	0,0000000	0,00000000
**Gfra1**	2,51767466	0,0000000	0,00000000
**Arhgef19**	2,5196302	0,0000000	0,00000000
**Niban1**	2,52218077	0,0000000	0,00000000
**Hecw2**	2,52486749	0,0000000	0,00000000
**Trp53inp2**	2,52868497	0,0000000	0,00000000
**Sod3**	2,52898469	0,0000000	0,00000000
**Plcb2**	2,52965738	0,0000000	0,00000000
**Selenop**	2,53656024	0,0000000	0,00000000
**Ctsf**	2,53772462	0,0000000	0,00000000
**Gm11734**	2,53828163	0,0000000	0,00000000
**Gm9899**	2,53888394	0,0000453	0,00000900
**Rida**	2,54182349	0,0000000	0,00000000
**Usp17lb**	2,54246115	0,0007051	0,00017359
**Gstm2-ps1**	2,54293575	0,0000000	0,00000000
**Esr2**	2,54384285	0,0000401	0,00000790
**Adgra1**	2,54925011	0,0007068	0,00017418
**Trim14**	2,55110084	0,0000000	0,00000000
**Sec14l5**	2,55146369	0,0006621	0,00016205
**Gpat3**	2,55364004	0,0000002	0,00000003
**Susd3**	2,55378288	0,0000000	0,00000000
**Arhgef28**	2,5538354	0,0000000	0,00000000
**Oas1c**	2,5572971	0,0000000	0,00000000
**Parp3**	2,55784076	0,0000000	0,00000000
**Synm**	2,55911913	0,0000000	0,00000000
**Mir22hg**	2,5637348	0,0000000	0,00000000
**Asb2**	2,56670867	0,0000000	0,00000000
**Pltp**	2,56677154	0,0000000	0,00000000
**Unc5b**	2,57177879	0,0000000	0,00000000
**Dusp10**	2,57407662	0,0000000	0,00000000
**Aox1**	2,57531556	0,0000000	0,00000000
**P2rx5**	2,57643921	0,0000000	0,00000000
**Cp**	2,58045777	0,0000000	0,00000000
**Cited4**	2,58234143	0,0000049	0,00000085
**Slc37a2**	2,58727233	0,0000000	0,00000000
**Mx1**	2,58753803	0,0000000	0,00000000
**Dnah5**	2,58943153	0,0006134	0,00014923

**Dusp27**	2,59287089	0,0000000	0,00000000
**Cd109**	2,59384553	0,0000000	0,00000000
**Gm10687**	2,59527434	0,0000000	0,00000000
**Chit1**	2,59819302	0,0000345	0,00000671
**Gldn**	2,59977848	0,0001028	0,00002162
**Slc16a5**	2,60331977	0,0000000	0,00000000
**Plch2**	2,60565892	0,0000001	0,00000001
**Cald1**	2,60871046	0,0000000	0,00000000
**Anxa1**	2,60921343	0,0000000	0,00000000
**Gm21818**	2,61368842	0,0000334	0,00000648
**Csf1**	2,61668261	0,0000000	0,00000000
**Hr**	2,61854075	0,0000000	0,00000000
**Sh3bgr**	2,62079418	0,0000000	0,00000000
**Mill2**	2,6211226	0,0000000	0,00000000
**Plaur**	2,62534089	0,0000000	0,00000000
**Xdh**	2,63196356	0,0005367	0,00012914
**2310043P16Rik**	2,63631219	0,0000000	0,00000000
**Adra1b**	2,63724654	0,0000682	0,00001398
**Ddit4l**	2,64555788	0,0000000	0,00000000
**Mrgprf**	2,65094652	0,0000277	0,00000532
**Steap2**	2,65297483	0,0000000	0,00000000
**Celf5**	2,66186335	0,0000006	0,00000010
**Mylk3**	2,66227799	0,0000001	0,00000001
**Stat2**	2,66470369	0,0000000	0,00000000
**Des**	2,67028133	0,0000000	0,00000000
**Mfge8**	2,67071057	0,0000000	0,00000000
**Upk3bl**	2,67203286	0,0000258	0,00000491
**Klf5**	2,67243112	0,0000000	0,00000000
**Pcdh17**	2,68087951	0,0000000	0,00000000
**Hspb6**	2,6816573	0,0000000	0,00000000
**Tgm2**	2,68423918	0,0000000	0,00000000
**Slc4a11**	2,68470435	0,0000000	0,00000000
**Eno3**	2,68617141	0,0000000	0,00000000
**Ccn1**	2,6867464	0,0000000	0,00000000
**Id1**	2,68820925	0,0000000	0,00000000
**Ndrg4**	2,69002336	0,0000000	0,00000000
**Cmpk2**	2,69016671	0,0000000	0,00000000
**Fxyd5**	2,69320162	0,0000000	0,00000000
**Serpinb6b**	2,69958146	0,0000000	0,00000000
**Col19a1**	2,70023823	0,0000512	0,00001027
**4732419C18Rik**	2,70534363	0,0000000	0,00000000
**Lctl**	2,71317874	0,0000000	0,00000000

**Adora2a**	2,7166581	0,0000000	0,00000000
**Col8a1**	2,71694374	0,0000216	0,00000406
**Ttll11**	2,7175328	0,0000000	0,00000000
**Arnt2**	2,71995518	0,0000000	0,00000000
**Prph**	2,72278366	0,0000000	0,00000000
**Lrrc75b**	2,72324145	0,0000000	0,00000000
**Fos**	2,7234784	0,0000000	0,00000000
**Scn1b**	2,726054	0,0000000	0,00000000
**Lgr6**	2,72770603	0,0000000	0,00000000
**Fat2**	2,73558174	0,0000199	0,00000371
**Irgm1**	2,74061177	0,0000000	0,00000000
**Pcdhb14**	2,74190372	0,0000000	0,00000000
**G730013B05Rik**	2,74597524	0,0000010	0,00000016
**Arl4d**	2,75102501	0,0000000	0,00000000
**Map1a**	2,75610389	0,0000000	0,00000000
**Vdr**	2,75645869	0,0000000	0,00000000
**Clca2**	2,76647098	0,0000171	0,00000317
**Rgs4**	2,77288923	0,0000135	0,00000245
**Ifi211**	2,774543	0,0000168	0,00000311
**Pdgfa**	2,78031101	0,0000000	0,00000000
**Frmd4b**	2,78428395	0,0000000	0,00000000
**Apobr**	2,78648747	0,0000000	0,00000000
**Rasa4**	2,79193483	0,0000000	0,00000000
**Pdk2**	2,79438907	0,0000000	0,00000000
**Mgst2**	2,79442181	0,0000001	0,00000001
**Mpeg1**	2,79486592	0,0000155	0,00000285
**Bmf**	2,79988967	0,0000000	0,00000000
**Pik3r5**	2,80364001	0,0000136	0,00000249
**Sucnr1**	2,80372169	0,0000011	0,00000017
**Rims1**	2,8080645	0,0000001	0,00000001
**Gm16010**	2,80839193	0,0000000	0,00000000
**Serpine2**	2,81054055	0,0000000	0,00000000
**Lhfp**	2,81469306	0,0000000	0,00000000
**Galnt18**	2,8258757	0,0000110	0,00000199
**Lgals3bp**	2,83611803	0,0000000	0,00000000
**Pla1a**	2,83627058	0,0002634	0,00005955
**Apod**	2,84094507	0,0000000	0,00000000
**Gm43197**	2,84115383	0,0000000	0,00000000
**Gm9887**	2,84580407	0,0000000	0,00000000
**Itga3**	2,84623325	0,0000000	0,00000000
**Mmp19**	2,85480223	0,0000001	0,00000001
**Myh3**	2,85611947	0,0000000	0,00000000

**Tfec**	2,85617318	0,0000000	0,00000000
**Col18a1**	2,86894139	0,0000000	0,00000000
**Fcgr4**	2,87398346	0,0000247	0,00000470
**Hrct1**	2,88003486	0,0000000	0,00000000
**Epha4**	2,88443935	0,0000000	0,00000000
**Myl9**	2,88675844	0,0000000	0,00000000
**Serpinb1b**	2,89143766	0,0000096	0,00000172
**Cd248**	2,89733329	0,0000000	0,00000000
**Pcdhb10**	2,89756587	0,0000000	0,00000000
**Pld2**	2,90666799	0,0000000	0,00000000
**Tmem106a**	2,91142346	0,0000000	0,00000000
**Pcyt1b**	2,91716487	0,0000000	0,00000000
**Aox4**	2,91739745	0,0000090	0,00000161
**Dmpk**	2,91766442	0,0000000	0,00000000
**Fbxo2**	2,92733254	0,0000000	0,00000000
**Dnm3**	2,92755848	0,0000000	0,00000000
**Rnf150**	2,92796854	0,0000000	0,00000000
**Ptgs1**	2,93196816	0,0000000	0,00000000
**6330403L08Rik**	2,93970008	0,0000000	0,00000000
**Smtnl1**	2,94932291	0,0000001	0,00000002
**Uba7**	2,95255961	0,0000000	0,00000000
**Lgals3**	2,95337322	0,0000000	0,00000000
**Parp8**	2,95626286	0,0000000	0,00000000
**Hfm1**	2,95637991	0,0000072	0,00000127
**Trim25**	2,95668963	0,0000000	0,00000000
**Folr1**	2,95762794	0,0000000	0,00000000
**Me3**	2,96061451	0,0000000	0,00000000
**Gm6556**	2,96163532	0,0000000	0,00000000
**Kctd11**	2,96237918	0,0000000	0,00000000
**Gm16325**	2,9660451	0,0000000	0,00000000
**Slc41a2**	2,96988352	0,0000000	0,00000000
**Gm15640**	2,97330382	0,0000000	0,00000000
**Plscr2**	2,97767826	0,0000000	0,00000000
**Nfam1**	2,98192788	0,0000060	0,00000104
**Kcnb1**	2,98401664	0,0000000	0,00000000
**Nmb**	2,99316162	0,0000000	0,00000000
**Gata3**	2,99972023	0,0000000	0,00000000
**Atp2a3**	3,00089568	0,0001105	0,00002336
**H2-T23**	3,00370311	0,0000000	0,00000000
**Thbs1**	3,01036692	0,0000000	0,00000000
**Slc22a23**	3,01817581	0,0000000	0,00000000
**Dchs2**	3,01866987	0,0000001	0,00000001

**Irf5**	3,02464555	0,0000000	0,00000000
**Cadm2**	3,02917133	0,0000000	0,00000000
**Grb10**	3,03416195	0,0001295	0,00002772
**Scn3b**	3,03452607	0,0000000	0,00000000
**Gm20559**	3,04057018	0,0000000	0,00000000
**Ccn3**	3,04323428	0,0000003	0,00000005
**Fn1**	3,05774849	0,0000000	0,00000000
**Myl1**	3,0589247	0,0000077	0,00000136
**Spef1l**	3,06813228	0,0000045	0,00000078
**Gm7893**	3,07217263	0,0000034	0,00000058
**Igdcc4**	3,0747407	0,0000000	0,00000000
**Gm17501**	3,07691121	0,0000000	0,00000000
**Tmem151a**	3,07881464	0,0000000	0,00000000
**Nppb**	3,08042347	0,0000000	0,00000000
**Slc27a1**	3,08262198	0,0000000	0,00000000
**Sema3e**	3,08922703	0,0000000	0,00000000
**S100a13**	3,09019088	0,0000000	0,00000000
**Kcnk2**	3,09898397	0,0000000	0,00000000
**Gm26797**	3,0994878	0,0000000	0,00000000
**Sqor**	3,10195861	0,0001040	0,00002189
**Psmb8**	3,11306855	0,0000039	0,00000066
**Ppef1**	3,12521966	0,0000937	0,00001961
**Acsbg1**	3,12928048	0,0000000	0,00000000
**Prr5l**	3,13030633	0,0000000	0,00000000
**2310008N11Rik**	3,13439544	0,0000035	0,00000060
**Akap12**	3,13518405	0,0000000	0,00000000
**Ptn**	3,13738006	0,0000920	0,00001925
**Postn**	3,14106776	0,0000000	0,00000000
**Irag1**	3,1415434	0,0000000	0,00000000
**Tnn**	3,1438506	0,0000032	0,00000053
**Lama3**	3,14716797	0,0000051	0,00000087
**Ace**	3,14960826	0,0000000	0,00000000
**Hmcn2**	3,16079393	0,0000000	0,00000000
**Gsta4**	3,16222915	0,0000000	0,00000000
**Prkag3**	3,16557483	0,0000030	0,00000050
**Edil3**	3,16708863	0,0000000	0,00000000
**Oasl1**	3,16774858	0,0000000	0,00000000
**1700007K13Rik**	3,16961543	0,0000000	0,00000000
**Acap1**	3,17139233	0,0000000	0,00000000
**Lamb1**	3,17334302	0,0000000	0,00000000
**Gm42765**	3,17641428	0,0000030	0,00000049
**Ttc9**	3,17751903	0,0000000	0,00000000

**Parp10**	3,18238426	0,0000000	0,00000000
**Ifi47**	3,18641291	0,0000000	0,00000000
**Slc17a8**	3,18870286	0,0000000	0,00000000
**Cpz**	3,18938315	0,0000000	0,00000000
**Tap2**	3,19531853	0,0000000	0,00000000
**Adcyap1r1**	3,20002892	0,0000017	0,00000028
**Samd9l**	3,20104391	0,0000000	0,00000000
**Tapbp**	3,21262175	0,0000000	0,00000000
**Sp9**	3,22000972	0,0000000	0,00000000
**Ube2l6**	3,22039102	0,0000000	0,00000000
**Tnfrsf22**	3,22608948	0,0000000	0,00000000
**Helz2**	3,22944615	0,0000000	0,00000000
**Lst1**	3,23074126	0,0000000	0,00000000
**Myof**	3,23154279	0,0000000	0,00000000
**Ier5l**	3,23234759	0,0000000	0,00000000
**Sgms2**	3,23738494	0,0000000	0,00000000
**4930417H01Rik**	3,23788151	0,0000023	0,00000037
**Tspan2**	3,24361907	0,0000000	0,00000000
**S100a7a**	3,24870893	0,0000017	0,00000027
**Grhl3**	3,24908847	0,0000000	0,00000000
**Nmnat2**	3,25242712	0,0000000	0,00000000
**0610012D04Rik**	3,2607123	0,0000000	0,00000000
**Bst2**	3,26199203	0,0000000	0,00000000
**Lgals7**	3,26748407	0,0000003	0,00000004
**Akap3**	3,27550562	0,0000024	0,00000039
**S100a3**	3,27602685	0,0000000	0,00000001
**Igf2**	3,2831933	0,0000014	0,00000022
**Nlrp10**	3,28878592	0,0000000	0,00000000
**Cdsn**	3,29376801	0,0000000	0,00000000
**Corin**	3,29698906	0,0000000	0,00000000
**Mdga1**	3,30003467	0,0000002	0,00000003
**Padi3**	3,31027046	0,0000002	0,00000002
**Ggt1**	3,31084894	0,0000000	0,00000000
**Rnase4**	3,31375567	0,0000000	0,00000000
**Rab11fip5**	3,31567627	0,0000000	0,00000000
**Nlrc3**	3,32307612	0,0000000	0,00000000
**Sertad4**	3,32531507	0,0000000	0,00000000
**Notch1**	3,33034803	0,0000000	0,00000000
**Irf9**	3,34330996	0,0000000	0,00000000
**Nlrc5**	3,35012438	0,0000000	0,00000000
**Serpina3n**	3,35235632	0,0000001	0,00000002
**Fblim1**	3,35400629	0,0000000	0,00000000

**Hcfc1r1**	3,36317482	0,0000000	0,00000000
**Artn**	3,36941709	0,0000000	0,00000000
**Lmod1**	3,37448008	0,0000000	0,00000000
**Oasl2**	3,37455711	0,0000000	0,00000000
**Pkhd1**	3,37586603	0,0000000	0,00000000
**Ltbp1**	3,37988218	0,0000000	0,00000000
**Usp17ld**	3,38871853	0,0000364	0,00000712
**Cav1**	3,39093826	0,0000000	0,00000000
**Kcnh2**	3,39353226	0,0000000	0,00000000
**Palld**	3,4015898	0,0000000	0,00000000
**Ccn2**	3,4068118	0,0000000	0,00000000
**Cd34**	3,4096655	0,0000000	0,00000000
**9330175E14Rik**	3,41366524	0,0000000	0,00000000
**Cd38**	3,42410259	0,0000000	0,00000000
**Robo3**	3,42821319	0,0000000	0,00000000
**Hspa1b**	3,42889793	0,0000000	0,00000000
**Apobec1**	3,43189033	0,0000000	0,00000000
**Atp1b2**	3,44205057	0,0000000	0,00000000
**Gsta3**	3,44521772	0,0000000	0,00000000
**C1s1**	3,4540066	0,0000000	0,00000000
**Slc16a11**	3,46297201	0,0000000	0,00000000
**Adh7**	3,4636076	0,0000000	0,00000000
**Galnt15**	3,46470117	0,0000007	0,00000011
**Plk2**	3,46974525	0,0000000	0,00000000
**Slfn8**	3,47423988	0,0000269	0,00000514
**Dhx58**	3,47719225	0,0000000	0,00000000
**Pramel19**	3,47847877	0,0000269	0,00000515
**Btg2**	3,47975584	0,0000000	0,00000000
**4930461G14Rik**	3,48274365	0,0000007	0,00000011
**Ak1**	3,48732787	0,0000000	0,00000000
**Trim34a**	3,4927983	0,0000000	0,00000000
**Tmprss11f**	3,50184159	0,0000000	0,00000000
**Pdgfb**	3,50539838	0,0000000	0,00000000
**Foxn1**	3,51201868	0,0000218	0,00000411
**Ccdc180**	3,51537322	0,0000006	0,00000009
**Zbp1**	3,52171611	0,0000005	0,00000008
**Serpinb1a**	3,53136119	0,0000000	0,00000000
**Lif**	3,53286167	0,0000000	0,00000000
**Il12rb1**	3,54547355	0,0000000	0,00000000
**Gm15767**	3,54571007	0,0000006	0,00000009
**Ldb3**	3,5474811	0,0000000	0,00000000
**Spn**	3,5505323	0,0000000	0,00000000

**Ppm1e**	3,55147322	0,0000000	0,00000000
**Nupr1**	3,5541424	0,0000000	0,00000000
**Rin1**	3,55563316	0,0000000	0,00000000
**Gm15228**	3,55670617	0,0000005	0,00000008
**Trim12a**	3,56163454	0,0000000	0,00000000
**Cst3**	3,56469586	0,0000000	0,00000000
**Nckap5**	3,57196083	0,0000000	0,00000000
**Gdpd2**	3,57284597	0,0000000	0,00000000
**Rnf213**	3,57737315	0,0000000	0,00000000
**Col10a1**	3,58000396	0,0000000	0,00000000
**Tnnt2**	3,58068119	0,0000000	0,00000000
**Efhd1**	3,59333022	0,0000000	0,00000000
**Usp17la**	3,61261257	0,0000004	0,00000006
**Tnxb**	3,61834307	0,0000000	0,00000000
**Ccdc9b**	3,62355495	0,0000000	0,00000000
**Nqo1**	3,63321988	0,0000000	0,00000000
**Gm2065**	3,63437788	0,0000000	0,00000000
**Ptpn5**	3,65695135	0,0000003	0,00000005
**Plekha6**	3,66769677	0,0000000	0,00000000
**Itga8**	3,67772269	0,0000000	0,00000000
**Nell2**	3,68820435	0,0000001	0,00000002
**Cobl**	3,69733617	0,0000003	0,00000004
**Ifit1bl2**	3,7428771	0,0000102	0,00000184
**Cfap70**	3,74966521	0,0000000	0,00000000
**Ifi35**	3,76451817	0,0000000	0,00000000
**Serpinb9**	3,77137219	0,0000000	0,00000000
**Parp12**	3,79995505	0,0000000	0,00000000
**Parp9**	3,8046578	0,0000000	0,00000000
**H2-K1**	3,81858347	0,0000000	0,00000000
**Lrig1**	3,82589172	0,0000000	0,00000000
**Tap1**	3,83030603	0,0000000	0,00000000
**Fbn1**	3,85114136	0,0000064	0,00000112
**Stat1**	3,85927604	0,0000000	0,00000000
**Prl2c5**	3,87047593	0,0000000	0,00000000
**Iqsec3**	3,87331414	0,0000060	0,00000105
**Tmem40**	3,87979958	0,0000000	0,00000000
**Prkg2**	3,88275792	0,0000000	0,00000000
**Wnt9a**	3,8900451	0,0000000	0,00000001
**Gpr176**	3,8923745	0,0000000	0,00000000
**Krt7**	3,90427006	0,0000054	0,00000094
**Ifi27**	3,91227526	0,0000000	0,00000000
**Timp4**	3,91948685	0,0000000	0,00000000

**Tgtp2**	3,92772311	0,0000001	0,00000001
**Fas**	3,94091531	0,0000001	0,00000001
**Egr1**	3,9528833	0,0000000	0,00000000
**Hspb1**	3,95617505	0,0000000	0,00000000
**Rsad2**	3,95763586	0,0000000	0,00000000
**Usp17le**	3,97058788	0,0000041	0,00000070
**Synpo2**	3,97235165	0,0000000	0,00000000
**Oas1b**	3,97275948	0,0000000	0,00000000
**Ndrg2**	3,97773921	0,0000000	0,00000000
**Actg2**	3,99734185	0,0000000	0,00000000
**Prl2c2**	4,00509822	0,0000000	0,00000000
**Kitl**	4,03355247	0,0000000	0,00000000
**Otog**	4,05628075	0,0000000	0,00000000
**Car5b**	4,06098315	0,0000000	0,00000000
**Ifih1**	4,06403621	0,0000000	0,00000000
**Pgf**	4,10457395	0,0000000	0,00000000
**Tmem179**	4,11046593	0,0000000	0,00000000
**Vgf**	4,13779807	0,0000000	0,00000000
**Klhdc8a**	4,14273464	0,0000000	0,00000000
**Plb1**	4,15780134	0,0000000	0,00000000
**Gm20658**	4,15908247	0,0000000	0,00000000
**Fbln2**	4,1713756	0,0000000	0,00000000
**Eda2r**	4,19516565	0,0000000	0,00000000
**Serping1**	4,20782289	0,0000000	0,00000000
**Chrnb1**	4,20843519	0,0000000	0,00000000
**Vat1l**	4,21854713	0,0000000	0,00000000
**Gm40038**	4,23431821	0,0000000	0,00000000
**Mx2**	4,24995465	0,0000000	0,00000000
**Axl**	4,30423295	0,0000000	0,00000000
**Slit1**	4,30747501	0,0000000	0,00000000
**Phf11d**	4,32081259	0,0000010	0,00000015
**Ifit3b**	4,3446813	0,0000000	0,00000000
**Sp100**	4,34905847	0,0000000	0,00000000
**Ecm1**	4,35611429	0,0000000	0,00000000
**Ifit3**	4,42112797	0,0000000	0,00000000
**Trim12c**	4,42254757	0,0000000	0,00000000
**Parp14**	4,43081677	0,0000000	0,00000000
**Trim30d**	4,44049241	0,0000006	0,00000009
**Marchf10**	4,45455384	0,0000000	0,00000000
**Ifi44**	4,45650693	0,0000005	0,00000008
**Gbp2**	4,45668972	0,0000000	0,00000000
**Oas1a**	4,46174809	0,0000000	0,00000000

**Cacna1a**	4,47916432	0,0000000	0,00000000
**Uroc1**	4,48634371	0,0000000	0,00000000
**Epha2**	4,49689807	0,0000000	0,00000000
**Ptpn7**	4,49824623	0,0000005	0,00000007
**Ifit1**	4,51355811	0,0000000	0,00000000
**S100a4**	4,54102047	0,0000000	0,00000000
**Slc18a1**	4,57379479	0,0000000	0,00000000
**Clu**	4,60528406	0,0000000	0,00000000
**Igfbp7**	4,61250747	0,0000000	0,00000000
**Lrrc15**	4,62823665	0,0000000	0,00000000
**Isg20**	4,6850056	0,0000000	0,00000000
**Gbp7**	4,70158864	0,0000000	0,00000000
**Dglucy**	4,70940852	0,0000000	0,00000000
**Gm19705**	4,72903606	0,0000000	0,00000000
**Dysf**	4,75361531	0,0000000	0,00000000
**Ces2e**	4,75536694	0,0000002	0,00000002
**Pkp3**	4,77934965	0,0000000	0,00000000
**Col6a1**	4,80958225	0,0000000	0,00000000
**Ifitm3**	4,816943	0,0000000	0,00000000
**Oas3**	4,83072373	0,0000000	0,00000000
**Ntn4**	4,84610051	0,0000000	0,00000000
**Cd80**	4,87441879	0,0000000	0,00000000
**Ddx60**	4,89702697	0,0000000	0,00000000
**Cemip**	4,95054383	0,0000000	0,00000000
**Pcdh12**	4,96863415	0,0000000	0,00000000
**Oas1g**	4,9741035	0,0000000	0,00000000
**Prss23**	4,97645104	0,0000000	0,00000000
**Ddx58**	5,06649918	0,0000000	0,00000000
**Dnase1l3**	5,11913562	0,0000000	0,00000000
**Syn2**	5,19375074	0,0000000	0,00000000
**Wincr1**	5,19743476	0,0000000	0,00000000
**Ifi204**	5,19934001	0,0000000	0,00000000
**Gbp3**	5,27289901	0,0000000	0,00000000
**S100a6**	5,31854963	0,0000000	0,00000000
**Col1a1**	5,34757117	0,0000000	0,00000000
**Slc6a12**	5,35676257	0,0000000	0,00000000
**Hspb7**	5,38528418	0,0000000	0,00000000
**Tgfbi**	5,39576872	0,0000000	0,00000000
**9530053A07Rik**	5,42628483	0,0000000	0,00000000
**Serpinb2**	5,43400334	0,0000000	0,00000000
**Usp18**	5,52858295	0,0000000	0,00000000
**Xaf1**	5,74293178	0,0000000	0,00000000

**Trim30a**	5,82596206	0,0000000	0,00000000
**Folr2**	5,86887308	0,0000000	0,00000000
**Irf7**	5,90694971	0,0000000	0,00000000
**Ccdc80**	5,9929708	0,0000000	0,00000000
**Irgm2**	6,04934234	0,0000000	0,00000000
**Rtp4**	6,0891282	0,0000000	0,00000000
**Oas2**	6,12990547	0,0000000	0,00000000
**Fgd3**	6,3064106	0,0000000	0,00000000
**Serpine1**	6,32353614	0,0000000	0,00000000
**Isg15**	6,4068244	0,0000000	0,00000000
**Igtp**	6,67842264	0,0000000	0,00000000
**Il11**	6,70096026	0,0000000	0,00000000
**Sncg**	7,35680016	0,0000000	0,00000000
**Tnfrsf23**	7,4369703	0,0000000	0,00000000
**Apol9a**	7,52919892	0,0000000	0,00000000
**Serpinb9b**	7,61596295	0,0000000	0,00000000
**Apol9b**	7,87800091	0,0000000	0,00000000
**Lamc2**	7,90218155	0,0000000	0,00000000

1 MO4 cells exposed to 1µM CM-272 for 24 hours were subjected to RNA sequencing. Gene expression counts were processed using DESeq2 R-package to render normalized expression counts and report on log_2-fold changes with corresponding *p*-values and *q*-values. Listed genes comply with following cut-offs: *p*-value <0.0005, *q*-value <0.002, and |log_2-fold change| >1.

2 log_2-fold change represents CM-272 condition relative to vehicle condition.

Table S2 – RNA sequencing of CM-272-treated MO4 cells *in vitro* - significantly changed gene sets. [1]

Gene set name	# genes	NES	FDR-value	*p*-value
**Epigenetic regulation of gene expression and protein stability**				
Golgi to ER transport				
REACTOME_GOLGI_TO_ER_RETROGRADE_TRANSPORT	125	-1.9019	0.0025	0.0
REACTOME_COPI_DEPENDENT_GOLGI_TO_ERRETROGRADE_TRAFFIC	91	-2.13	0.0001	0.0
REACTOME_KINESINS	53	-2.2377	0.0	0.0
REACTOME_MHC_CLASS_II_ANTIGEN_PRESENTATION	112	-1.4152	0.0914	0.0
REACTOME_INTRA_GOLGI_AND_RETROGRADE_GOLGI_TO_ER_TRAFFIC	191	-1.4671	0.0688	0.0
REACTOME_FACTORS_INVOLVED_IN_MEGAKARYOCYTE_DEVELOPMENT_AND_PLATELET_PRODUCTION	138	-1.9897	0.0009	0.0
REACTOME_RHO_GTPASES_ACTIVATE_IQGAPS	26	-1.7509	0.0099	0.0
DNA modifications				
REACTOME_DNA_METHYLATION	27	-2.4511	0.0	0.0
REACTOME_POSITIVE_EPIGENETIC_REGULATION OF_RRNA_EXPRESSION	68	-2.3641	0.0	0.0
REACTOME_PRC2_METHYLATES_HISTONES_AND_DNA	35	-2.5158	0.0	0.0
REACTOME_EPIGENETIC_REGULATION_OF_GENE EXPRESSION	109	-2.4318	0.0	0.0
Histone modifications				
WP_HISTONE_MODIFICATIONS	44	-1.9623	0.0012	0.0
REACTOME_PKMTS_METHYLATE_HISTONE_LYSINES	49	-2.2776	0.0	0.0
REACTOME_RMTS_METHYLATE_HISTONE_ARGININES	46	-2.0037	0.0008	0.0
REACTOME_CHROMATIN_MODIFYING_ENZYMES	230	-2.0504	0.0004	0.0
REACTOME_HATS_ACETYLATE_HISTONES	99	-2.0788	0.0002	0.0
REACTOME_HDACS_DEACETYLATE_HISTONES	51	-2.1175	0.0001	0.0
mRNA processing				
REACTOME_PROCESSING_OF_CAPPED_INTRONLESS_PRE_MRNA	28	-2.1348	0.0001	0.0
REACTOME_PROCESSING_OF_CAPPED_INTRON_CONTAINING_PRE_MRNA	237	-2.8765	0.0	0.0
REACTOME_MRNA_SPLICING	187	-2.585	0.0	0.0
KEGG_SPLICEOSOME	121	-2.3184	0.0	0.0
REACTOME_MRNA_SPLICING_MINOR_PATHWAY	52	-1.9737	0.0011	0.0
WP_MRNA_PROCESSING	125	-2.633	0.0	0.0
REACTOME_RNA_POLYMERASE_II_TRANSCRIPTION_TERMINATION	65	-2.4017	0.0	0.0
Pyrimidine metabolism				
KEGG_PYRIMIDINE_METABOLISM	94	-1.8542	0.0004	0.0
WP_PYRIMIDINE_METABOLISM	81	-1.7382	0.0109	0.0
RNA degradation				
REACTOME_DEADENYLATION_OF_MRNA	25	-1.9513	0.0014	0.0

REACTOME_DEADENYLATION_DEPENDENT_MRNA_DECAY	55	-2.245	0.0	0.0
KEGG_RNA_DEGRADATION	56	-1.8081	0.0064	0.0
Sumoylation of proteins				
REACTOME_GLUCOSE_METABOLISM	89	-1.4631	0.0702	0.0
REACTOME_HCMV_LATE_EVENTS	68	-2.3627	0.0	0.0
REACTOME_SNRNP_ASSEMBLY	51	-2.6013	0.0	0.0
REACTOME_HIV_LIFE_CYCLE	141	-1.932	0.0018	0.0
REACTOME_TRANSCRIPTIONAL_REGULATION_BY SMALL_RNAS	67	-2.5892	0.0	0.0
REACTOME_INTERACTIONS_OF_VPR_WITH_HOST CELLULAR_PROTEINS	34	-2.3605	0.0	0.0
REACTOME_EXPORT_OF_VIRAL_RIBONUCLEOPROTEINS_FROM_NUCLEUS	30	-2.3538	0.0	0.0
REACTOME_INTERACTIONS_OF_REV_WITH_HOST CELLULAR_PROTEINS	35	-2.4247	0.0	0.0
REACTOME_HCMV_INFECTION	108	-2.2718	0.0	0.0
REACTOME_GLYCOLYSIS	69	-1.7334	0.0113	0.0
REACTOME_TRNA_PROCESSING	107	-2.0594	0.0003	0.0
REACTOME_SUMOYLATION_OF_SUMOYLATION_PROTEINS	33	-2.4211	0.0	0.0
REACTOME_TRANSPORT_OF_MATURE_MRNAS_DERIVED_FROM_INTRONLESS_TRANSCRIPTS	41	-2.4636	0.0	0.0
REACTOME_TRNA_PROCESSING_IN_THE_NUCLEUS	57	-2.3999	0.0	0.0
REACTOME_SUMOYLATION_OF_RNA_BINDING_PROTEINS	44	-2.3122	0.0	0.0
REACTOME_SUMOYLATION_OF_DNA_DAMAGE_RESPONSE_AND_REPAIR_PROTEINS	73	-2.131	0.0001	0.0
REACTOME_POSTMITOTIC_NUCLEAR_PORE_COMPLEX_NPC_REFORMATION	26	-2.369	0.0	0.0
REACTOME_SUMOYLATION_OF_DNA_REPLICATION_PROTEINS	44	-2.5818	0.0	0.0
REACTOME_REGULATION_OF_HSF1_MEDIATED_HEAT_SHOCK_RESPONSE	77	-1.6503	0.0207	0.0
REACTOME_VIRAL_MESSENGER_RNA_SYNTHESIS	42	-2.2042	0.0	0.0
REACTOME_NUCLEAR_PORE_COMPLEX_NPC_DISASSEMBLY	34	-2.5401	0.0	0.0
REACTOME_SUMOYLATION	169	-2.0821	0.0002	0.0
REACTOME_NUCLEAR_IMPORT_OF_REV_PROTEIN	32	-2.4397	0.0	0.0
REACTOME_TRANSPORT_OF_THE_SLBP_DEPENDANT_MATURE_MRNA	34	-2.4215	0.0	0.0
REACTOME_NUCLEAR_ENVELOPE_BREAKDOWN	51	-2.3189	0.0	0.0
REACTOME_NS1_MEDIATED_EFFECTS_ON_HOST_PATHWAYS	38	-1.9847	0.0009	0.0
REACTOME_HCMV_EARLY_EVENTS	87	-2.4343	0.0	0.0
REACTOME_SUMOYLATION_OF_UBIQUITINYLATION_PROTEINS	37	-2.126	0.0001	0.0
REACTOME_GENE_SILENCING_BY_RNA	98	-2.2526	0.0	0.0
REACTOME_TRANSPORT_OF_MATURE_TRANSCRIPT_TO_CYTOPLASM	80	-2.6262	0.0	0.0
REACTOME_SUMOYLATION_OF_CHROMATIN_ORGANIZATION_PROTEINS	55	-2.3872	0.0	0.0

REACTOME_REGULATION_OF_GLUCOKINASE_BY GLUCOKINASE_REGULATORY_PROTEIN	30	-2.2865	0.0	0.0
**Immune recognition and responses**				
Antigen presentation				
KEGG_ANTIGEN_PROCESSING_AND_PRESENTATION	53	1.623	0.0339	0.0023
REACTOME_ANTIGEN_PRESENTATION_FOLDING_ASSEMBLY_AND_PEPTIDE_LOADING_OF_CLASS_I MHC	21	1.6003	0.0408	0.0027
Autoimmune signaling				
KEGG_ALLOGRAFT_REJECTION	27	1.8458	0.0033	0.0
KEGG_VIRAL_ MYOCARDITIS	60	1.6186	0.0349	0.0
WP_ALLOGRAFT_REJECTION	77	1.6921	0.0185	0.0011
KEGG_TYPE_I_DIABETES_MELLITUS	33	1.743	0.0108	0.0
KEGG_GRAFT_VERSUS_HOST_DISEASE	26	1.7866	0.0067	0.0
KEGG_AUTOIMMUNE_THYROID_DISEASE	31	1.8228	0.0041	0.0
Complement system				
REACTOME_COMPLEMENT_CASCADE	58	1.6089	0.0375	0.0023
REACTOME_FORMATION_OF_FIBRIN_CLOT_CLOTTING_CASCADE_	37	1.6206	0.0344	0.0025
WP_COMPLEMENT_AND_COAGULATION_CASCADES	55	1.6055	0.0391	0.0012
KEGG_COMPLEMENT_AND_COAGULATION_CASCADES	64	1.7139	0.0146	0.0
WP_HUMAN_COMPLEMENT_SYSTEM	89	1.8625	0.0022	0.0
Inflammatory response				
REACTOME_INTERLEUKIN_10_SIGNALING	42	1.9482	0.0002	0.0
WP_CYTOKINES_AND_INFLAMMATORY_RESPONSE	26	1.7504	0.0105	0.0013
KEGG_JAK_STAT_SIGNALING_PATHWAY	138	1.5014	0.0926	0.0011
WP_DEVELOPMENT_AND_HETEROGENEITY_OF_THE_ILC_FAMILY	32	1.8309	0.0037	0.0
KEGG_HEMATOPOIETIC_CELL_LINEAGE	78	1.9497	0.0003	0.0
KEGG_CYTOKINE_CYTOKINE_RECEPTOR_INTERACTION	231	1.7192	0.0136	0.0
Interferon signaling				
WP_TYPE_II_INTERFERON_SIGNALING_IFNG	34	1.8623	0.0021	0.0
WP_THE_HUMAN_IMMUNE_RESPONSE_TO_TUBERCULOSIS	22	2.0883	0.0	0.0
REACTOME_INTERFERON_GAMMA_SIGNALING	76	2.0867	0.0	0.0
REACTOME_INTERFERON_ALPHA_BETA_SIGNALING	52	2.3095	0.0	0.0
REACTOME_INTERFERON_SIGNALING	166	1.9177	0.0009	0.0
Interleukin pathways				
PID_IL12_2PATHWAY	61	1.6012	0.0406	0.0
PID_IL27_PATHWAY	26	1.5955	0.0418	0.0
REACTOME_INTERLEUKIN_4_AND_INTERLEUKIN_13_SIGNALING	109	1.8759	0.0021	0.0
PID_IL23_PATHWAY	37	1.7323	0.0112	0.0039
TGF-β signaling				
WP_HYPOTHESIZED_PATHWAYS_IN_PATHOGENESIS_OF_CARDIOVASCULAR_DISEASE	24	1.7069	0.0153	0.0013
WP_TGFBETA_RECEPTOR_SIGNALING	54	1.6506	0.0264	0.0

WP_TGFBETA_RECEPTOR_SIGNALLING_IN_SKELETAL_DYSPLASIAS	58	1.6447	0.0276	0.0
Toll-like receptor signaling				
KEGG_TOLL_LIKE_RECEPTOR_SIGNALING_PATHWAY	88	1.5516	0.0661	0.0022
WP_REGULATION_OF_TOLLLIKE_RECEPTOR_SIGNALING_PATHWAY	128	1.5392	0.0716	0.0
WP_TOLLLIKE_RECEPTOR_SIGNALING_PATHWAY	90	1.5144	0.084	0.0022
REACTOME_TRAF6_MEDIATED_IRF7_ACTIVATION	18	1.6801	0.0209	0.0027
WP_TOLLLIKE_RECEPTOR_SIGNALING_RELATED_TO_MYD88	31	1.6168	0.0349	0.0025
WP_SARS_CORONAVIRUS_AND_INNATE_IMMUNITY	20	1.7346	0.0109	0.0027
WP_MIRNAS_INVOLVEMENT_IN_THE_IMMUNE_RESPONSE_IN_SEPSIS	60	1.5995	0.0406	0.0023
WP_HOSTPATHOGEN_INTERACTION_OF_HUMAN_CORONA_VIRUSES_INTERFERON_INDUCTION	33	1.7904	0.0065	0.0
WP_TYPE_I_INTERFERON_INDUCTION_AND_SIGNALING_DURING_SARSCOV2_INFECTION	29	1.7061	0.0153	0.0026
WP_FIBRIN_COMPLEMENT_RECEPTOR_3_SIGNALING_PATHWAY	41	1.6355	0.0293	0.0012
**Carcinogenesis-related pathways**				
Cell cycle				
REACTOME_RHO_GTPASE_EFFECTORS	274	-2.6005	0.0	0.0
REACTOME_CELL_CYCLE_CHECKPOINTS	265	-3.1369	0.0	0.0
REACTOME_ANCHORING_OF_THE_BASAL_BODY_TO_THE_PLASMA_MEMBRANE	96	-2.4938	0.0	0.0
REACTOME_RECRUITMENT_OF_NUMA_TO_MITOTIC_CENTROSOMES	87	-2.4848	0.0	0.0
REACTOME_INHIBITION_OF_DNA_RECOMBINATION_AT_TELOMERE	38	-2.1621	0.0001	0.0
REACTOME_INHIBITION_OF_THE_PROTEOLYTIC_ACTIVITY_OF_APC_C_REQUIRED_FOR_THE_ONSET_OF_ANAPHASE_BY_MITOTIC_SPINDLE_CHECKPOINT_COMPONENTS	21	-2.0011	0.0008	0.0
REACTOME_CYCLIN_A_B1_B2_ASSOCIATED_EVENTS_DURING_G2_M_TRANSITION	25	-2.6381	0.0	0.0
REACTOME_RUNX1_REGULATES_GENES_INVOLVED_IN_MEGAKARYOCYTE_DIFFERENTIATION_AND_PLATELET_FUNCTION	59	-1.6988	0.0145	0.0
PID_E2F_PATHWAY	72	-2.2036	0.0	0.0
REACTOME_INITIATION_OF_NUCLEAR_ENVELOPENE_REFORMATION	19	-2.069	0.0003	0.0
REACTOME_FORMATION_OF_THE_BETA_CATENIN_TCF_TRANSACTIVATING_COMPLEX	53	-2.2427	0.0	0.0
WP_DNA_IRDOUBLE_STRAND_BREAKS_DSBS_AND_CELLULAR_RESPONSE_VIA_ATM	55	-2.5294	0.0	0.0
REACTOME_TELOMERE_C_STRAND_LAGGING_STRAND_SYNTHESIS	33	-2.6074	0.0	0.0
PID_ATM_PATHWAY	34	-2.2806	0.0	0.0
REACTOME_REPRODUCTION	104	-2.3235	0.0	0.0
REACTOME_ORGANELLE_BIOGENESIS_AND_MAINTENANCE	288	-1.7125	0.0132	0.0

REACTOME_SENESCENCE_ASSOCIATED_SECRETORY_PHENOTYPE_SASP_	72	-1.9527	0.0014	0.0
REACTOME_POLYMERASE_SWITCHING_ON_THE_C_STRAND_OF_THE_TELOMERE	25	-2.5368	0.0	0.0
REACTOME_B_WICH_COMPLEX_POSITIVELY_REGULATES_RRNA_EXPRESSION	53	-2.2701	0.0	0.0
REACTOME_MITOTIC_G2_G2_M_PHASES	191	-2.4314	0.0	0.0
REACTOME_ACTIVATION_OF_ANTERIOR_HOX_GENES_IN_HINDBRAIN_DEVELOPMENT_DURING_EARLY_EMBRYOGENESIS	84	-2.2113	0.0	0.0
REACTOME_CHROMOSOME_MAINTENANCE	109	-3.1124	0.0	0.0
REACTOME_TELOMERE_MAINTENANCE	83	-2.8198	0.0	0.0
PID_FANCONI_PATHWAY	46	-2.7261	0.0	0.0
REACTOME_TRANSLESION_SYNTHESIS_BY_Y_FAMILY_DNA_POLYMERASES_BYPASSES_LESIONS_ON_DNA_TEMPLATE	39	-1.7728	0.0083	0.0
REACTOME_ERCC6_CSB_AND_EHMT2_G9A_POSITIVELY_REGULATE_RRNA_EXPRESSION	38	-2.4181	0.0	0.0
REACTOME_MITOTIC_PROPHASE	102	-2.6825	0.0	0.0
REACTOME_REGULATION_OF_TP53_ACTIVITY	158	-2.0434	0.0004	0.0
WP_RETINOBLASTOMA_GENE_IN_CANCER	86	-3.1195	0.0	0.0
REACTOME_CYCLIN_A_CDK2_ASSOCIATED_EVENTS_AT_S_PHASE_ENTRY	85	-1.5635	0.0371	0.0
REACTOME_G2_M_DNA_DAMAGE_CHECKPOINT	70	-2.8504	0.0	0.0
REACTOME_NEGATIVE_EPIGENETIC_REGULATION_OF_RRNA_EXPRESSION	70	-2.3514	0.0	0.0
REACTOME_RECRUITMENT_OF_MITOTIC_CENTROSOME_PROTEINS_AND_COMPLEXES	78	-2.496	0.0	0.0
REACTOME_CONVERSION_FROM_APC_C_CDC20_TO_APC_C_CDH1_IN_LATE_ANAPHASE	20	-1.8448	0.0044	0.0042
REACTOME_TERMINATION_OF_TRANSLESION_DNA_SYNTHESIS	32	-1.7089	0.0134	0.0
PID_ATR_PATHWAY	39	-2.8735	0.0	0.0
REACTOME_TRANSCRIPTION_OF_E2F_TARGETS_UNDER_NEGATIVE_CONTROL_BY_P107_RBL1_AND_P130_RBL2_IN_COMPLEX_WITH_HDAC1	16	-2.3997	0.0	0.0
KEGG_OOCYTE_MEIOSIS	107	-2.0208	0.0006	0.0
REACTOME_SEPARATION_OF_SISTER_CHROMATIDS	183	-2.8433	0.0	0.0
REACTOME_REGULATION_OF_TP53_ACTIVITY_THROUGH_PHOSPHORYLATION	91	-2.2074	0.0	0.0
REACTOME_LAGGING_STRAND_SYNTHESIS	20	-2.6226	0.0	0.0
REACTOME_AURKA_ACTIVATION_BY_TPX2	71	-2.6781	0.0	0.0
PID_PLK1_PATHWAY	46	-2.9439	0.0	0.0
REACTOME_PHOSPHORYLATION_OF_THE_APC_C	20	-1.9603	0.0012	0.0
REACTOME_SWITCHING_OF_ORIGINS_TO_A_POST REPLICATIVE_STATE	91	-2.054	0.0004	0.0
WP_DNA_IRDAMAGE_AND_CELLULAR_RESPONSE VIA_ATR	80	-2.9654	0.0	0.0
REACTOME_G1_S_SPECIFIC_TRANSCRIPTION	28	-2.7196	0.0	0.0
REACTOME_DUAL_INCISION_IN_GG_NER	40	-2.3625	0.0	0.0
REACTOME_PROCESSIVE_SYNTHESIS_ON_THE_C_STRAND_OF_THE_TELOMERE	18	-2.1099	0.0001	0.0

REACTOME_ACTIVATION_OF_ATR_IN_RESPONSE_TO_REPLICATION_STRESS	37	-2.8539	0.0	0.0
KEGG_DNA_REPLICATION	35	-2.9181	0.0	0.0
REACTOME_DUAL_INCISION_IN_TC_NER	63	-2.0551	0.0004	0.0
REACTOME_E2F_MEDIATED_REGULATION_OF_DNA_REPLICATION	22	-2.2966	0.0	0.0
REACTOME_TRANSCRIPTION_OF_E2F_TARGETS_UNDER_NEGATIVE_CONTROL_BY_DREAM_COMPLEX	19	-2.3834	0.0	0.0
KEGG_PROGESTERONE_MEDIATED_OOCYTE_MATURATION	83	-1.9003	0.0025	0.0
REACTOME_TRANSLESION_SYNTHESIS_BY_POLK	17	-2.0197	0.0006	0.0
REACTOME_TRANSLESION_SYNTHESIS_BY_POLH	19	-2.1091	0.0001	0.0
REACTOME_RNA_POLYMERASE_I_TRANSCRIPTION	72	-2.2326	0.0	0.0
WP_G1_TO_S_CELL_CYCLE_CONTROL	64	-2.4889	0.0	0.0
REACTOME_RHO_GTPASES_ACTIVATE_FORMINS	132	-3.0313	0.0	0.0
REACTOME_RESOLUTION_OF_AP_SITES_VIA_THE MULTIPLE_NUCLEOTIDE_PATCH_REPLACEMENT_PATHWAY	24	-2.5118	0.0	0.0
REACTOME_DISEASES_OF_MITOTIC_CELL_CYCLE	36	-1.7759	0.0082	0.0
REACTOME_HDR_THROUGH_SINGLE_STRAND_ANNEALING_SSA_	37	-2.5529	0.0	0.0
WP_DNA_MISMATCH_REPAIR	23	-2.5945	0.0	0.0
REACTOME_RESOLUTION_OF_D_LOOP_STRUCTURES	33	-2.4205	0.0	0.0
REACTOME_M_PHASE	364	-2.9423	0.0	0.0
REACTOME_DNA_DAMAGE_TELOMERE_STRESS_INDUCED_SENESCENCE	50	-2.3346	0.0	0.0
REACTOME_EXTENSION_OF_TELOMERES	50	-2.7375	0.0	0.0
REACTOME_G0_AND_EARLY_G1	27	-2.6346	0.0	0.0
REACTOME_PROCESSIVE_SYNTHESIS_ON_THE_LAGGING_STRAND	15	-2.4115	0.0	0.0
REACTOME_RESOLUTION_OF_ABASIC_SITES_AP_SITES_	37	-2.5524	0.0	0.0
REACTOME_MITOTIC_G1_PHASE_AND_G1_S_TRANSITION	147	-2.7303	0.0	0.0
REACTOME_HDR_THROUGH_HOMOLOGOUS_RECOMBINATION_HRR_	66	-2.7893	0.0	0.0
REACTOME_MEIOTIC_SYNAPSIS	49	-2.3809	0.0	0.0
REACTOME_MEIOSIS	79	-2.5991	0.0	0.0
REACTOME_POLO_LIKE_KINASE_MEDIATED_EVENTS	16	-2.49	0.0	0.0
REACTOME_ACTIVATION_OF_THE_PRE_REPLICATIVE_COMPLEX	32	-2.9215	0.0	0.0
REACTOME_RECOGNITION_AND_ASSOCIATION_OF_DNA_GLYCOSYLASE_WITH_SITE_CONTAINING_AN_AFFECTED_PURINE	26	-2.481	0.0	0.0
REACTOME_APC_CDC20_MEDIATED_DEGRADATION_OF_NEK2A	26	-2.1708	0.0001	0.0
REACTOME_SIRT1_NEGATIVELY_REGULATES_RRNA_EXPRESSION	30	-2.4826	0.0	0.0
REACTOME_OXIDATIVE_STRESS_INDUCED_SENESCENCE	87	-2.1904	0.0	0.0

REACTOME_G2_M_CHECKPOINTS	143	-2.7554	0.0	0.0
REACTOME_TRANSCRIPTIONAL_REGULATION_OF GRANULOPOIESIS	52	-2.2394	0.0	0.0
REACTOME_TRANSCRIPTION_COUPLED_NUCLEO TIDE_EXCISION_REPAIR_TC_NER_	76	-2.1176	0.0001	0.0
REACTOME_CILIUM_ASSEMBLY	195	-1.8208	0.0058	0.0
KEGG_HOMOLOGOUS_RECOMBINATION	27	-2.5111	0.0	0.0
REACTOME_CYCLIN_D_ASSOCIATED_EVENTS_IN_ G1	47	-1.7167	0.0129	0.0
REACTOME_NUCLEAR_ENVELOPE_NE_REASSEMB LY	67	-2.4833	0.0	0.0
REACTOME_MITOTIC_METAPHASE_AND_ANAPHA SE	225	-2.9416	0.0	0.0
REACTOME_MITOTIC_PROMETAPHASE	193	-3.2758	0.0	0.0
REACTOME_APC_C_CDC20_MEDIATED_DEGRADA TION_OF_CYCLIN_B	24	-2.0514	0.0004	0.0
WP_REGULATION_OF_SISTER_CHROMATID_SEPA RATION_AT_THE_METAPHASEANAPHASE_TRANSI TION	15	-2.1792	0.0001	0.0
WP_CELL_CYCLE	119	-2.5818	0.0	0.0
REACTOME_ASSEMBLY_OF_THE_PRE_REPLICATI VE_COMPLEX	68	-1.8755	0.0032	0.0
REACTOME_RESOLUTION_OF_SISTER_CHROMATI D_COHESION	118	-3.3488	0.0	0.0
REACTOME_RHO_GTPASES_ACTIVATE_PKNS	53	-1.973	0.0011	0.0
REACTOME_CELLULAR_SENESCENCE	155	-2.1188	0.0001	0.0
REACTOME_FANCONI_ANEMIA_PATHWAY	37	-2.1185	0.0001	0.0
REACTOME_ESTROGEN_DEPENDENT_GENE_EXPR ESSION	111	-1.7563	0.0094	0.0
KEGG_CELL_CYCLE	123	-2.63	0.0	0.0
REACTOME_CONDENSATION_OF_PROPHASE_CHR OMOSOMES	35	-2.6805	0.0	0.0
REACTOME_RUNX1_REGULATES_TRANSCRIPTION OF_GENES_INVOLVED_IN_DIFFERENTIATION_OF_ HSCS	92	-1.5984	0.0296	0.0
REACTOME_DNA_REPLICATION	127	-2.7513	0.0	0.0
REACTOME_REGULATION_OF_PLK1_ACTIVITY_A T_G2_M_TRANSITION	86	-2.6285	0.0	0.0
REACTOME_RNA_POLYMERASE_I_PROMOTER_ES CAPE	52	-2.2517	0.0	0.0
REACTOME_PRE_NOTCH_EXPRESSION_AND_PROC ESSING	82	-1.6885	0.0156	0.0
REACTOME_GAP_FILLING_DNA_REPAIR_SYNTHES IS_AND_LIGATION_IN_GG_NER	25	-2.4277	0.0	0.0
WP_BASE_EXCISION_REPAIR	31	-2.5328	0.0	0.0
REACTOME_GLOBAL_GENOME_NUCLEOTIDE_EX CISION_REPAIR_GG_NER_	83	-2.1227	0.0001	0.0
REACTOME_DNA_REPLICATION_PRE_INITIATION	84	-2.4054	0.0	0.0
KEGG_MISMATCH_REPAIR	22	-2.559	0.0	0.0
REACTOME_MITOTIC_SPINDLE_CHECKPOINT	109	-3.2107	0.0	0.0
REACTOME_SIGNALING_BY_RHO_GTPASES	402	-2.3289	0.0	0.0
REACTOME_DNA_STRAND_ELONGATION	32	-3.0646	0.0	0.0

REACTOME_APC_C_MEDIATED_DEGRADATION_OF_CELL_CYCLE_PROTEINS	88	-1.9093	0.0023	0.0
REACTOME_MEIOTIC_RECOMBINATION	48	-2.6358	0.0	0.0
REACTOME_ORC1_REMOVAL_FROM_CHROMATIN	71	-1.7797	0.008	0.0
REACTOME_RESOLUTION_OF_D_LOOP_STRUCTURES_THROUGH_SYNTHESIS_DEPENDENT_STRAND_ANNEALING_SDSA_	26	-2.4311	0.0	0.0
REACTOME_ACTIVATED_PKN1_STIMULATES_TRANSCRIPTION_OF_AR_ANDROGEN_RECEPTOR_REGULATED_GENES_KLK2_AND_KLK3	27	-2.4351	0.0	0.0
WP_DNA_REPLICATION	41	-2.967	0.0	0.0
PID_BARD1_PATHWAY	29	-2.5673	0.0	0.0
REACTOME_S_PHASE	162	-2.7695	0.0	0.0
REACTOME_DEPOSITION_OF_NEW_CENPA_CONTAINING_NUCLEOSOMES_AT_THE_CENTROMERE	44	-2.8681	0.0	0.0
DNA damage response				
WP_MIRNA_REGULATION_OF_DNA_DAMAGE_RESPONSE	88	-1.8351	0.0049	0.0
WP_DNA_DAMAGE_RESPONSE	66	-1.8157	0.006	0.0
WP_ATM_SIGNALING_PATHWAY	40	-2.0345	0.0005	0.0
WP_INTEGRATED_CANCER_PATHWAY	44	-1.8389	0.0048	0.0
KEGG_NUCLEOTIDE_EXCISION_REPAIR	42	-2.4169	0.0	0.0
REACTOME_DNA_DAMAGE_BYPASS	48	-1.8898	0.0028	0.0
REACTOME_NONHOMOLOGOUS_END_JOINING_NHEJ_	44	-2.4834	0.0	0.0
REACTOME_HOMOLOGOUS_DNA_PAIRING_AND_STRAND_EXCHANGE	42	-2.7215	0.0	0.0
REACTOME_PCNA_DEPENDENT_LONG_PATCH_BASE_EXCISION_REPAIR	21	-2.5942	0.0	0.0
REACTOME_NUCLEOTIDE_EXCISION_REPAIR	108	-2.1	0.0001	0.0
REACTOME_DNA_REPAIR	296	-2.6978	0.0	0.0
KEGG_BASE_EXCISION_REPAIR	33	-2.169	0.0001	0.0
REACTOME_BASE_EXCISION_REPAIR	60	-2.8635	0.0	0.0
REACTOME_DNA_DOUBLE_STRAND_BREAK_RESPONSE	52	-2.4418	0.0	0.0
REACTOME_BASE_EXCISION_REPAIR_AP_SITE_FORMATION	33	-2.4868	0.0	0.0
REACTOME_MISMATCH_REPAIR	15	-2.2441	0.0	0.0
REACTOME_HOMOLOGY_DIRECTED_REPAIR	112	-3.046	0.0	0.0
REACTOME_RECOGNITION_OF_DNA_DAMAGE_BY_PCNA_CONTAINING_REPLICATION_COMPLEX	30	-2.4868	0.0	0.0
WP_NUCLEOTIDE_EXCISION_REPAIR	42	-2.4755	0.0	0.0
REACTOME_DNA_DOUBLE_STRAND_BREAK_REPAIR	140	-3.0429	0.0	0.0
REACTOME_PROCESSING_OF_DNA_DOUBLE_STRAND_BREAK_ENDS	73	-2.8438	0.0	0.0
ECM metabolism				
WP_MATRIX_METALLOPROTEINASES	29	1.6362	0.0292	0.0038
PID_INTEGRIN5_PATHWAY	17	1.6712	0.023	0.0
KEGG_FOCAL_ADHESION	195	1.6641	0.0237	0.0
REACTOME_LAMININ_INTERACTIONS	30	1.8015	0.0057	0.0
REACTOME_MET_ACTIVATES_PTK2_SIGNALING	30	1.6404	0.0282	0.0026
PID_INTEGRIN1_PATHWAY	66	1.9123	0.0009	0.0

REACTOME_COLLAGEN_CHAIN_TRIMERIZATION	43	1.6418	0.0278	0.0012
REACTOME_INTEGRIN_CELL_SURFACE_INTERACTIONS	84	1.7676	0.0081	0.0
REACTOME_ANCHORING_FIBRIL_FORMATION	15	1.8935	0.0015	0.0
WP_ALPHA_6_BETA_4_SIGNALING_PATHWAY	33	1.5979	0.0409	0.0026
REACTOME_COLLAGEN_FORMATION	89	1.8363	0.0038	0.0
PID_AVB3_OPN_PATHWAY	31	1.6701	0.0232	0.0012
REACTOME_DEGRADATION_OF_THE_EXTRACELLULAR_MATRIX	134	1.8377	0.0039	0.0
PID_INTEGRIN2_PATHWAY	28	1.7013	0.0166	0.0013
REACTOME_COLLAGEN_BIOSYNTHESIS_AND_MODIFYING_ENZYMES	66	1.5997	0.0408	0.0023
REACTOME_ASSEMBLY_OF_COLLAGEN_FIBRILS_AND_OTHER_MULTIMERIC_STRUCTURES	61	1.8358	0.0037	0.0
REACTOME_ECM_PROTEOGLYCANS	76	1.7397	0.0108	0.0
REACTOME_EXTRACELLULAR_MATRIX_ORGANIZATION	291	1.7677	0.0083	0.0
REACTOME_COLLAGEN_DEGRADATION	63	1.7591	0.0094	0.0
REACTOME_NON_INTEGRIN_MEMBRANE_ECM_INTERACTIONS	59	1.773	0.0077	0.0
KEGG_ECM_RECEPTOR_INTERACTION	83	1.637	0.0292	0.0
PID_AVB3_INTEGRIN_PATHWAY	74	1.6628	0.0234	0.0
PID_INTEGRIN3_PATHWAY	43	1.9161	0.0008	0.0
PID_SYNDECAN_1_PATHWAY	46	1.5358	0.0734	0.0048
PID_UPA_UPAR_PATHWAY	42	1.7766	0.0075	0.0
WP_FOCAL_ADHESION	195	1.6186	0.0352	0.0
Keratinization				
REACTOME_FORMATION_OF_THE_CORNIFIED_ENVELOPE	99	1.6139	0.0361	0.0
REACTOME_KERATINIZATION	130	1.6896	0.019	0.0
NCAM signaling				
REACTOME_NCAM1_INTERACTIONS	42	1.7417	0.0108	0.0
REACTOME_NCAM_SIGNALING_FOR_NEURITE_OUT_GROWTH	63	1.7358	0.0108	0.0
PPAR signaling				
KEGG_PPAR_SIGNALING_PATHWAY	68	1.5814	0.0491	0.0035
WP_PPAR_SIGNALING_PATHWAY	67	1.618	0.0349	0.0035
TP53 regulation				
REACTOME_TP53_REGULATES_TRANSCRIPTION_OF_CELL_CYCLE_GENES	48	-1.9636	0.0012	0.0
REACTOME_TRANSCRIPTIONAL_REGULATION_BY TP53	350	-1.7507	0.0099	0.0
PID_P53_DOWNSTREAM_PATHWAY	131	1.4861	0.0998	0.0021
REACTOME_TP53_REGULATES_TRANSCRIPTION_OF_GENES_INVOLVED_IN_G2_CELL_CYCLE_ARREST	17	-1.9329	0.0018	0.0
**Others**				
Adipogenesis				
WP_ADIPOGENESIS	129	1.7206	0.0136	0.0
WP_TRANSCRIPTION_FACTOR_REGULATION_IN_ADIPOGENESIS	22	1.8038	0.0055	0.0
Cardiomyopathy				
KEGG_HYPERTROPHIC_CARDIOMYOPATHY_HCM	83	1.6682	0.0232	0.0

KEGG_DILATED_CARDIOMYOPATHY	89	1.6431	0.0277	0.0011
KEGG_ARRHYTHMOGENIC_RIGHT_VENTRICULAR CARDIOMYOPATHY_ARVC	74	1.5454	0.0682	0.0011
WP_ARRHYTHMOGENIC_RIGHT_VENTRICULAR_CARDIOMYOPATHY	74	1.5328	0.074	0.0034
Fatty acid metabolism				
REACTOME_ARACHIDONIC_ACID_METABOLISM	53	1.663	0.0236	0.0
WP_EICOSANOID_SYNTHESIS	22	1.6724	0.0229	0.0027
WP_EICOSANOID_METABOLISM_VIA_LIPO_OXYGENASES_LOX	28	1.6988	0.0171	0.0013
REACTOME_FATTY_ACID_METABOLISM	168	1.5306	0.0756	0.0
Glutathione/cytochrome p450 metabolism				
WP_GLUTATHIONE_METABOLISM	19	1.6734	0.0228	0.0028
REACTOME_PHASE_II_CONJUGATION_OF_COMPOUNDS	86	1.7161	0.0141	0.0
KEGG_METABOLISM_OF_XENOBIOTICS_BY_CYTOCHROME_P450	48	1.9572	0.0002	0.0
WP_METAPATHWAY_BIOTRANSFORMATION_PHASE_I_AND_II	149	1.6565	0.0249	0.0
REACTOME_PHASE_I_FUNCTIONALIZATION_OF_COMPOUNDS	93	1.6458	0.0276	0.0
REACTOME_CYTOCHROME_P450_ARRANGED_BY_SUBSTRATE_TYPE	55	1.5261	0.0781	0.0048
KEGG_RETINOL_METABOLISM	44	1.7199	0.0136	0.0
REACTOME_BIOLOGICAL_OXIDATIONS	184	1.7924	0.0065	0.0
KEGG_GLUTATHIONE_METABOLISM	46	1.5179	0.0812	0.0012
REACTOME_GLUTATHIONE_CONJUGATION	31	1.8742	0.002	0.0
KEGG_DRUG_METABOLISM_CYTOCHROME_P450	50	1.925	0.0006	0.0
Muscle contraction				
REACTOME_ION_HOMEOSTASIS	52	1.723	0.0132	0.0
REACTOME_SMOOTH_MUSCLE_CONTRACTION	36	2.0309	0.0	0.0
WP_STRIATED_MUSCLE_CONTRACTION_PATHWAY	38	1.7136	0.0145	0.005
REACTOME_MUSCLE_CONTRACTION	200	1.866	0.002	0.0
REACTOME_CARDIAC_CONDUCTION	134	1.6643	0.0239	0.0

[1] MO4 cells exposed to 1μM CM-272 for 24 hours were subjected to RNA sequencing, followed by GSEA on normalized gene expression counts. Listed gene sets comply with following cut-offs: nominal *p*-value <0.005, FDR-value <0.1, and |NES| >1.

Table S3 – Multiplex gene expression analysis on *ex vivo* tumor tissue - significantly changed gene sets. [1]

Gene set name	# genes	NES	FDR-value	*p*-value
**Immune response**				
Toll-like receptor signaling				
REACTOME_MYD88_INDEPENDENT_TLR4_CASCADE_	16	2.3143	0.0	0.0
REACTOME_TOLL_LIKE_RECEPTOR_4_TLR4_CASCADE	20	2.223	0.0005	0.0
REACTOME_TOLL_LIKE_RECEPTOR_CASCADES	22	2.1434	0.0003	0.0
REACTOME_TOLL_LIKE_RECEPTOR_TLR1_TLR2_CASCADE	16	2.0911	0.0008	0.0
WP_CYTOSOLIC_DNASENSING_PATHWAY	16	1.9938	0.0025	0.0016
WP_NOVEL_INTRACELLULAR_COMPONENTS_OF_RIGILIKE_RECEPTOR_RLR_PATHWAY	15	1.934	0.0042	0.0
KEGG_RIG_I_LIKE_RECEPTOR_SIGNALING_PATHWAY	16	1.8374	0.0088	0.00
KEGG_TOLL_LIKE_RECEPTOR_SIGNALING_PATHWAY	23	1.7758	0.0122	0.0046
WP_TOLLLIKE_RECEPTOR_SIGNALING_PATHWAY	23	1.7679	0.0116	0.0046
IFN-I response				
REACTOME_DDX58_IFIH1_MEDIATED_INDUCTION_OF_INTERFERON_ALPHA_BETA	17	2.0716	0.0008	0.0
T cell receptor signaling				
WP_TCELL_ANTIGEN_RECEPTOR_TCR_SIGNALING_PATHWAY	24	1.7989	0.0104	0.0
**Other pathways**				
Signaling by nuclear receptors				
REACTOME_SIGNALING_BY_NUCLEAR_RECEPTORS	17	1.9016	0.0051	0.0016
Cancer-related pathways				
WP_INTEGRATED_BREAST_CANCER_PATHWAY	15	1.961	0.0035	0.0
REACTOME_TRANSCRIPTIONAL_REGULATION_BY_TP53	16	1.8904	0.0054	0.0047
WP_GASTRIN_SIGNALING_PATHWAY	16	1.8744	0.0061	0.0015
KEGG_APOPTOSIS	15	1.8212	0.0096	0.015
KEGG_PATHWAYS_IN_CANCER	32	1.8003	0.0105	0.0015
KEGG_APOPTOSIS	15	1.8212	0.0096	0.015

[1] Tumors of MO4 tumor-bearing mice subjected to the CM-272 treatment regimen were resected at 706.9±193.8 mm^3 tumor volume and subjected to multiplex gene expression analysis, followed by GSEA on normalized gene expression counts. Listed gene sets comply with following cut-offs: nominal *p*-value <0.005, FDR-value <0.1, and |NES| >1.

Table S4 – Report on statistical significance pertaining to Figure 5C. [1]

Sidak multiple comparison test \ Days after tumor inoculation	7	10	11	12	13	14	17	18	19
vehicle vs CM-272	-	-	-	-	-	-			
vehicle vs DC	-	-	*	*	**	*			
vehicle vs DC + CM-272	*	**	**	**	***	***			
CM-272 vs DC	-	-	-	-	-	-	***		
CM-272 vs DC + CM-272	-	-	*	*	**	**	****		
DC vs DC + CM-272	-	-	-	-	-	-	-	-	-

[1] MO4 tumor-bearing C57Bl6J mice were subjected to following treatment regimen: vehicle treatment, CM-272 treatment, vehicle treatment + DC vaccination, or CM-272 treatment + DC vaccination, until 1000 mm^3 tumor volume endpoint was reached. Mean tumor volume in time was plotted for each experimental group, until the first mouse of the concerning group had reached the experimental endpoint tumor volume (Figure 5C). Experimental conditions were statistically compared using REML modeling with Geisser-Greenhouse correction and post-hoc Sidak multiple comparison test. Fixed effects of 'treatment' was significant ($p<0.0001$). Adjusted *p*-values (*q*-values) of the Sidak multiple comparison tests are listed in this table. Asterisks indicate statistical significance: * $q \leq 0.05$; ** $q \leq 0.01$; *** $q \leq 0.001$; **** $q \leq 0.0001$.
